# Supplementary material for: zUMIs - A fast and flexible pipeline to process RNA sequencing data with UMIs
Source: Gigascience. 2018 May 26;7(6):giy059. doi: 10.1093/gigascience/giy059 (PMC6007394; doi:10.1093/gigascience/giy059)
Supplement: GIGA-D-17-00271_Original_Submission.pdf [file giy059_giga-d-17-00271_original_submission.pdf]

GigaScience

zUMIs - A fast and flexible pipeline to process RNA sequencing data with UMIs

--Manuscript Draft--

**Manuscript Number:**	GIGA-D-17-00271
**Full Title:**	zUMIs - A fast and flexible pipeline to process RNA sequencing data with UMIs
**Article Type:**	Technical Note
**Funding Information:**	Deutsche Forschungsgemeinschaft (SFB1243 - A15) — Dr. Ines Hellmann<br>Deutsche Forschungsgemeinschaft (SFB1243 - A14) — Prof. Wolfgang Enard
**Abstract:**	Single cell RNA-seq (scRNA-seq) experiments typically analyze hundreds or thousands of cells after amplification of the cDNA. The high throughput is made possible by the early introduction of sample-specific barcodes (BCs) and the amplification bias is alleviated by unique molecular identifiers (UMIs). Thus the ideal analysis pipeline for scRNA-seq data needs to efficiently tabulate reads according to both BC and UMI.<br>zUMIs is such a pipeline, it can handle both known and random BCs and also efficiently collapses UMIs, either just for exon mapping reads or for both exon and intron mapping reads. Another unique feature of zUMIs is the adaptive downsampling function, that facilitates dealing with hugely varying library sizes, but also allows to evaluate whether the library has been sequenced to saturation. zUMIs flexibility allows to accommodate data generated with any of the major scRNA-seq protocols that use BCs and UMIs. To illustrate the utility of zUMIs, we analysed a single-nucleus RNA-seq dataset and show that more than 35% of all reads map to introns. We furthermore show that these intronic reads are informative about expression levels, significantly increasing the number of detected genes and improving the cluster resolution.
**Corresponding Author:**	Ines Hellmann<br>Ludwig-Maximilians-Universitat Munchen Fakultat fur Biologie<br>Martinsried, GERMANY
**Corresponding Author Secondary Information:**	
**Corresponding Author's Institution:**	Ludwig-Maximilians-Universitat Munchen Fakultat fur Biologie
**Corresponding Author's Secondary Institution:**	
**First Author:**	Swati Parekh
**First Author Secondary Information:**	
**Order of Authors:**	Swati Parekh<br>Christoph Ziegenhain<br>Beate Vieth<br>Wolfgang Enard<br>Ines Hellmann
**Order of Authors Secondary Information:**	
**Opposed Reviewers:**	
**Additional Information:**	
**Question**	**Response**
Are you submitting this manuscript to a special series or article collection?	No
**Experimental design and statistics**	Yes

Powered by Editorial Manager® and ProduXion Manager® from Aries Systems Corporation

Full details of the experimental design and statistical methods used should be given in the Methods section, as detailed in our Minimum Standards Reporting Checklist. Information essential to interpreting the data presented should be made available in the figure legends.<br><br>Have you included all the information requested in your manuscript?	
**Resources**<br><br>A description of all resources used, including antibodies, cell lines, animals and software tools, with enough information to allow them to be uniquely identified, should be included in the Methods section. Authors are strongly encouraged to cite Research Resource Identifiers (RRIDs) for antibodies, model organisms and tools, where possible.<br><br>Have you included the information requested as detailed in our Minimum Standards Reporting Checklist?	Yes
**Availability of data and materials**<br><br>All datasets and code on which the conclusions of the paper rely must be either included in your submission or deposited in publicly available repositories (where available and ethically appropriate), referencing such data using a unique identifier in the references and in the "Availability of Data and Materials" section of your manuscript.<br><br>Have you have met the above requirement as detailed in our Minimum Standards Reporting Checklist?	Yes

Manuscript Click here to download Manuscript main.tex

Placeholder for OUP logo oup.pdf

Placeholder for journal logo gigascience-logo.pdf

GigaScience, 2017, 1–6

doi: xx.xxxx/xxxx
Manuscript in Preparation
Technical Note

TECHNICAL NOTE

zUMIs – A fast and flexible pipeline to process RNA sequencing data with UMIs

Swati Parekh[1,*,†], Christoph Ziegenhain[1,†], Beate Vieth[1], Wolfgang Enard[1] and Ines Hellmann[1,*]

[1]Anthropology & Human Genomics, Department of Biology II, Ludwig-Maximilians University, 82152 Martinsried, Germany

[*]parekh@bio.lmu.de; hellmann@bio.lmu.de
[†]Contributed equally.

Abstract

Single cell RNA-seq (scRNA-seq) experiments typically analyze hundreds or thousands of cells after amplification of the cDNA. The high throughput is made possible by the early introduction of sample-specific barcodes (BCs) and the amplification bias is alleviated by unique molecular identifiers (UMIs). Thus the ideal analysis pipeline for scRNA-seq data needs to efficiently tabulate reads according to both BC and UMI. *zUMIs* is such a pipeline, it can handle both known and random BCs and also efficiently collapses UMIs, either just for exon mapping reads or for both exon and intron mapping reads. Another unique feature of *zUMIs* is the adaptive downsampling function, that facilitates dealing with hugely varying library sizes, but also allows to evaluate whether the library has been sequenced to saturation. *zUMIs* flexibility allows to accommodate data generated with any of the major scRNA-seq protocols that use BCs and UMIs. To illustrate the utility of *zUMIs*, we analysed a single-nucleus RNA-seq dataset and show that more than 35% of all reads map to introns. We furthermore show that these intronic reads are informative about expression levels, significantly increasing the number of detected genes and improving the cluster resolution.
Availability: https://github.com/sdparekh/zUMIs

Key words: single-cell RNA sequencing, Digital gene expression, Unique Molecular Identifiers, Pipeline

Introduction

The recent development of increasingly sensitive protocols allows to generate RNA-seq libraries of single cells [1]. The throughput of such single-cell RNA-sequencing (scRNA-seq) protocols is rapidly increasing, enabling the profiling of tens of thousands of cells [2, 3] and opening exciting possibilities to analyse cellular identities [4, 5]. As the required amplification from such low starting amounts introduces substantial amounts of noise [6], many scRNA-seq protocols incorporate unique molecular identifiers (UMIs) to label individual cDNA molecules with a random nucleotide sequence before amplification [7]. This enables the computational removal of amplification noise and thus increases the power to detect expression differences between cells [8, 9]. To increase the throughput, many protocols also incorporate sample-specific barcodes (BCs) to label all cDNA molecules of a single cell with a nucleotide sequence before library generation [10, 2]. This allows for early pooling, which further decreases amplification noise [6]. Additionally, for cell types such as neurons it has been proven to be more feasible to isolate RNA from single nuclei rather than whole cells [11, 12]. This decreases mRNA amounts further, so that it has been suggested to count intron-mapping reads originating from nascent RNAs as part of single cell expression profiles [11]. However, the few bioinformatic tools that process RNA-seq data with UMIs and BCs have limitations. For example the Drop-seq pipeline is not open source

Compiled on: October 17, 2017.
Draft manuscript prepared by the author.

Key Points

- zUMIs processes UMI-based RNA-seq data from raw reads to count tables in one command.
- Unique features of zUMIs:
  - Automatic cell barcode selection
  - Adaptive downsampling
  - Counting of intron-mapping reads for gene expression quantification
- zUMIs is compatible with all major UMI based RNA-seq library protocols.

[10]. While Cell Ranger is open, it is exceedingly difficult to adapt the code to new or unknown sample barcodes and other library types. Other tools are specifically designed to work with one mapping algorithm and focus mainly on transcriptomes [13, 14]. Furthermore, to our knowledge, no UMI-RNA-seq pipeline provides the utility to also consider intron mapping reads [2, 15, 14, 13, 16]. Here, we present *zUMIs*, a fast and flexible pipeline that overcomes these limitations.

Findings

zUMIs is a pipeline that processes paired fastq files containing the UMI and BC reads and the cDNA sequence. Read pairs are filtered to remove reads with bad BCs or UMIs based on sequence quality and the remaining reads are then mapped to the genome (Figure 1). To allow the quantification of intronic reads that are generated from unspliced mRNAs, especially when using nuclei as input material, *zUMIs* generates separate UMI and read count tables for exons, introns and exon+introns. Another unique feature of *zUMIs* is that it allows for downsampling of reads before collapsing UMIs, uniquely enabling the user to assess whether a library was sequenced to saturation or whether deeper sequencing is necessary to depict the full mRNA complexity. Furthermore, *zUMIs* is flexible with respect to the length and sequences of the BC and UMIs, supporting protocols that have both sequences in one read [17, 18, 10, 14, 3, 2, 12] or split across several reads, as is the case in the InDrops v3 [19, 20] and STRT-2i [21] methods. Thus, *zUMIs* is compatible with all major UMI-based scRNA-seq protocols. Finally, *zUMIs* can be easily installed as an application on any unix machine or be conveniently deployed for cloud computing at Amazon's elastic compute service with a provided machine image.

Implementation and Operation

Pre-processing, Mapping and Counting

The input for *zUMIs* is a group of paired fastq files, where one file contains the cDNA sequence and the other file(s) the read(s) containing the BC and UMI. The exact location and length of UMI and BC are specified by the user, thus *zUMIs* can process sequences obtained from any scRNA-seq with UMIs. The first step in our pipeline is to filter reads that have low quality BCs according to a user-defined threshold, this should eliminate the bulk of spurious BCs. A similar sequence quality based cutoff can be applied to the UMI. Others have suggested to use edit distances and frequencies of the UMIs to collapse spurious counts due to errors [16]. However, in the data that we analyzed, quality filtering of UMIs had no significant impact on the power to detect differentially expressed genes (Figure 2), implying that the computationally expensive distance filter will be mostly unnecessary.

The remaining reads are then mapped to the genome using the splice-aware aligner STAR [22]. The user is free to customize mapping by using the options of STAR. Furthermore, if the user wishes to use a different mapper, it is also possible to provide *zUMIs* with an aligned bam-file instead of the fastq-file with the cDNA sequence, with the sole requirement that only one mapping position per read is reported in the bam-file. Next, reads are assigned to genes and to exons or introns based on the provided gtf file, while ensuring introns are not overlapping with any exon. `Rsubread featureCounts` [23] is used to first assign reads to exons and afterwards to check whether the remaining reads fall into introns. The output is then read into R using `data.table` [24] count tables for UMIs and reads per gene per BC are generated. Only identical UMI sequences that were mapped either to the exon or intron of the same gene are collapsed. Note that only the processing of intron and exon reads together allows to properly collapse UMIs that can be sampled from the intronic as well as from the exonic part of the same nascent mRNA molecule.

Cell Barcode Selection

In order to be compatible with well-based and droplet-based scRNA-seq methods, *zUMIs* needs to be able to deal with known as well as random BCs. As default behavior, *zUMIs* infers which barcodes mark good cells from the data (Figure 3 A,B). To this end, we fit a k-dimensional multivariate normal distribution [25, 26] for the number of reads/BC, and reason that only the kth normal distribution with the largest mean contains barcodes that identify reads originating from intact cells. We exclude all barcodes that fall in the lower 1% tail of this distribution. The HEK dataset used in this paper contains 96 cells with known barcodes and *zUMIs* identifies 99 barcodes as intact, including all the 96 known barcodes. Also for the single-nucleus RNA-seq from Habib et al.[12] *zUMIs* identified a reasonable number of cells: Habib et al. report 10,877 nuclei and zUMIs identified 11,013 intact nuclei. However, if the number of barcodes or barcode sequences are known, it is preferable to use this information. In the case that *zUMIs* is either given the number of BCs or is provided with a list of BC sequences, it will use this information and forgo automatic inference.

Downsampling

scRNA-seq library sizes can vary by orders of magnitude, which complicates normalization [27, 28]. A straight-forward solution for this issue is to downsample over-represented libraries [29]. *zUMIs* has an inbuilt function for downsampling datasets to a user-specified number of reads or a range of reads. By default, *zUMIs* downsamples all selected barcodes to be within three absolute deviations from the median number of reads per barcode (Figure 3 C). Alternatively, the user can provide a target sequencing depth and *zUMIs* will downsample to the specified read number or omit the sample from the downsampled count table. Furthermore, *zUMIs* also allows to specify multiple target read number at once for downsampling. This feature is helpful, if the user wishes to determine whether the RNA-seq library was sequenced to saturation or whether further sequencing would increaset the number of detected genes or UMIs enough to justify the extra cost. In our HEK-cell exam-

ple dataset the number of detected genes starts leveling of at one million reads, sequencing double that amount would only increase the number of detected genes from 9,000 to 10,600, when counting exon reads (Figure 3D). The saturation curve of exon+intron reads runs parallel to the one for exon reads, both indicating that a sequencing depth of one million reads per cell is sufficient for these libraries.

Output and Statistics

zUMIs outputs three UMI and three read count tables: gene-wise counts for traditional exon mapping, one for intron and one for exon+intron counts. If a user chooses the downsampling option, 6 additional count-tables per target read count are provided. To evaluate library quality *zUMIs* summarizes the mapping statistics of the reads. While exon and intron mapping reads likely represent mRNA quantities, a high fraction of intergenic and unmapped reads indicates low-quality libraries. Another measure of RNA-seq library quality is the complexity of the library, for which the number of detected genes and the number of identified UMIs are good measures (Figure 1). We processed 227 million reads with *zUMIs* and quantified expression levels for exon and intron counts on a unix machine using up to 16 threads, which took barely 3 hours. Increasing the number of reads increases the processing time approximately linearly, where filtering, mapping and counting each take up roughly one third of the total time (Figure 3 E). We also observe that the peak RAM usage for processing datasets of 227, 500 and 1000 million pairs was 42 Gb, 89 Gb and 172 Gb, respectively. Finally, *zUMIs* could process the largest scRNA-seq dataset reported to date with around 1.3 million brain cells and 25 billion read pairs generated with 10xGenomics Chromium https://support.10xgenomics.com/single-cell-gene-expression/datasets/1.3.0/1M_neurons on a 22-core Intel Xeon E5-2699 processor in only 7 days.

Intron Counting

Assuming that intron mapping reads originate from nascent mRNAs, *zUMIs* also counts and collapses intron mapping reads with other reads mapping to the same gene with the same UMI. To assess the information gain from intronic reads to estimate gene expression levels, we analysed a publicly available DroNc-seq mouse brain dataset ([12], https://portals.broadinstitute.org/single_cell). For the $\sim 11,000$ single nuclei of this dataset, the fraction of intron mapping reads of all reads goes upto 61%. Thus, if intronic reads are considered, the mean number of detected genes per cell increases significantly from 1041 for exon reads to 1995 for exon+intron reads (Welch two sample t-test: p-value < 2.2e-16). To assess the impact of intronic reads on the inference of differential expression, we performed power simulations using empirical mean and dispersion distributions from this dataset [9]. The simulations assumed a balanced two-group comparison of variable sample sizes with 10% of the genes differentially expressed between groups. We observed a 0.5% decrease of the marginal false discovery rate (FDR) for exon+intron relative to exon counts for group sample sizes of $<$ 250 cells, while the power to detect differentially expressed genes was similar for exon and exon+intron counts. Next, we investigated whether exon+intron counting improves the identification of cell types, as suggested in [11]. Following the Seurat pipeline [30], we clustered the cells of the DroNc-seq dataset based on the exon as well as our exon+intron counts. The KNN-clustering reported 24 distinct clusters for the exon+intron counts, while we could only discriminate 15 clusters using exon counts (Figure 4). This analysis shows, that the additional genes that were detected by also counting intron-mapping reads are not spurious, but carry biological meaning.

Conclusion

zUMIs is a fast and flexible pipeline processing raw reads to obtain count tables for RNA-seq data using UMIs. To our knowledge it is the only open source pipeline that has a barcode and UMI quality filter, allows intron counting and has an integrated downsampling functionality. These features ensure that *zUMIs* is applicable to most experimental designs of RNA-seq data, including single nucleus sequencing techniques, droplet-based methods where the BC is unknown, as well as plate-based UMI-methods with known BCs. Finally, *zUMIs* is computationally efficient, user-friendly and easy to install.

Availability of Source Code and Requirements

- Project name: zUMIs
- Project home page: https://github.com/sdparekh/zUMIs
- Operating system(s): UNIX
- Programming language: shell, R, perl
- Other requirements: STAR >= 2.5.3a, R >= 3.4, pigz >= 2.3 & samtools >= 1.1
- License: GNU GPLv3.0

Availability of supporting data and materials

All data that were generated for this project were submitted to GEO under accession GSE99822.

Declarations

List of Abbreviations

scRNA-seq - single-cell RNA-sequencing
UMI - Unique Molecular Identifier
BC - Barcode
MAD - Median Absolute Deviation

Competing Interests

The author(s) declare that they have no competing interests.

Funding

This work has been supported by the DFG through SFB1243 subprojects A14/A15.

Author's Contributions

SP and CZ designed and implemented the pipeline. BV tested the pipeline and helped in power simulations. SP, CZ, WE and IH wrote the manuscript. All authors read and approved the final manuscript.

References

1. Sandberg R. Entering the era of single-cell transcriptomics in biology and medicine. Nat Methods 2014 Jan;11(1):22–24.
2. Zheng GXY, Terry JM, Belgrader P, Ryvkin P, Bent ZW, Wilson R, et al. Massively parallel digital transcriptional profiling of single cells. Nat Commun 2017 16 Jan;8:14049.

3. Rosenberg AB, Roco C, Muscat RA, Kuchina A, Mukherjee S, Chen W, et al. Scaling single cell transcriptomics through split pool barcoding. bioRxiv 2017 2 Feb;p. 105163.
4. Wagner A, Regev A, Yosef N. Revealing the vectors of cellular identity with single-cell genomics. Nat Biotechnol 2016 8 Nov;34(11):1145–1160.
5. Regev A, Teichmann S, Lander ES, Amit I, Benoist C, Birney E, et al. The Human Cell Atlas. bioRxiv 2017 8 May;p. 121202.
6. Parekh S, Ziegenhain C, Vieth B, Enard W, Hellmann I. The impact of amplification on differential expression analyses by RNA-seq. Sci Rep 2016 9 May;6:25533.
7. Kivioja T, Vähärautio A, Karlsson K, Bonke M, Enge M, Linnarsson S, et al. Counting absolute numbers of molecules using unique molecular identifiers. Nat Methods 2012 Jan;9(1):72–74.
8. Ziegenhain C, Vieth B, Parekh S, Reinius B, Guillaumet-Adkins A, Smets M, et al. Comparative Analysis of Single-Cell RNA Sequencing Methods. Mol Cell 2017 16 Feb;65(4):631–643.e4.
9. Vieth B, Ziegenhain C, Parekh S, Enard W, Hellmann I. powsimR: Power analysis for bulk and single cell RNA-seq experiments. Bioinformatics 2017 Jul;.
10. Macosko EZ, Basu A, Satija R, Nemesh J, Shekhar K, Goldman M, et al. Highly Parallel Genome-wide Expression Profiling of Individual Cells Using Nanoliter Droplets. Cell 2015 21 May;161(5):1202–1214.
11. Lake BB, Ai R, Kaeser GE, Salathia NS, Yung YC, Liu R, et al. Neuronal subtypes and diversity revealed by single-nucleus RNA sequencing of the human brain. Science 2016 24 Jun;352(6293):1586–1590.
12. Habib N, Avraham-Davidi I, Basu A, Burks T, Shekhar K, Hofree M, et al. Massively parallel single-nucleus RNA-seq with DroNc-seq. Nat Methods 2017 Oct;14(10):955–958.
13. Svensson V, Natarajan KN, Ly LH, Miragaia RJ, Labalette C, Macaulay IC, et al. Power analysis of single-cell RNA-sequencing experiments. Nat Methods 2017 6 Mar;.
14. Hashimshony T, Senderovich N, Avital G, Klochendler A, de Leeuw Y, Anavy L, et al. CEL-Seq2: sensitive highly-multiplexed single-cell RNA-Seq. Genome Biol 2016 28 Apr;17(1):77.
15. Macosko EZ, Basu A, Satija R, Nemesh J, Shekhar K, Goldman M, et al. Highly Parallel Genome-wide Expression Profiling of Individual Cells Using Nanoliter Droplets. Cell 2015 21 May;161(5):1202–1214.
16. Smith TS, Heger A, Sudbery I. UMI-tools: Modelling sequencing errors in Unique Molecular Identifiers to improve quantification accuracy. Genome Res 2017 18 Jan;.
17. Soumillon M, Cacchiarelli D, Semrau S, van Oudenaarden A, Mikkelsen TS. Characterization of directed differentiation by high-throughput single-cell RNA-Seq. bioRxiv 2014 5 Mar;.
18. Jaitin DA, Kenigsberg E, Keren-Shaul H, Elefant N, Paul F, Zaretsky I, et al. Massively parallel single-cell RNA-seq for marker-free decomposition of tissues into cell types. Science 2014 14 Feb;343(6172):776–779.
19. Klein AM, Mazutis L, Akartuna I, Tallapragada N, Veres A, Li V, et al. Droplet barcoding for single-cell transcriptomics applied to embryonic stem cells. Cell 2015 21 May;161(5):1187–1201.
20. Zilionis R, Nainys J, Veres A, Savova V, Zemmour D, Klein AM, et al. Single-cell barcoding and sequencing using droplet microfluidics. Nat Protoc 2017 Jan;12(1):44–73.
21. Hochgerner H, Lännerberg P, Hodge R, Mikes J, Heskol A, Hubschle H, et al. STRT-seq-2i: dual-index 5' single cell and nucleus RNA-seq on an addressable microwell array. bioRxiv 2017 20 Apr;p. 126268.
22. Dobin A, Davis CA, Schlesinger F, Drenkow J, Zaleski C, Jha S, et al. STAR: ultrafast universal RNA-seq aligner. Bioinformatics 2013 1 Jan;29(1):15–21.
23. Liao Y, Smyth GK, Shi W. featureCounts: an efficient general purpose program for assigning sequence reads to genomic features. Bioinformatics 2014 1 Apr;30(7):923–930.
24. Dowle M, Srinivasan A. data.table: Extension of 'data.frame'; 2017, https://CRAN.R-project.org/package=data.table, r package version 1.10.4.
25. Fraley C, Raftery AE. Model-Based Clustering, Discriminant Analysis, and Density Estimation. J Am Stat Assoc 2002 Jun;97(458):611–631.
26. Fraley C, Raftery AE, Brendan Murphy T, Scrucca L. mclust Version 4 for R: Normal Mixture Modeling for Model-Based Clustering, Classification, and Density Estimation 2012;.
27. Vallejos CA, Risso D, Scialdone A, Dudoit S, Marioni JC. Normalizing single-cell RNA sequencing data: challenges and opportunities. Nat Methods 2017 Jun;14(6):565–571.
28. Evans C, Hardin J, Stoebel DM. Selecting between-sample RNA-Seq normalization methods from the perspective of their assumptions. Brief Bioinform 2017 27 Feb;.
29. Grün D, van Oudenaarden A. Design and Analysis of Single-Cell Sequencing Experiments. Cell 2015 5 Nov;163(4):799–810.
30. Butler A, Satija R. Integrated analysis of single cell transcriptomic data across conditions, technologies, and species. bioRxiv 2017 Jul;p. 164889.

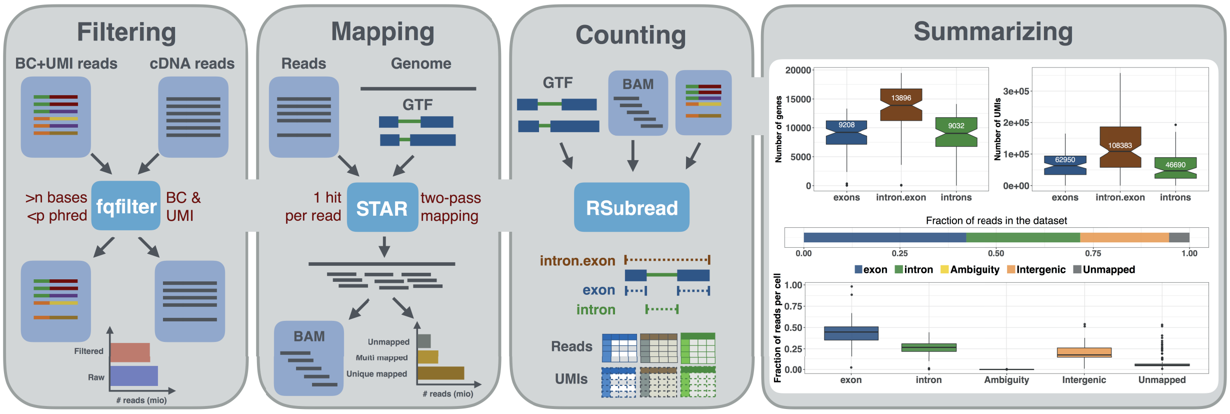

Figure 1. Schematic of the zUMIs pipeline. Each of the grey panels from left to right depicts a step of the *zUMIs* pipeline. First, fastq files are filtered according to user-defined barcode (BC) and unique molecular identifier (UMI) quality thresholds. Next, the remaining cDNA reads are mapped to the reference genome using STAR. Gene-wise read and UMI count tables are generated for exon, intron and exon+intron overlapping reads. To obtain comparable library sizes, reads can be downsampled to a desired range during the counting step. In addition, *zUMIs* also generates data and plots for several quality measures, such as the number of detected genes/UMIs per barcode and distribution of reads into mapping feature categories.

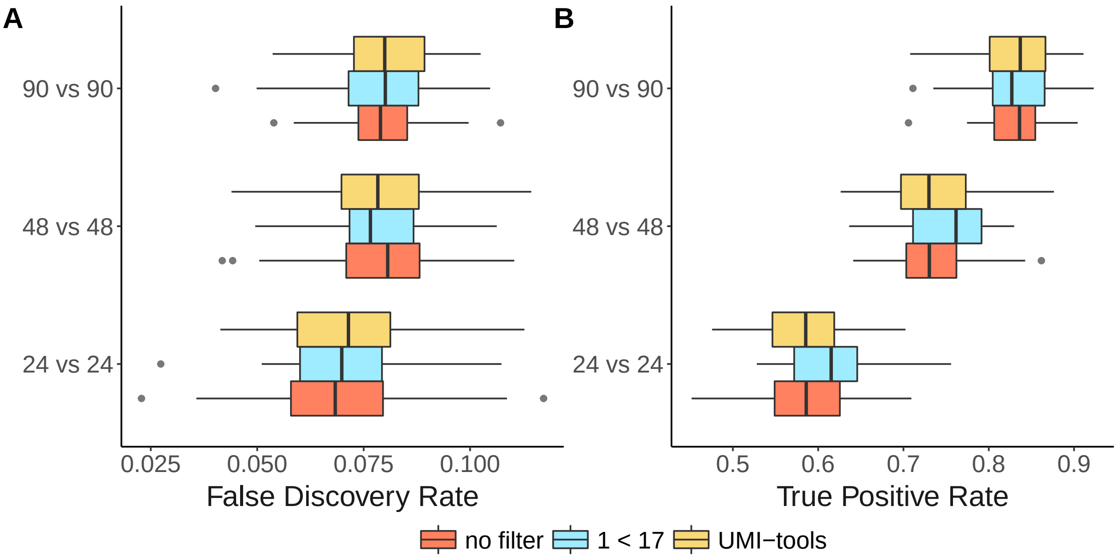

Figure 2. Impact of UMI quality filtering on Differential Gene Expression. We estimated the mean expression and dispersion of genes across the cells from our HEK dataset without any UMI quality filters (red); reads where the UMI has at least one base with a quality score < 17 (blue) and using the directional-adjacency method implemented in UMI-tools[16] (yellow), that collapses UMIs based on their distance in a sequence graph also considering the frequency. The resulting count matrices were then used for power simulations using powsimR [9] with balanced sample sizes of n in each group. We performed 50 simulations with 9000 genes where 10% of the genes are differentially expressed with log2 fold changes drawn from a normal distribution $N(\mu = 0, \sigma = 1.5)$. We report here A) false discovery rate (FDR) and B) true positive rate (TPR) to detect differential expression for each filtering criterion.

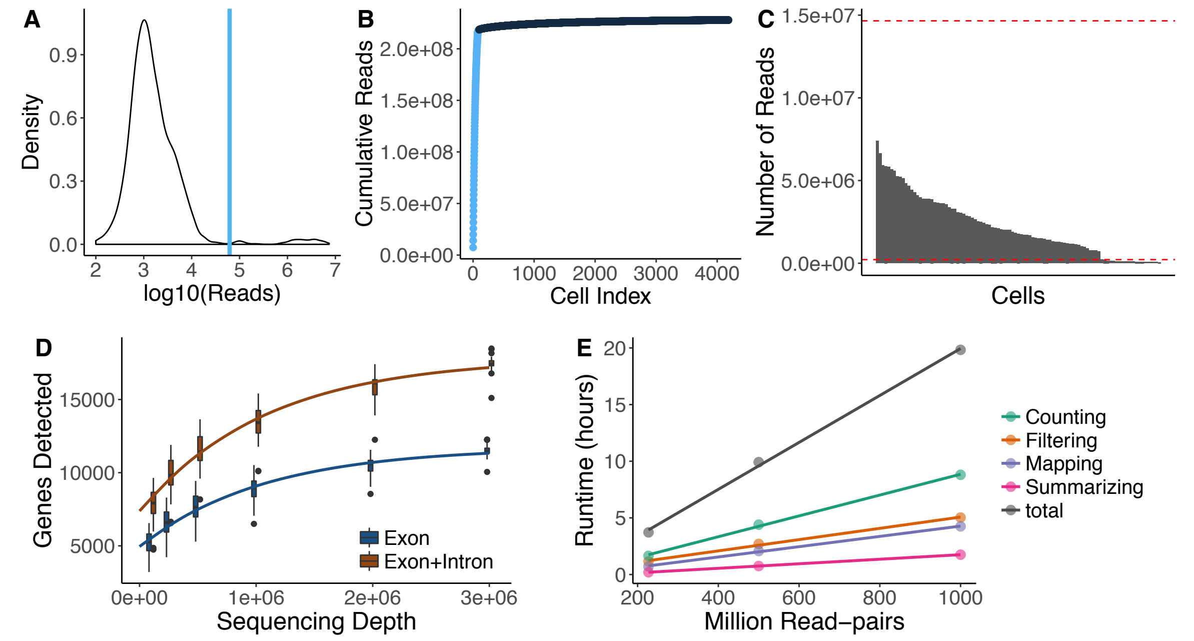

Figure 3. Utilities of zUMIs. Each of the panels shows the utilities of *zUMIs* pipeline. The plots from A-D are the results from the example HEK dataset used in the paper. A) The plot shows a density distribution of reads per barcode. Cell barcodes with reads above the blue line are selected. B) The plot shows the cumulative read distribution in the example HEK dataset where the barcodes in light blue are the selected cells. C) The barplot shows the number of reads per selected cell barcode with the red lines showing upper and lower MAD (Median Absolute Deviations) cutoffs for adaptive downsampling. Here, the cells below the lower MAD have very low coverage and are discarded in downsampled count tables. D) Cells were downsampled to six depths from 100,000 to 3,000,000 reads. For each sequencing depth the genes detected per cell is shown. E) Runtime for three datasets with 227, 500 and 1000 million read-pairs. The runtime is divided in the main steps of the *zUMIs* pipeline: Filtering, Mapping, Counting and Summarizing. Each dataset was processed using 16 threads ("-p 16").

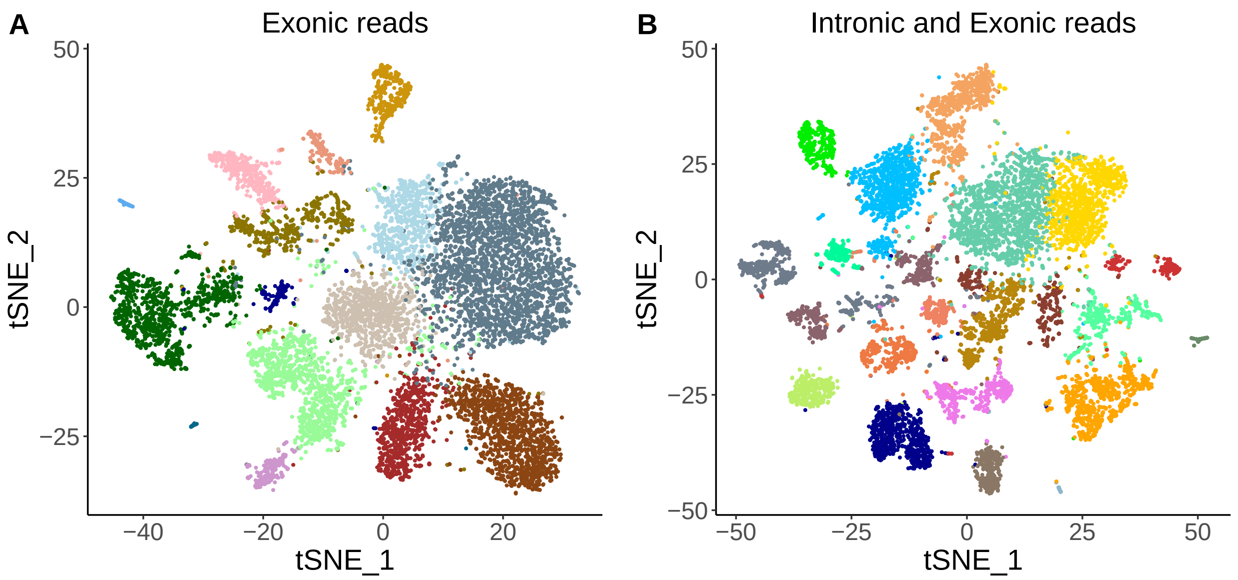

Figure 4. Contribution of intron reads in scRNA-seq. We analyse published single-nucleus RNA-seq data[12] to assess the utility of counting intron reads. We processed the raw data with *zUMIs* to obtain a count table with exon reads as well as exon+intron reads. We follow the Seurat pipeline[30] for filtering, normalising and clustering of cells for exon and exon+intron count tables and find 15 and 24 clusters, respectively. The t-SNE plot in panel (A) is colored by cluster identity of exon reads and panel (B) colored by cluster identity from exon+intron reads.

Reference PDF

Click here to access/download
Supplementary Material
zumis_fast_flexible (1).pdf

Personal Cover Click here to download Personal Cover CoverLetterGiga.pdf

LUDWIG-MAXIMILIANS-UNIVERSITÄT MÜNCHEN

BIOZENTRUM
DEPARTMENT BIOLOGIE II

Dr. Ines Hellmann
Department of Biology
Anthropology and Human genetics
Großhadernerstr. 2
D-82152 Planegg-Martinsried, Germany

Phone +49 (0)89 / 2180 - 74336
Fax +49 (0)89 / 2180 – 74331
mail: hellmann@bio.lmu.de

17. Oktober 2017

Submission of Parekh et al.: “zUMIs: A fast and flexible pipeline to process RNA sequencing data with UMIs”

Dear Editor,

We would like to submit our paper entitled “zUMIs: A fast and flexible pipeline to process RNA sequencing data with UMIs” as Technical Note to GigaScience. RNA-seq methods have become more and more sensitive, so that today RNA-seq libraries are prepared from single cells or even single nuclei. This increase in sensitivity is only possible if the cDNA is amplified, and thus comes at the cost of increased noise. This problem is commonly solved by adding unique molecular identifiers (UMIs) to tag single mRNA molecules. However, current pipelines that make use of UMIs to remove amplification noise appear to be solely written for one method and hence lack the flexibility to be used for other library designs and are often not open source.

Our new pipeline zUMIs solves this problem. The user can flexibly define the locations of the UMI and the cell barcode in the sequencing layout. zUMIs provides a heuristic to identify good cell barcodes, which is necessary if the cell barcodes are random as it is the case for droplet based methods. Furthermore, due to the extremely sparse starting material for single nucleus sequencing it is recommended to also count intron mapping reads and to our knowledge zUMIs is the only UMI-counting pipeline that offers this functionality, thus improving single cell clustering. These features make zUMIs highly useful for all prominent RNA-seq methods that use UMIs.

Finally, zUMIs also helps to make the often hugely varying library sizes of single cell data comparable by implementing a downsampling function for cells with excessive number of reads. All results are neatly summarized in tab-delimited text and R-files and we also provide plots and tables for quality assessment of the libraries. Furthermore, zUMIs is easily deployed in the cloud with our provided Amazon Machine Image (AMI).
In summary, we believe that there is great need of a tool such as zUMIs in the RNA-seq community.
We hope that you also find our manuscript interesting and are willing to consider it for publication in GigaScience , which we feel would be the best place.

With best regards,

Dr Ines Hellmann
