# Supplementary material for: zUMIs - A fast and flexible pipeline to process RNA sequencing data with UMIs
Source: Gigascience. 2018 May 26;7(6):giy059. doi: 10.1093/gigascience/giy059 (PMC6007394; doi:10.1093/gigascience/giy059)
Supplement: GIGA-D-17-00271_Revision_2.pdf [file giy059_giga-d-17-00271_revision_2.pdf]

GigaScience

zUMIs - A fast and flexible pipeline to process RNA sequencing data with UMIs

--Manuscript Draft--

**Manuscript Number:**	GIGA-D-17-00271R2
**Full Title:**	zUMIs - A fast and flexible pipeline to process RNA sequencing data with UMIs
**Article Type:**	Technical Note
**Funding Information:**	Deutsche Forschungsgemeinschaft (SFB1243 - A15) — Dr. Ines Hellmann<br>Deutsche Forschungsgemeinschaft (SFB1243 - A14) — Prof. Wolfgang Enard
**Abstract:**	Single cell RNA-seq (scRNA-seq) experiments typically analyze hundreds or thousands of cells after amplification of the cDNA. The high throughput is made possible by the early introduction of sample-specific barcodes (BCs) and the amplification bias is alleviated by unique molecular identifiers (UMIs). Thus the ideal analysis pipeline for scRNA-seq data needs to efficiently tabulate reads according to both BC and UMI.<br>zUMIs is such a pipeline, it can handle both known and random BCs and also efficiently collapses UMIs, either just for exon mapping reads or for both exon and intron mapping reads. Another unique feature of zUMIs is the adaptive downsampling function, that facilitates dealing with hugely varying library sizes, but also allows to evaluate whether the library has been sequenced to saturation. zUMIs flexibility allows to accommodate data generated with any of the major scRNA-seq protocols that use BCs and UMIs. To illustrate the utility of zUMIs, we analysed a single-nucleus RNA-seq dataset and show that more than 35% of all reads map to introns. We furthermore show that these intronic reads are informative about expression levels, significantly increasing the number of detected genes and improving the cluster resolution.
**Corresponding Author:**	Ines Hellmann<br>Ludwig-Maximilians-Universitat Munchen Fakultat fur Biologie<br>Martinsried, GERMANY
**Corresponding Author Secondary Information:**	
**Corresponding Author's Institution:**	Ludwig-Maximilians-Universitat Munchen Fakultat fur Biologie
**Corresponding Author's Secondary Institution:**	
**First Author:**	Swati Parekh
**First Author Secondary Information:**	
**Order of Authors:**	Swati Parekh<br>Christoph Ziegenhain<br>Beate Vieth<br>Wolfgang Enard<br>Ines Hellmann
**Order of Authors Secondary Information:**	
**Response to Reviewers:**	Note: For better readability, we only include the reviewer reports with remaining concerns in the point by point response below.<br><br>Reviewer reports:<br>Reviewer #2: Review of Revised version R1 of "zUMIs - A fast and flexible pipeline to process RNA sequencing data with UMIs"<br><br>Parekh et al. have partially addressed my concerns, however there remain unresolved issues that have critical importance to the conclusions of the manuscript. In particular,

Powered by Editorial Manager® and ProduXion Manager® from Aries Systems Corporation

although the authors have included a Methods section with the revision, some methodological details for reproducibility are still missing. In addition, although the authors have compared one aspect of the pipeline to existing tools, other steps of the pipeline that are also carried out by other tools are not compared. Finally, the biological relevance of the differences in clustering with and without introns is still not convincingly demonstrated. These remaining concerns are detailed below (new responses marked by ***).

AUTHOR RESPONSE:
We want to clarify that we do not wish to claim that counting introns is a good idea in general. However, we argue that for extremely sparse datasets such as generated by single nuclei sequencing, having Intron counts is better than losing even more genes. We hope that we could make this clearer in the text, as such:

"Furthermore, we think that although noisy, the large number of additionally detected genes makes Exon+Intron counting worthwhile for extremely sparse data."

***
REVIEWER RESPONSE:
Thank you for the clarification. However, this recommendation remains vague. It would be useful to define what is meant by "extremely sparse data". It may not be clear to the single-cell community, since all single-cell data is quite sparse by nature. But it seems this recommendation is focused on a particular subset of protocols (DroNc-seq)? Please clarify.
***

AUTHOR RESPONSE 2:
We have revised this text passage to be more specific, so readers know when to consider Exon+Intron counting. Our recommendation is now differentiated between nucleus sequencing that is enriched in intronic sequences because of nascent mRNA molecules and low coverage sparse data generated in high throughput applications.

“Furthermore, we think that, although potentially noisy, the large number of additionally detected genes makes Exon+Intron counting worthwhile, especially for single- nuclei sequencing techniques that are enriched for nuclear nascent RNA transcripts, such as DroNc-seq [12]. Additionally, Exon+Intron counting may help extracting as much information as possible from low coverage data as generated in the context of high-throughput cell atlas efforts (eg 10,000- 20,000 reads/cell [37, 38]. Lastly, users should always exclude the possibility of intronic reads stemming from genomic DNA contamination in the library preparation by confirming low intergenic mapping fractions using the statistics output provided by zUMIs.”

I have identified several issues that the authors should address in order to improve the manuscript, which are detailed below and divided into major (of critical importance) and minor (to improve clarity) categories.

Major Comments:
1. Methodological details are missing throughout. The method should be described more completely and clearly, and any analyses or comparisons should be made reproducible. For example:
* What differential expression method was used in the simulation study to compare UMItools and zUMI?
* What options were used with powsimR in the simulation study?
* How is the k-dimensional multivariate normal distribution fit in the cell barcode selection step?
* How is k determined in the cell barcode selection step?
* How was data simulated for the Intron evaluation?
* What options were used in applying the Seurat pipeline to cluster cells?

AUTHOR RESPONSE:
We added a methods section (Page:3-4) that includes subsections for (1) data generation of the HEK dataset as well as data processing of other used datasets, (2) the powsimR simulations and (3) the use of the Seurat pipeline. The passage about the Cell-Barcode selection was changed in the main text (Page:2). We hope to have made our barcode selection clearer in the main text.

"To this end, we fit a k-dimensional multivariate normal distribution using the R-package mclust [25, 26] for the number of reads/BC, where k is empirically determined by mclust via the Bayesian Information Criterion (BIC). We reason that only the kth normal distribution with the largest mean contains barcodes that identify reads originating from intact cells."

***
REVIEWER RESPONSE:
The authors added a Methods section. Most of the details seem to have been added, but there are still some details that I have questions about. For example, how was the Intron-sampling experiment carried out (Figure 1 in Additional File 1)?
What is meant by "sufficiently many cells for DE analysis" (page 3)?
***

AUTHOR RESPONSE 2:
We now added a description of the Intron-Sampling to Clustering part of the Methods section.
Sorry, for the confusion about the statement “sufficiently many cells …” - In this analysis we included all genes that were detected, i.e. had at least one read mapped. We changed this sentence.
“For a fair comparison, we include all detected genes”.
---

2. One major conclusion of the paper is that incorporation of intron-mapping reads significantly improves cluster resolution. It is perhaps not surprising that including the Intron counts results in a higher mean number of genes detected, but the authors conclude that since more clusters are also found that this means the additional reads are biologically meaningful. Unfortunately, the authors have not provided any evidence that this is the case. The fact that more clusters are seen says nothing about the difference between technical and biological sources of variation. If these additional clusters also corresponded to some independently measured biological covariate, the argument would have basis.

AUTHOR RESPONSE:
While we do not wish to claim that counting of intron-mapping reads is recommended in all cases of scRNA-seq, we do think it is valid and helpful for extremely sparse datasets such as the DroNc-seq data from Habib et al. (2017). We now provide detailed analyses of differences between newly formed subclusters using Exon+Intron counting. We find not only more genes, but also more significantly differentially expressed genes between subclusters when using Exon+Intron UMI data (Figure 4D). Furthermore, log2 fold changes (LFC) for the groups that were split up more when using Exon+Intron counting corresponded well to the Exon-only LFC (see the new Figure 4F). Additionally, we illustrate the biological relevance of subclusters found with Exon+Intron data by the example of the transcriptomic subtypes of GABAergic Pvalb-type Neurons marked by Il1rapl2 expression. We have added this evidence to the 'Intron Counting' section and included methodological details in the appropriate Methods sections.
Lastly, we have excluded the possibility of Intron-mapping reads being spurious by sampling fake intronic reads and attempting cluster identification (see response to Reviewer 1, point 3).

***
REVIEWER RESPONSE:

Powered by Editorial Manager® and ProduXion Manager® from Aries Systems Corporation

The authors have not fully addressed the concern of biologically meaningful clustering results. They highlight the example of a single gene, which is not convincing that the results are systematically meaningful. In main text they state that 5% of the additional genes found are marker genes, but no baseline is given to be able to judge if that is a significant result. What percentage of genes overall are marker genes? What percentage of DE genes by exon only are marker genes?

Furthermore while they have shown that there are more DE genes between sub-clusters with introns included (again this is not surprising since more genes are detected), this result could be influenced to a degree by the use of a model (limma-trend) that is not appropriate for sparse RNA-seq data (see also response to Major comment 6).

In addition, it seems that the figures or additional results presented in the Additional File (including the "sampling fake intronic reads") are never mentioned in the manuscript. To me, the sampling fake intronic reads analysis could be a valuable addition to supporting the conclusion that utilizing the intronic reads gives biologically meaningful clustering results (as long as the details of this simulation are realistic, but these details are currently not provided).

As an additional note, it is not clear what color represents in newly added Figures 4D, G, and H and this is not defined in the legend. It is also not clear what color represents in Figure 4A and B - some colors seem to map to the legend of 4C, but not all of them.
***

AUTHOR RESPONSE 2:
We extended the section where we cite a number of papers that utilize intron counts in the analysis of single cell RNA-seq and in particular single nuclei sequencing data. What information can be gained from intron counting is indeed a hot topic in the single cell community and even though we believe to contribute some evidence to support the notion that intron counts add biologically meaningful information, our paper is hardly the place where this issue can be fully resolved. However, the intron counting utility of zUMIs will facilitate research in this area and thus ultimately help other researchers to address this question.

Concerning the additionally detected marker genes: We do not expect any enrichment of marker genes when we use Exon+Intron counting and the 5% additional markers are roughly the level expected: 4 % of all detected Exon+Intron genes are also marker genes. However, detecting more of them gives us a better chance to later classify the cells.
We also agree that it is not surprising that we find more DE genes if we detect more genes overall. Including introns simply allows us to better detect present transcripts. On the contrary, we would be reluctant to recommend the inclusion of Introns if they would lead to a major shift in the expression profiles, i.e. many more DE- or marker genes than expected.

We now added the fake intron analysis to Figure 4 and also extended the methods section for Cluster Identification to describe our sampling:

“To illustrate that the additional clusters found by counting Exon+Intron reads are not spurious, we use Intron-only UMI-counts from the same data to add to the observed Exon only counts. More specifically, to each gene we add scran-sizeFactor corrected Intron counts from the same gene after permuting them across cells. We assessed the cluster numbers from 100 such permutations.”

We now explain the color schemes of Figure 4 A,B &E in the figure legend and added a color legend for D,H &I (formerly D,G & H).
“Different shades of those clusters indicate that multiple clusters had the same major cell-type assigned.”
---

4. Several open-source tools exist that perform many of the steps in the zUMI pipeline

[1, 2, 3]. It would be nice to see how these perform in comparison to zUMI.

AUTHOR RESPONSE:
While several tools exist that can perform some of the steps of the zUMIs pipeline, none of them provides a comprehensive combination as zUMIs. We have added a Table to compare available features of six other pipelines geared towards scRNA-seq data with UMIs. The tool "UMI-Reducer" with reference [2] suggested by the reviewer was omitted because it seemed like a tool geared towards one specific application outside of single-cell RNA-seq. Furthermore, "UMI-Reducer" only de-duplicates UMIs with the same mapping position, which would be inappropriate for scRNA-seq protocols that fragment after preamplification, such as SCRB-seq.
Furthermore, we added a comparison of the count-tables produced by zUMIs, Drop-seq-tools and UMI-tools and generally find very good correspondence (see response to Reviewer 1).

***
REVIEWER RESPONSE
The authors have included a comparison to two other tools for the UMI-collapsing strategy. However, these other tools are not included in any other aspect. For example, how do other methods perform in cell barcode selection (Page 2)? How do other methods perform in running time (Figure 3E)?
***

AUTHOR RESPONSE 2:
The purpose of Figure 2 was never a comprehensive comparison of all UMI-collapsing tools, but it should merely provide perspective on the possible choices of UMI collapsing within zUMIs so that the user can make an informed choice given the run time and the added information.

A systematic comparison of runtimes among pipelines is beyond the scope of this Technical Note. More specifically, the input data vary widely and we would be hard pressed to find data-sets that can be fed into each of the pipelines, thus making it virtually impossible to provide a fair overall run-time comparison.

In fact zUMIs is the only tool that can handle data from all major UMI-based scRNA-seq library protocols (Table 1). Besides, we found that no other pipeline had the combination of processing featured that we found to be useful. This said, the main advantage of zUMIs is its unique combination of features and not the performance details of the separate steps.
That’s why we feel that the comparison of features of 7 UMI pipelines that we provide in Table 1 is an adequate ‘performance’ evaluation. Table 1 now also includes a column detailing the various Barcode selection options.
---

6. It is not clear how the simulation parameters in the comparison to UMI-tools directly relate to the UMI quantification. Specifically, estimating the mean and dispersion of the processed data and then using these as the basis for a simulated dataset seems pretty far removed from the observed UMI counts. The authors should also investigate differences in differential expression analysis of the actual data (not simulated data). They could also generate a simulated null comparison by randomly permuting sample labels. The same comments hold for the second simulation (evaluating Intron count inclusion).

AUTHOR RESPONSE:
We removed the simulations from the description of UMI-collapsing methods and focus our reporting on the descriptive statistics suggested by the reviewer (Figure 2 & section "Transcript counting").

***

Powered by Editorial Manager® and ProduXion Manager® from Aries Systems Corporation

REVIEWER RESPONSE
The authors have removed one of the simulations from the manuscript, but the second simulation remains. Unfortunately, I still have concerns regarding the intron count inclusion simulation. In particular, now that the authors have added methodological details, they have used a differential expression method that was developed for bulk RNA-seq and is not appropriate for sparse single-cell RNA-seq data. It could be that the differential expression results are due to a poorly fitting model with more sparse exon-only data. The authors should use a method appropriate for sparse single-cell RNA-seq data, such as MAST (Finak et al. 2015, Genome Biology) or zingeR (Van den Berge et al. 2018, Genome Biology).
***

--- AUTHOR RESPONSE 2:
It is correct that limma-trend is a method developed for bulk RNA-seq. However, if applied correctly, bulk methods are also suitable for single cell data (Dal Molin, Baruzzo, and Di Camillo 2017). A recent, thorough comparison by Soneson & Robinson (Soneson and Robinson 2018) has shown that limma-trend is one of the best available methods for single-cell differential expression analysis. It outperformed many specialised single cell methods. Most notably, limma-trend was one of the most consistent DE tools across a wide range of data sets (see figure 5 of Soneson & Robinson). TPR and FDR were comparable to MAST (see figure 4), while limma-trend has a vastly superior runtime.
Furthermore, many of the problems due to the sparsity of single cell data are introduced during normalization and we applied a normalisation method specifically developed for sparse gene expression data (scran) (Vallejos et al. 2017).

References:
Dal Molin, Alessandra, Giacomo Baruzzo, and Barbara Di Camillo. 2017. “Single-Cell RNA-Sequencing: Assessment of Differential Expression Analysis Methods.” Frontiers in Genetics 8 (May): 62. https://doi.org/10.3389/fgene.2017.00062.
Soneson, Charlotte, and Mark D. Robinson. 2018. “Bias, Robustness and Scalability in Single-Cell Differential Expression Analysis.” Nature Methods 15 (4): 255–61. https://doi.org/10.1038/nmeth.4612.
Vallejos, Catalina A., Davide Risso, Antonio Scialdone, Sandrine Dudoit, and John C. Marioni. 2017. “Normalizing Single-Cell RNA Sequencing Data: Challenges and Opportunities.” Nature Methods, May. https://doi.org/10.1038/nmeth.4292.
---

3. In the cell barcode selection step, the authors state that they remove "all barcodes that fall in the lower 1% tail of this distribution." What is the justification for this? What does this correspond to in practice? This threshold should also be denoted in Figure 3A.

AUTHOR RESPONSE:
The blue line in figure 3A corresponds to the calculated read cut-off. The normal distribution identified by mclust with the highest mean number of reads contains actual cell barcodes. Thus, setting the read cut-off to the lower 1% of this distribution is an empirical value that gives good correspondence to the known cell-barcodes for the HEK dataset (cut-off value: 52634 reads/barcode) and gave similarly good results for the DroNc-seq data analysed here. Still, in practice we recommend to always look at the elbow-plots output by zUMIs (Figure 3B). This will show whether our empirical cut-off was also valid for the dataset at hand.

***
REVIEWER RESPONSE
It would be useful to state your recommendation in the manuscript "to always look at the elbow-plots output by zUMIs (Figure 3B)" when setting the cut-off so that readers can benefit from this advice.
***

AUTHOR RESPONSE 2:

	We have added this in the main text (page 2 section: Cell Barcode Selection).<br>---
**Additional Information:**	
**Question**	**Response**
Are you submitting this manuscript to a special series or article collection?	No
**Experimental design and statistics**<br><br>Full details of the experimental design and statistical methods used should be given in the Methods section, as detailed in our Minimum Standards Reporting Checklist. Information essential to interpreting the data presented should be made available in the figure legends.<br><br>Have you included all the information requested in your manuscript?	Yes
**Resources**<br><br>A description of all resources used, including antibodies, cell lines, animals and software tools, with enough information to allow them to be uniquely identified, should be included in the Methods section. Authors are strongly encouraged to cite Research Resource Identifiers (RRIDs) for antibodies, model organisms and tools, where possible.<br><br>Have you included the information requested as detailed in our Minimum Standards Reporting Checklist?	Yes
**Availability of data and materials**<br><br>All datasets and code on which the conclusions of the paper rely must be either included in your submission or deposited in publicly available repositories (where available and ethically appropriate), referencing such data using a unique identifier in the references and in the "Availability of Data and Materials" section of your manuscript.<br><br>Have you have met the above requirement as detailed in our Minimum Standards Reporting Checklist?	Yes

Powered by Editorial Manager® and ProduXion Manager® from Aries Systems Corporation

Manuscript
Click here to download Manuscript zumis_fast_flexible_R2_ms.pdf

GigaScience, 2017, 1–9

doi: 10.1101/153940
Technical Note

TECHNICAL NOTE

zUMIs – A fast and flexible pipeline to process RNA sequencing data with UMIs

Swati Parekh[1,*,†], Christoph Ziegenhain[1,†], Beate Vieth[1], Wolfgang Enard[1] and Ines Hellmann[1,*]

[1]Anthropology & Human Genomics, Department of Biology II, Ludwig-Maximilians University, 82152 Martinsried, Germany

[*]parekh@bio.lmu.de; hellmann@bio.lmu.de
[†]Contributed equally.

Abstract

Single cell RNA-seq (scRNA-seq) experiments typically analyze hundreds or thousands of cells after amplification of the cDNA. The high throughput is made possible by the early introduction of sample-specific barcodes (BCs) and the amplification bias is alleviated by unique molecular identifiers (UMIs). Thus the ideal analysis pipeline for scRNA-seq data needs to efficiently tabulate reads according to both BC and UMI. *zUMIs* is such a pipeline, it can handle both known and random BCs and also efficiently collapses UMIs, either just for Exon mapping reads or for both Exon and Intron mapping reads. Another unique feature of *zUMIs* is the adaptive downsampling function, that facilitates dealing with hugely varying library sizes, but also allows to evaluate whether the library has been sequenced to saturation. *zUMIs* flexibility allows to accommodate data generated with any of the major scRNA-seq protocols that use BCs and UMIs. To illustrate the utility of *zUMIs*, we analysed a single-nucleus RNA-seq dataset and show that more than 35% of all reads map to Introns. We furthermore show that these intronic reads are informative about expression levels, significantly increasing the number of detected genes and improving the cluster resolution. **Availability:** https://github.com/sdparekh/zUMIs

Key words: Single-Cell RNA-Sequencing, Digital Gene Expression, Unique Molecular Identifiers, Pipeline

Introduction

The recent development of increasingly sensitive protocols allows to generate RNA-seq libraries of single cells [1]. The throughput of such single-cell RNA-sequencing (scRNA-seq) protocols is rapidly increasing, enabling the profiling of tens of thousands of cells [2, 3] and opening exciting possibilities to analyse cellular identities [4, 5]. As the required amplification from such low starting amounts introduces substantial amounts of noise [6], many scRNA-seq protocols incorporate unique molecular identifiers (UMIs) to label individual cDNA molecules with a random nucleotide sequence before amplification [7]. This enables the computational removal of amplification noise and thus increases the power to detect expression differences between cells [8, 9]. To increase the throughput, many protocols also incorporate sample-specific barcodes (BCs) to label all cDNA molecules of a single cell with a nucleotide sequence before library generation [10]. This allows for early pooling, which further decreases amplification noise [6]. Additionally, for cell types such as primary neurons it has been proven to be more feasible to isolate RNA from single nuclei rather than whole cells [11, 12]. This decreases mRNA amounts further, so that it has been suggested to count Intron mapping reads originating from nascent RNAs as part of single cell expression profiles [11]. However, the few bioinformatic tools that process RNA-seq data with UMIs and BCs have limitations (Table 1). For example the Drop-seq-tools is not open source [13]. While Cell Ranger is open, it is exceedingly difficult to adapt the code to new or unknown sample barcodes and other library types. Other tools are specifically designed to work with one mapping algorithm and focus mainly on tran-

Compiled on: April 7, 2018.
Draft manuscript prepared by the author.

Key Points

- zUMIs processes UMI-based RNA-seq data from raw reads to count tables in one command.
- Unique features of zUMIs:
  - Automatic cell barcode selection
  - Adaptive downsampling
  - Counting of Intron mapping reads for gene expression quantification
- zUMIs is compatible with all major UMI-based RNA-seq library protocols.

scriptomes [14, 15]. Furthermore, the only other UMI-RNA-seq pipeline providing the utility to also consider Intron mapping reads, dropEst [16], is only applicable to droplet-based protocols. Here, we present *zUMIs*, a fast and flexible pipeline that overcomes these limitations.

Findings

zUMIs is a pipeline to process RNA-seq data that were multiplexed using cell barcodes and also contain UMIs. Read pairs are filtered to remove reads with low quality BCs or UMIs based on sequence and then mapped to a reference genome (Figure 1). Next, *zUMIs* generates UMI and read count tables for Exon and Exon+Intron counting. We reason that especially very low input material such as from single nuclei sequencing might profit from including reads that potentially originate from nascent RNAs. Another unique feature of *zUMIs* is that it allows for downsampling of reads before collapsing UMIs, thus enabling the user to assess whether a library was sequenced to saturation or whether deeper sequencing is necessary to depict the full mRNA complexity. Furthermore, *zUMIs* is flexible with respect to the length and sequences of the BCs and UMIs, supporting protocols that have both sequences in one read [17, 18, 13, 15, 3, 2, 12] as well as protocols that provide UMI and BC in separate reads [19, 20, 21]. This makes *zUMIs* the only tool that is easily compatible with all major UMI-based scRNA-seq protocols.

Implementation and Operation

Filtering and Mapping

The first step in our pipeline is to filter reads that have low quality BCs according to a user-defined threshold (Figure 1). This step eliminates the majority of spurious BCs and thus greatly reduces the number of BCs that need to be considered for counting. Similarly, we also filter low quality UMIs.

The remaining reads are then mapped to the genome using the splice-aware aligner STAR [22]. The user is free to customize mapping by using the options of STAR. Furthermore, if the user wishes to use a different mapper, it is also possible to provide *zUMIs* with an aligned bam file instead of the fastq file with the cDNA sequence, with the sole requirement that only one mapping position per read is reported in the bam file.

Transcript counting

Next, reads are assigned to genes. In order to distinguish Exon and Intron counts, we generate two mutually exclusive annotation files from the provided gtf, one detailing Exon positions, the other Introns. Based on those annotations `Rsubread featureCounts` [23] is used to first assign reads to Exons and afterwards to check whether the remaining reads fall into Introns, in other words if a read is overlapping with intronic and exonic sequences, it will be assigned to the Exon only. The output is then read into R using `data.table` [24], generating count tables for UMIs and reads per gene per BC. We then collapse UMIs that were mapped either to the Exon or Intron of the same gene. Note that only the processing of Intron and Exon reads together allows to properly collapse UMIs that can be sampled from the intronic as well as from the exonic part of the same nascent mRNA molecule.

Per default, we only collapse UMIs by sequence identity. If there is a risk that a large proportion of UMIs remains under-collapsed due to sequence errors, *zUMIs* provides the option to collapse UMIs within a given Hamming distance. We compare the two *zUMIs* UMI-collapsing options to the recommended directional adjacency approach implemented in UMI-tools [25], using our in-house example dataset (see Methods). *zUMIs* identity collapsing yields nearly identical UMI counts per cell as UMI-tools, while Hamming distance yields increasingly fewer UMIs/cell with increasing sequencing depth (Figure 2C). Smith et al. [25] suggest that edit distance collapsing without considering the relative frequencies of UMIs might indeed overreach and over-collapse the UMIs. We suspect that this is indeed what happens in our example data, where we find that gene-wise dispersion estimates appear suspiciously truncated as expected if several counts are unduly reduce to one, the minimal number after collapsing (Figure 2D).

However, note that the above described differences are minor. By and large, there is good agreement between UMI counts obtained by UMI-tools [25], the Drop-seq pipeline [13] and *zUMIs*. The correlation between gene-wise counts of the same cell is > 0.99 for all comparisons (Figure 2B). In light of this, we would consider the > 3 times higher processing speed of *zUMIs* a decisive advantage (Figure 2A).

Cell Barcode Selection

In order to be compatible with well-based and droplet-based scRNA-seq methods, *zUMIs* needs to be able to deal with known as well as random BCs. As default behavior, *zUMIs* infers which barcodes mark good cells from the data (Figure 3 A,B). To this end, we fit a k-dimensional multivariate normal distribution using the R-package mclust [26, 27] for the number of reads/BC, where k is empirically determined by mclust via the Bayesian Information Criterion (BIC). We reason that only the kth normal distribution with the largest mean contains barcodes that identify reads originating from intact cells. We exclude all barcodes that fall in the lower 1% tail of this kth normal-distribution to exclude spurious barcodes. The HEK dataset used in this paper contains 96 cells with known barcodes and *zUMIs* identifies 99 barcodes as intact, including all the 96 known barcodes. Also for the single-nucleus RNA-seq from Habib et al.[12] *zUMIs* identified a reasonable number of cells: Habib et al. report 10,877 nuclei and zUMIs identified 11,013 intact nuclei. However, we recommend to always check the elbow-plot generated by zUMIs (Figure 3B) to confirm that the cut-off used by zUMIs is valid for a given dataset. In cases where the number of barcodes or barcode sequences are known, it is preferable to use this information. If *zUMIs* is either given the number of expected BCs or is provided with a list of BC

sequences, it will use this information and forgo automatic inference.

Downsampling

scRNA-seq library sizes can vary by orders of magnitude, which complicates normalization [28, 29]. A straight-forward solution for this issue is to downsample over-represented libraries [30]. *zUMIs* has an inbuilt function for downsampling datasets to a user-specified number of reads or a range of reads. By default, *zUMIs* downsamples all selected barcodes to be within three absolute deviations from the median number of reads per barcode (Figure 3 C). Alternatively, the user can provide a target sequencing depth and *zUMIs* will downsample to the specified read number or omit the cell from the downsampled count table if less reads were present. Furthermore, *zUMIs* also allows to specify multiple target read number at once for downsampling. This feature is helpful, if the user wishes to determine whether the RNA-seq library was sequenced to saturation or whether further sequencing would increase the number of detected genes or UMIs enough to justify the extra cost. In our HEK-cell example dataset the number of detected genes starts leveling of at one million reads, sequencing double that amount would only increase the number of detected genes from 9,000 to 10,600, when counting Exon reads (Figure 3D). In line with previous findings [8, 14], the saturation curve of Exon+Intron counting runs parallel to the one for Exon counting, both indicating that a sequencing depth of one million reads per cell is sufficient for these libraries.

Output and Statistics

zUMIs outputs three UMI and three read count tables: genewise counts for traditional Exon counting, one for Intron and one for Exon+Intron counts. If a user chooses the downsampling option, 6 additional count tables per target read count are provided. To evaluate library quality, *zUMIs* summarizes the mapping statistics of the reads. While Exon and Intron mapping reads likely represent mRNA quantities, a high fraction of intergenic and unmapped reads indicates low-quality libraries. Another measure of RNA-seq library quality is the complexity of the library, for which the number of detected genes and the number of identified UMIs are good measures (Figure 1). We processed 227 million reads with *zUMIs* and quantified expression levels for Exon and Intron counts on a unix machine using up to 16 threads, which took barely 3 hours. Increasing the number of reads increases the processing time approximately linearly, where filtering, mapping and counting each take up roughly one third of the total time (Figure 3 E). We also observe that the peak RAM usage for processing datasets of 227, 500 and 1000 million pairs was 42 Gb, 89 Gb and 172 Gb, respectively. Finally, *zUMIs* could process the largest scRNA-seq dataset reported to date with around 1.3 million brain cells and 30 billion read pairs generated with 10xGenomics Chromium (see Methods) on a 22-core processor in only 7 days.

Intron Counting

Recently it has been shown that Intron mapping reads in RNA-seq likely originate from nascent mRNAs and are useful for gene expression estimates [31, 32]. Additionally, novel approaches leverage the ratios of Intron and Exon mapping reads to infer information on transcription dynamics and cell states La Manno et al. [33]. To address this new aspect of analysis, *zUMIs* also counts and collapses Intron-only mapping reads as well as Intron and Exon mapping reads from the same gene with the same UMI. To assess the information gain from intronic reads to estimate gene expression levels, we analyzed a publicly available DroNc-seq dataset from mouse brain ([12], see Methods). For the $\sim 11,000$ single nuclei of this dataset, the fraction of Intron mapping reads of all reads goes up to 61%. Thus, if intronic reads are considered, the mean number of detected genes per cell increases from 1041 for Exon counts to 1995 for Exon+Intron counts. Next, we used the resulting UMI count tables to investigate whether Exon+Intron counting improves the identification of cell types, as suggested in Lake et al. (2016)[11]. The validity and accuracy of counting Introns for single nucleus sequencing methods has recently been demonstrated [34]. Following the Seurat pipeline to cluster cells [35, 36], we find that using Exon+Intron counts discriminates 28 clusters, while we could only discriminate 19 clusters using Exon counts (Figure 4A+B). The larger number of clusters is not simply due to the increase in the counted UMIs and genes. When we permute the Intron counts across cells and add them to the Exon counts, the added noise actually reduces the number of identifiable clusters (Figure 4E).

We continue to further characterize the 7 clusters that were subdivided by the addition of Intron counts (Figure 4D). First, we identify differentially expressed (DE) genes between the newly formed clusters. If we count only Exon reads, there appear to be on average only 10 DE genes between the subgroups, while Exon+Intron counting yields $\sim 10\times$ more DE genes, thus corroborating the signal found with clustering. The log2-fold changes of those additional DE genes estimated with either counting strategy are generally in good agreement, especially large log2-fold changes are detected with both Exon and Exon+Intron counting (Figure 4F). Genes that are detected as DE in only one of our counting strategies have small log2-fold changes and there are more of these small changes detected using Exon+Intron counting.

Detecting more genes naturally increases the chance to also detect more informative genes. Here, we cross-reference the gene list with marker genes for transcriptomic subtypes detected for major cell types of the mouse brain [37] and find that $\sim 5\%$ of the additional genes are also marker genes. Having a closer look at cluster 7, it was split into a bigger (7) and a smaller cluster (24) using Exon+Intron counting (Figure 4A-C), we find one marker gene (Il1rapl2) to be DE between the subclusters using Exon+Intron counting, while Il1rapl2 had only spurious counts using Exon counts. Il1rapl2 is a marker for transcriptomic subtypes of GABAergic Pvalb-type neurons [37], suggesting that the split of cluster 7 might be biological meaningful (Figure 4E).

In order to evaluate the power gained by Exon+Intron counting in a more systematic way, we perform power simulations using empirical mean and dispersion distributions from the largest and most uniform cluster (~ 1500 cells) [9]. For a fair comparison, we include all detected genes and thus there are on average $4\times$ more genes in the lowest expression quantile for Exon counting than for Exon+Intron counting (Figure 4H). For those genes, expression is too spurious to be used for differential expression analysis, while for Exon+Intron counting we have on average 60% power to detect a DE gene in the first mean expression bin with a well controlled FDR (Figure 4G). In summary, the increased power for Exon+Intron counting and probably also the larger number of clusters is due to a better detection of lowly expressed genes. Furthermore, we think that, although potentially noisy, the large number of additionally detected genes makes Exon+Intron counting worthwhile, especially for single-nuclei sequencing techniques that are enriched for nuclear nascent RNA transcripts, such as DroNc-seq [12]. Additionally, Exon+Intron counting may help extracting as much information as possible from low coverage data as generated in the context of high-throughput cell atlas efforts (eg 10,000-20,000 reads/cell [38, 39]. Lastly, users should always exclude the possibility of intronic reads stemming from genomic DNA contamination in the library preparation by confirming low intergenic mapping fractions using the statistics output provided by *zUMIs*.

Conclusion

zUMIs is a fast and flexible pipeline processing raw reads to obtain count tables for RNA-seq data using UMIs. To our knowledge it is the only open source pipeline that has a barcode and UMI quality filter, allows Intron counting and has an integrated downsampling functionality. These features ensure that *zUMIs* is applicable to most experimental designs of RNA-seq data, including single nucleus sequencing techniques, droplet-based methods where the BC is unknown, as well as plate-based UMI-methods with known BCs. Finally, *zUMIs* is computationally efficient, user-friendly and easy to install.

Methods

Analysed RNA-seq datasets

HEK293T cells were cultured in DMEM High Glucose with L-Glutamine (Biowest) supplemented with 10 % Fetal Bovine Serum (Thermo Fisher) and 1 % Penicillin/Streptomycin (Sigma-Aldrich) in a 37 °C incubator with 5 % CO2. Cells were passaged and split every 2 or 3 days. For single-cell RNA-seq, HEK293T cells were dissociated by incubation with 0.25 % Trypsin (Sigma-Aldrich) for 5 minutes at 37 °C. The single-cell suspension was washed twice with PBS and dead cells stained with Zombie Yellow (Biolegend) according to the manufacturer's protocol. Single-cells were sorted into DNA LoBind 96-well PCR plates (Eppendorf) containing lysis buffer with a Sony SH-800 cell sorter in 3-drop purity mode using a 100 µm nozzle. Next, single-cell RNA-seq libraries were constructed from one 96-well plate using a slightly modified version of the mcSCRB-seq protocol. Reverse transcription was performed as described previously [40], with the only change being the use of KAPA HiFi HotStart enzyme for PCR amplification of cDNA. Resulting libraries were sequenced using an Illumina HiSeq1500 with 16 cycles in Read 1 to decode cell barcodes (6 bases) and UMIs (10 bases) and 50 cycles in Read 2 to sequence into the cDNA fragment, obtaining ~ 227 million reads. Raw fastq files were processed using *zUMIs*, mapping to the human genome (hg38) and Ensembl gene models (GRCh38.84).

Furthermore, we anlysed data from 1.3 million mouse brain cells generated on the 10xGenomics Chromium platform [2]. Sequences were downloaded from the NCBI Sequence Read Archive under accession number SRP096558. The data consist of 30 billion read pairs from 133 individual samples. In these data, read 1 contains 16 bp for the cell barcode and 10 bp for the UMI and read 2 contains 114 bp of cDNA. *zUMIs* was run using default settings and we allowed 7 threads per job for a total of up to 42 threads on an Intel Xeon E5-2699 22-core processor.

Finally, we obtained mouse brain DroNc-seq read data [12] from the Broad Institute Single Cell Portal (https://portals.broadinstitute.org/single_cell/study/dronc-seq-single-nucleus-rna-seq-on-mouse-archived-brain). This dataset consists of ~1615 million read pairs from ~ 11,000 single nuclei. Read 1 contains a 12bp cell barcode and a 8bp UMI and read 2 60bp of cDNA.

The two mouse datasets were mapped to genome version mm10 and applying Ensembl gene models (GRCm38.75).

Power simulations and DE analysis

We evaluated the power to detect differential expression with the help of the powsimR package [9]. For the DroNc-seq dataset, we estimated the parameters of the negative binomial distribution from one of the identified clusters, namely cluster 0, compromising 1500 glutamatergic neuronal cells from the prefrontal cortex (Figure 4D). Since we detect more genes with Exon+Intron counting (4433 compared to 1782), we included this phenomenon also in our read count simulation by drawing mean expression values for a total of 4433 genes. This means that the table includes sparse counts for the Exon counting. Log2 fold changes were drawn from a gamma distribution with shape equal to 1 and scale equal to 2. In each of the 25 simulation iterations, we draw an equal sample size of 300 cells per group and test for differential expression using limma-trend [41] on log2 CPM values with scran [42] library size correction. The TPR and FDR are stratified over the empirical mean expression quantile bins.

For the differential expression analysis between clusters, we use the same DE estimation procedure as in the simulations: scran normaliztion followed by limma-trend DE-analysis (c.f. [43]).

Cluster Identification

After processing the DroNc-seq data [12] with zUMIs as described above, we cluster cells based on UMI counts derived from Exons only and Exons+Introns reads using the Seurat pipeline [35, 36]. First, cells with fewer than 200 detected genes were filtered out. The filtered data were normalized using the 'LogNormalize' function. We then scale the data by regressing out the effects of the number of transcripts and genes detected per cell using the 'ScaleData' function. The normalized and scaled data are then used to identify the most variable genes by fitting a relationship between mean expression (ExpMean) and dispersion (LogVMR) using the 'FindVariableGenes' function. The identified variable genes are used for Principle Component Analysis (PCA) and the top 20 PCs are then used to find clusters using graph based clustering as implemented in 'FindClusters'. To illustrate that the additional clusters found by counting Exon+Intron reads are not spurious, we use Intron-only UMI-counts from the same data to add to the observed Exon only counts. More specifically, to each gene we add scran-sizeFactor corrected Intron counts from the same gene after permuting them across cells. We assessed the cluster numbers from 100 such permutations.

Comparison of UMI collapsing strategies

In order to validate *zUMIs* and compare different UMI collapsing methods, we used the HEK dataset described above. We ran *zUMIs* (1) without quality filtering, (2) filtering for 1 base under Phred 17 and (3) collapsing similar UMI sequences within a hamming distance of 1. To compare with other available tools, we ran the same dataset using the Drop-seq-tools version 1.13 [13] and quality filter "1 base under Phred 17" without edit distance collapsing. Lastly, the HEK dataset was used with UMI-tools [25] in (1) "unique" and (2) "directional adjacency" mode with edit distance set to 1. Furthermore, we compared the output of *zUMIs* from the DroNc-seq dataset when using default parameters ("1 base under Phred 20") to UMI-tools in (1) "unique", (2) "directional adjacency" and (3) "cluster" settings. For each setting and tool combination, we compared per-cell/per-nuclei UMI contents in a linear model fit.

Availability of Source Code and Requirements

- Project name: zUMIs
- Project home page: https://github.com/sdparekh/zUMIs
- Operating system(s): UNIX
- Programming language: shell, R, perl
- Other requirements: STAR >= 2.5.3a, R >= 3.4, Rsubread >= 1.26.1, pigz >= 2.3 & samtools >= 1.1
- License: GNU GPLv3.0
- Research Resource Identification Initiative ID: SCR_016139

Availability of supporting data and materials

All data that were generated for this project were submitted to GEO under accession GSE99822.

Declarations

List of Abbreviations

scRNA-seq - single-cell RNA-sequencing
UMI - Unique Molecular Identifier
BC - Barcode
MAD - Median Absolute Deviation

Competing Interests

The author(s) declare that they have no competing interests.

Funding

This work has been supported by the DFG through SFB1243 subprojects A14/A15.

Author's Contributions

SP and CZ designed and implemented the pipeline. BV tested the pipeline and helped in power simulations. All authors contributed to writing the manuscript.

References

1. Sandberg R. Entering the era of single-cell transcriptomics in biology and medicine. Nat Methods 2014 Jan;11(1):22–24.
2. Zheng GXY, Terry JM, Belgrader P, Ryvkin P, Bent ZW, Wilson R, et al. Massively parallel digital transcriptional profiling of single cells. Nat Commun 2017 16 Jan;8:14049.
3. Rosenberg AB, Roco CM, Muscat RA, Kuchina A, Sample P, Yao Z, et al. Single-cell profiling of the developing mouse brain and spinal cord with split-pool barcoding. Science 2018 Mar;p. eaam8999.
4. Wagner A, Regev A, Yosef N. Revealing the vectors of cellular identity with single-cell genomics. Nat Biotechnol 2016 8 Nov;34(11):1145–1160.
5. Regev A, Teichmann SA, Lander ES, Amit I, Benoist C, Birney E, et al. The Human Cell Atlas. Elife 2017 Dec;6.
6. Parekh S, Ziegenhain C, Vieth B, Enard W, Hellmann I. The impact of amplification on differential expression analyses by RNA-seq. Sci Rep 2016 9 May;6:25533.
7. Kivioja T, Vähärautio A, Karlsson K, Bonke M, Enge M, Linnarsson S, et al. Counting absolute numbers of molecules using unique molecular identifiers. Nat Methods 2012 Jan;9(1):72–74.
8. Ziegenhain C, Vieth B, Parekh S, Reinius B, Guillaumet-Adkins A, Smets M, et al. Comparative Analysis of Single-Cell RNA Sequencing Methods. Mol Cell 2017 16 Feb;65(4):631–643.e4.
9. Vieth B, Ziegenhain C, Parekh S, Enard W, Hellmann I. powsimR: Power analysis for bulk and single cell RNA-seq experiments. Bioinformatics 2017 Jul;.
10. Ziegenhain C, Vieth B, Parekh S, Hellmann I, Enard W. Quantitative single-cell transcriptomics. Brief Funct Genomics 2018 Mar;.
11. Lake BB, Ai R, Kaeser GE, Salathia NS, Yung YC, Liu R, et al. Neuronal subtypes and diversity revealed by single-nucleus RNA sequencing of the human brain. Science 2016 24 Jun;352(6293):1586–1590.
12. Habib N, Avraham-Davidi I, Basu A, Burks T, Shekhar K, Hofree M, et al. Massively parallel single-nucleus RNA-seq with DroNc-seq. Nat Methods 2017 Oct;14(10):955–958.
13. Macosko EZ, Basu A, Satija R, Nemesh J, Shekhar K, Goldman M, et al. Highly Parallel Genome-wide Expression Profiling of Individual Cells Using Nanoliter Droplets. Cell 2015 21 May;161(5):1202–1214.
14. Svensson V, Natarajan KN, Ly LH, Miragaia RJ, Labalette C, Macaulay IC, et al. Power analysis of single-cell RNA-sequencing experiments. Nat Methods 2017 6 Mar;.
15. Hashimshony T, Senderovich N, Avital G, Klochendler A, de Leeuw Y, Anavy L, et al. CEL-Seq2: sensitive highly-multiplexed single-cell RNA-Seq. Genome Biol 2016 28 Apr;17(1):77.
16. Petukhov V, Guo J, Baryawno N, Severe N, Scadden D, Samsonova MG, et al. Accurate estimation of molecular counts in droplet-based single-cell RNA-seq experiments. bioRxiv 2017 Sep;p. 171496.
17. Soumillon M, Cacchiarelli D, Semrau S, van Oudenaarden A, Mikkelsen TS. Characterization of directed differentiation by high-throughput single-cell RNA-Seq. bioRxiv 2014 5 Mar;.
18. Jaitin DA, Kenigsberg E, Keren-Shaul H, Elefant N, Paul F, Zaretsky I, et al. Massively parallel single-cell RNA-seq for marker-free decomposition of tissues into cell types. Science 2014 14 Feb;343(6172):776–779.
19. Klein AM, Mazutis L, Akartuna I, Tallapragada N, Veres A, Li V, et al. Droplet barcoding for single-cell transcriptomics applied to embryonic stem cells. Cell 2015 21 May;161(5):1187–1201.
20. Zilionis R, Nainys J, Veres A, Savova V, Zemmour D, Klein AM, et al. Single-cell barcoding and sequencing using droplet microfluidics. Nat Protoc 2017 Jan;12(1):44–73.
21. Hochgerner H, Lönnerberg P, Hodge R, Mikes J, Heskol A, Hubschle H, et al. STRT-seq-2i: dual-index 5' single cell and nucleus RNA-seq on an addressable microwell array. Sci Rep 2017 Nov;7(1):16327.
22. Dobin A, Davis CA, Schlesinger F, Drenkow J, Zaleski C, Jha S, et al. STAR: ultrafast universal RNA-seq aligner. Bioinformatics 2013 1 Jan;29(1):15–21.
23. Liao Y, Smyth GK, Shi W. featureCounts: an efficient general purpose program for assigning sequence reads to genomic features. Bioinformatics 2014 1 Apr;30(7):923–930.
24. Dowle M, Srinivasan A. data.table: Extension of 'data.frame'; 2017, https://CRAN.R-project.org/package=data.table, r package version 1.10.4.
25. Smith TS, Heger A, Sudbery I. UMI-tools: Modelling sequencing errors in Unique Molecular Identifiers to improve quantification accuracy. Genome Res 2017 18 Jan;.
26. Fraley C, Raftery AE. Model-Based Clustering, Discriminant Analysis, and Density Estimation. J Am Stat Assoc 2002 Jun;97(458):611–631.
27. Fraley C, Raftery AE, Brendan Murphy T, Scrucca L. mclust Version 4 for R: Normal Mixture Modeling for Model-Based Clustering, Classification, and Density Estimation. 2012;.
28. Vallejos CA, Risso D, Scialdone A, Dudoit S, Marioni JC. Normalizing single-cell RNA sequencing data: challenges and opportunities. Nat Methods 2017 Jun;14(6):565–571.
29. Evans C, Hardin J, Stoebel DM. Selecting between-sample RNA-Seq normalization methods from the perspective of their assumptions. Brief Bioinform 2017 27 Feb;.
30. Grün D, van Oudenaarden A. Design and Analysis of Single-

Cell Sequencing Experiments. Cell 2015 5 Nov;163(4):799–810.

31. Hendriks GJ, Gaidatzis D, Aeschimann F, Großhans H. Extensive oscillatory gene expression during C. elegans larval development. Mol Cell 2014 Feb;53(3):380–392.
32. Gaidatzis D, Burger L, Florescu M, Stadler MB. Analysis of intronic and exonic reads in RNA-seq data characterizes transcriptional and post-transcriptional regulation. Nat Biotechnol 2015 Jul;33(7):722–729.
33. La Manno G, Soldatov R, Hochgerner H, Zeisel A, Petukhov V, Kastriti M, et al. RNA velocity in single cells. bioRxiv 2017 Oct;p. 206052.
34. Lake BB, Codeluppi S, Yung YC, Gao D, Chun J, Kharchenko PV, et al. A comparative strategy for single-nucleus and single-cell transcriptomes confirms accuracy in predicted cell-type expression from nuclear RNA. Sci Rep 2017 Jul;7(1):6031.
35. Satija R, Farrell JA, Gennert D, Schier AF, Regev A. Spatial reconstruction of single-cell gene expression data. Nat Biotechnol 2015 May;33(5):495–502.
36. Butler A, Satija R. Integrated analysis of single cell transcriptomic data across conditions, technologies, and species. bioRxiv 2017 Jul;p. 164889.
37. Tasic B, Menon V, Nguyen TN, Kim TK, Jarsky T, Yao Z, et al. Adult mouse cortical cell taxonomy revealed by single cell transcriptomics. Nat Neurosci 2016 Feb;19(2):335–346.
38. The Tabula Muris Consortium, Quake SR, Wyss-Coray T, Darmanis S. Single-cell transcriptomic characterization of 20 organs and tissues from individual mice creates a Tabula Muris. bioRxiv 2018 Mar;p. 237446.
39. Han X, Wang R, Zhou Y, Fei L, Sun H, Lai S, et al. Mapping the Mouse Cell Atlas by Microwell-Seq. Cell 2018 Feb;172(5):1091–1107.e17.
40. Bagnoli JW, Ziegenhain C, Janjic A, Wange LE, Vieth B, Parekh S, et al. mcSCRB-seq: sensitive and powerful single-cell RNA sequencing. bioRxiv 2017 Oct;p. 188367.
41. Law CW, Chen Y, Shi W, Smyth GK. voom: Precision weights unlock linear model analysis tools for RNA-seq read counts. Genome Biol 2014 Feb;15(2):R29.
42. Lun ATL, McCarthy DJ, Marioni JC. A step-by-step workflow for low-level analysis of single-cell RNA-seq data with Bioconductor. F1000Res 2016 Aug;5:2122.
43. Soneson C, Robinson MD. Bias, robustness and scalability in single-cell differential expression analysis. Nat Methods 2018 Apr;15(4):255–261.
44. Grün D, Kester L, van Oudenaarden A. Validation of noise models for single-cell transcriptomics. Nat Methods 2014 Jun;11(6):637–640.
45. Tian L, Su S, Amann-Zalcenstein D, Biben C, Naik SH, Ritchie ME. scPipe: a flexible data preprocessing pipeline for single-cell RNA-sequencing data. bioRxiv 2017 Aug;p. 175927.
46. Islam S, Zeisel A, Joost S, La Manno G, Zajac P, Kasper M, et al. Quantitative single-cell RNA-seq with unique molecular identifiers. Nat Methods 2014 Feb;11(2):163–166.

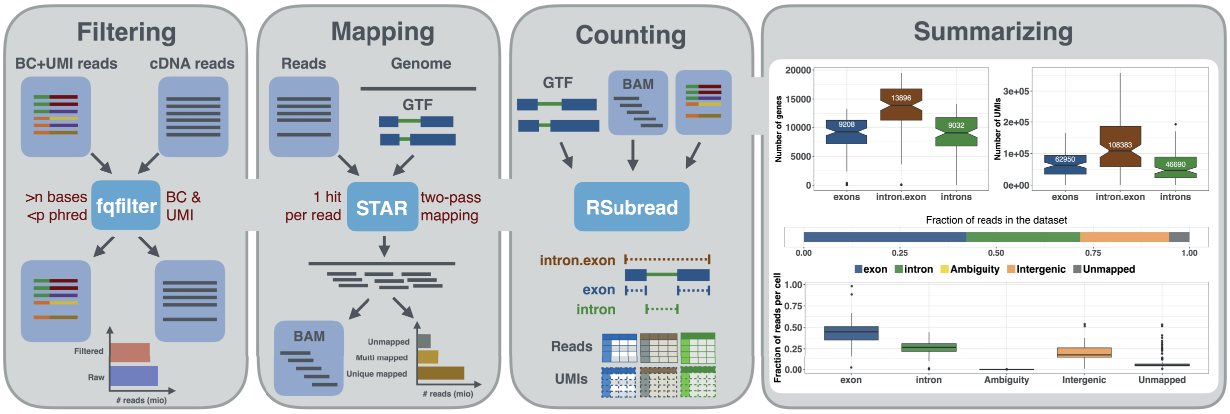

Figure 1. Schematic of the zUMIs pipeline. Each of the grey panels from left to right depicts a step of the *zUMIs* pipeline. First, fastq files are filtered according to user-defined barcode (BC) and unique molecular identifier (UMI) quality thresholds. Next, the remaining cDNA reads are mapped to the reference genome using STAR. Gene-wise read and UMI count tables are generated for Exon , Intron and Exon+Intron overlapping reads. To obtain comparable library sizes, reads can be downsampled to a desired range during the counting step. In addition, *zUMIs* also generates data and plots for several quality measures, such as the number of detected genes/UMIs per barcode and distribution of reads into mapping feature categories.

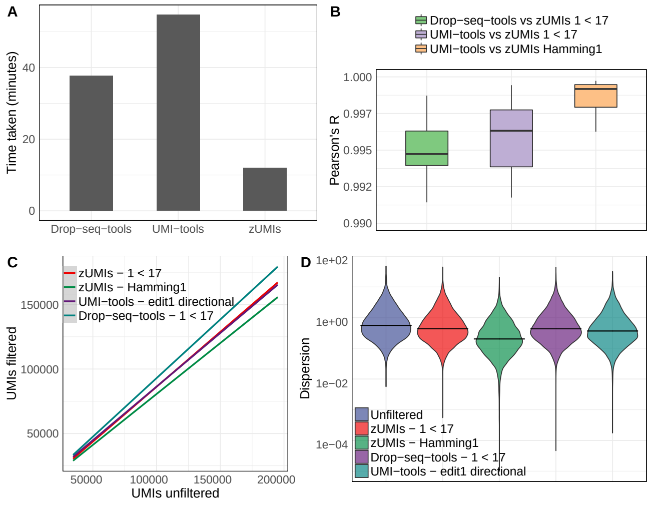

Figure 2. Comparison of different UMI collapsing methods. We compared Drop-seq-tools and UMI-tools with zUMIs using our HEK dataset (227 mio reads). **(A)** Runtime to count exonic UMIs using zUMIs (hamming distance = 0), UMI-tools ("unique" mode) and Drop-seq-tools (edit distance = 0). **(B)** Boxplots of correlation coefficients of gene-wise UMI counts of the same cell generated with different methods. UMI counts generated using zUMIs (quality filter "1 base under phred 17" or hamming distance = 1) were correlated to UMI counts generated using Drop-seq-tools (quality filter "1 base under phred 17") and UMI-tools ("directional adjacency" mode). **C)** Comparison of the total number of UMIs per cell derived from different counting methods to "unfiltered" counts. **(D)** Violin plots of gene-wise dispersion estimates with different quality filtering and UMI collapsing methods.

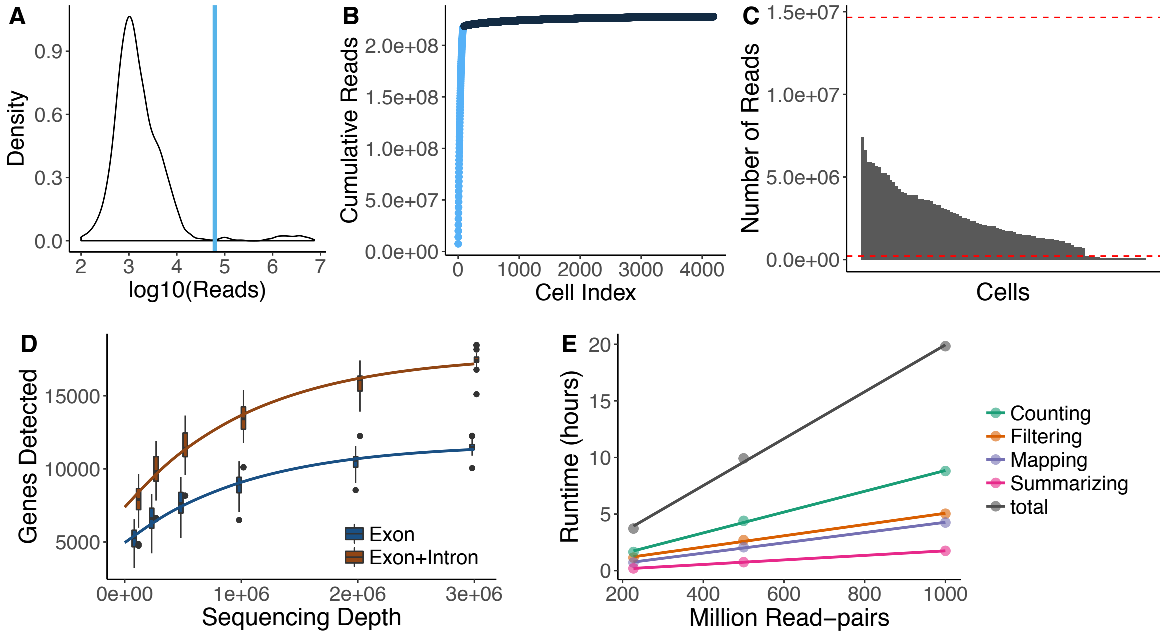

Figure 3. Utilities of zUMIs. Each of the panels shows the utilities of *zUMIs* pipeline. The plots from A-D are the results from the example HEK dataset used in the paper. A) The plot shows a density distribution of reads per barcode. Cell barcodes with reads above the blue line are selected. B) The plot shows the cumulative read distribution in the example HEK dataset where the barcodes in light blue are the selected cells. C) The barplot shows the number of reads per selected cell barcode with the red lines showing upper and lower MAD (Median Absolute Deviations) cutoffs for adaptive downsampling. Here, the cells below the lower MAD have very low coverage and are discarded in downsampled count tables. D) Cells were downsampled to six depths from 100,000 to 3,000,000 reads. For each sequencing depth the genes detected per cell is shown. E) Runtime for three datasets with 227, 500 and 1000 million read-pairs. The runtime is divided in the main steps of the *zUMIs* pipeline: Filtering, Mapping, Counting and Summarizing. Each dataset was processed using 16 threads ("-p 16").

Name	Reference	Open Source	Quality filter	UMI collapsing	Mapper	BC detection	Intron	Downsampling	Compatible UMI library protocols
Cell Ranger	[2]	yes	BC+UMI	Hamming distance	STAR	A	no	yes	[2]
CEL-seq	[15]	yes	BC+UMI	identity only	bowtie2	WL	no	no	[44, 15]
dropEst	[16]	yes	BC	frequency-based	TopHat2 or Kallisto	WL,top-n,EM	yes	no	[13, 19, 2]
Drop-seq-tools	[13]	no	BC+UMI	Hamming distance	STAR	WL,top-n	no	no	[13, 17, 15]
scPipe	[45]	yes	BC+UMI	Hamming distance	subread	WL,top-n	no	no	[44, 18, 17, 13]
umis	[14]	yes	BC	frequency-based	Kallisto	WL,top-n,EM	no	no	[17, 44, 46, 18, 13, 19, 2]
UMI-tools	[25]	yes	BC+UMI	network-based	BWA	WL	no	no	[17, 19]
zUMIs	This work	yes	BC+UMI	Hamming distance	STAR	A,WL,top-n	yes	yes	[17, 44, 46, 18, 13, 15, 21, 12, 3, 2]

Table 1. Features of available UMI pipelines for the quantification of gene expression data. We consider whether the pipeline is open source, has sequence quality filters for cell barcodes (BC) and UMIs, mappers, UMI-collapsing options, options for BC detection (A - automatically infer intact BCs, WL - extract only the given list of known BCs, top-n - order BCs according the number of reads and keep the top n BCs, EM - merge BCs with given edit-distance), whether it can count Intron mapping reads, whether it offers a utility to make varying library sizes more comparable via downsampling and finally with which RNA-seq library preparation protocols it is compatible.

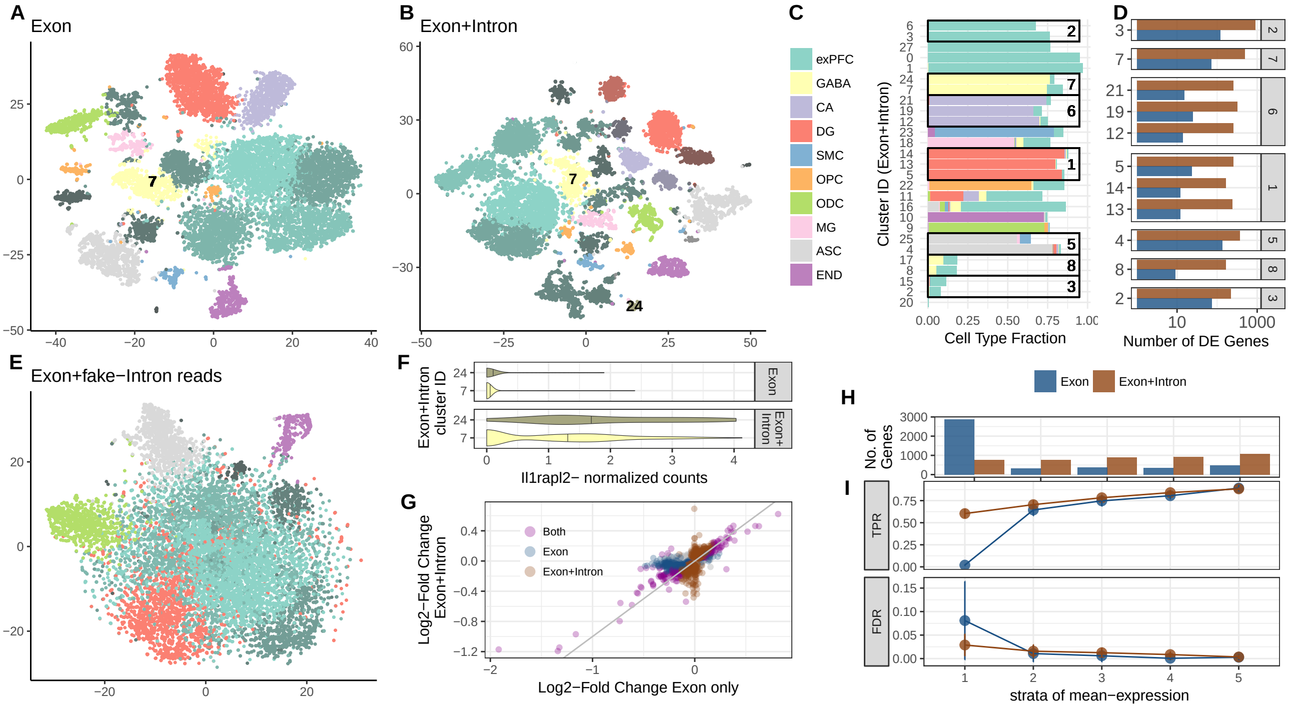

Figure 4. Contribution of Intron reads to biological insights. We analyse published single-nucleus RNA-seq data from mouse prefrontal cortex (PFC) and hippocampus [12] to assess the utility of counting Intron in addition to Exon reads. We processed the raw data with *zUMIs* to obtain expression tables with Exon reads as well as Exon+Intron reads and then use the R-package Seurat [35, 36] to cluster cells. With Exon counts, we thus identify 19 clusters **(A)** and with Exon+Intron counts 27 **(B)**. Clusters are represented as t-SNE plots and colored according to the most frequent cell-type assignment in the original paper [12]: glutamatergic neurons from the prefrontal cortex (exPFC), GABAergic interneurons (GABA), pyramidal neurons from the hippocampal CA region (CA), granule neurons from the hippocampal dentate gyrus region (DG), astrocytes (ASC), microglia (MG), oligodendrocytes (ODC), oligodendrocyte precursor cells (OPC), neuronal stem cells (NSC), smooth muscle cells (SMC) and endothelial cells (END). Different shades of those clusters indicate that multiple clusters had the same major cell-type assigned. If we randomly sample counts from the intron data and add them to the Exon counting, the noise reduces the number of clusters and the Seurat pipeline can only identify 9-11 clusters **(E)**. The composition of each cluster based on Exon+Intron is detailed in panel **(C)** and cells that were not assigned a cell type in Habib et al. [12] are displayed as empty. The boxes mark the clusters that were not split when using Exon data only. For example, cluster 7 from Exon counting that mainly consists of GABAergic neurons, was split into clusters 7, 24 (506, 66 cells) when using Exon+Intron counting. In **(D)**, we show the numbers of genes that were DE (limma p-adj<0.05) between the clusters only found with Exon+Intron counts. The panel numbers represent the Exon counting cluster numbers and the y-axis the Exon+Intron counting cluster number. The log2-fold changes corresponding to these contrasts are also used in **G)**. Among the genes that were additionally detected to be DE by Exon+Intron counting was the marker gene Il1rapl2 (limma p-adj=10^{-5}). In **(F)**, we present a violin plot of the normalized counts for Il1rapl2 in cells of the GABAergic subclusters 7 and 24. Log2-fold-changes calculated with Exon+Intron counts correlate well with Exon counts **(G)**. Note that for Exon counting only half as many genes could be evaluated as for Exon+Intron counting and thus only half of the Exon+Intron genes are depicted in **(G)**. Large LFCs are found significant with both counting strategies (purple points are close to the bisecting line). We conduct simulations based on mean and dispersion measured using Exon cluster 0 (1616 cells, ~ 90% exPFC). In **(I)** we show the expected true positive rate (TPR) and the false discovery rate (FDR) for a scenario comparing 300 vs 300 cells. Results for Exon and Exon+Intron counting were stratified into 5 quantiles according to the mean expression of genes, where stratum 1 contains lowly expressed genes and stratum 5 the most highly expressed genes. The numbers of genes falling into each of the bins using Exon+Intron and Exon counting are depicted in **(H)**.

Figure 1

Click here to download Figure zUMI_pipeline.eps

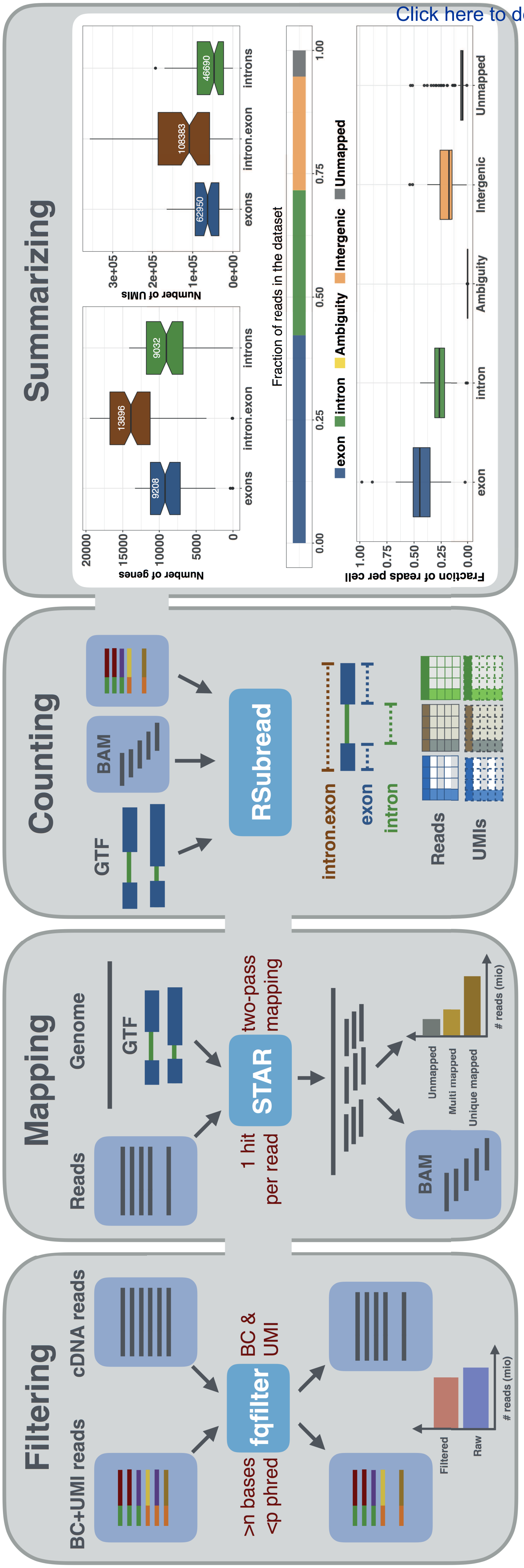

Figure 2

Click here to download Figure figure2.pdf

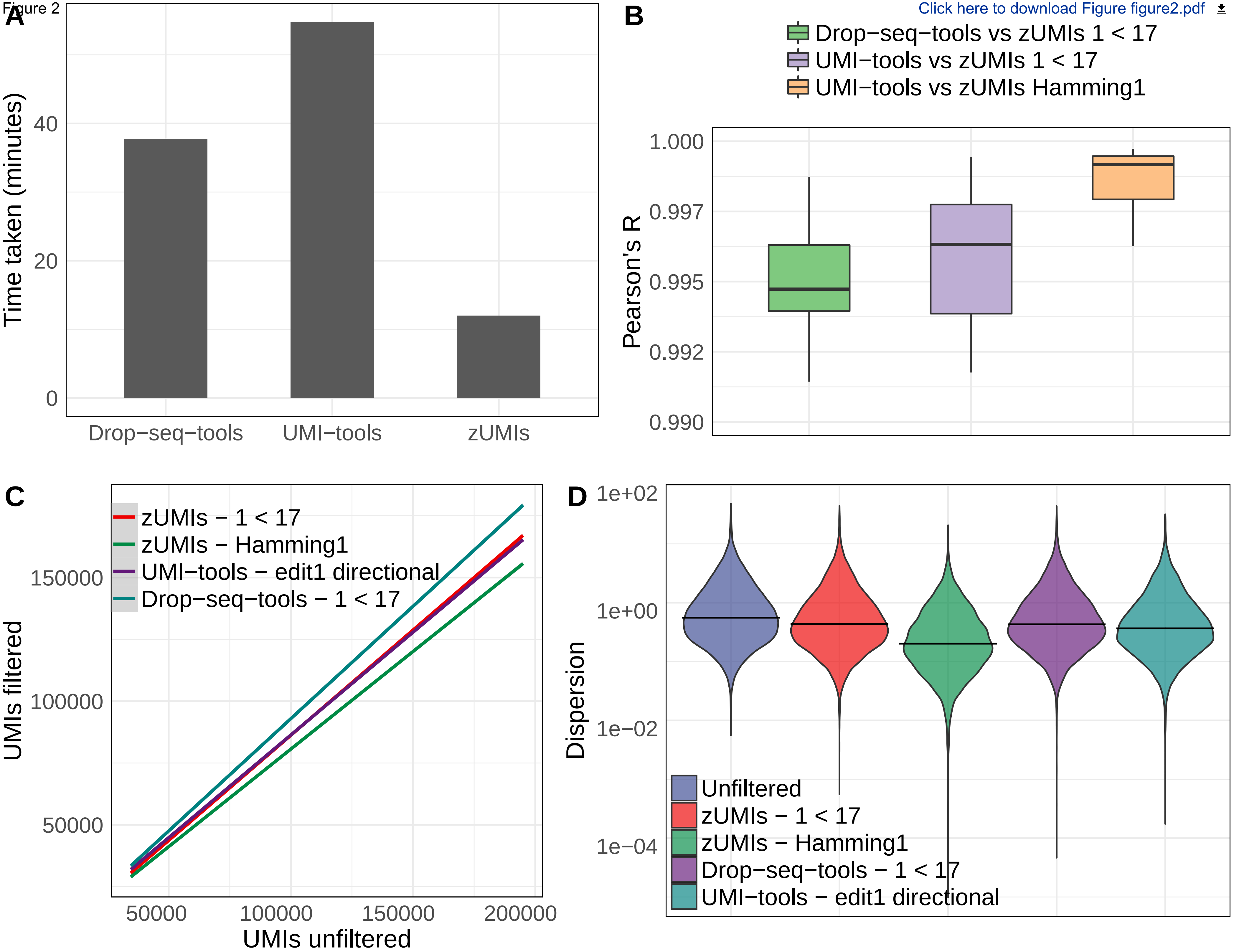

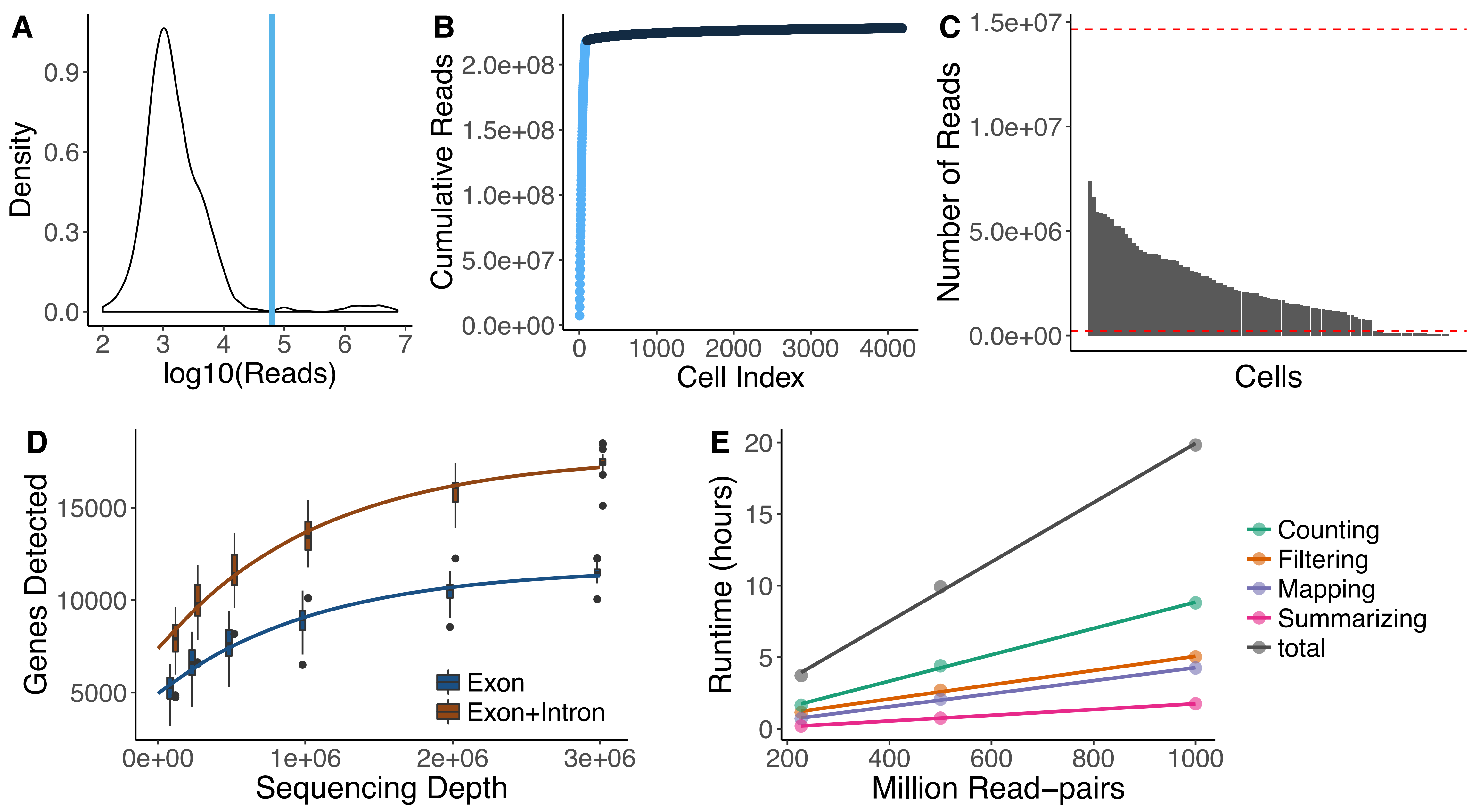
A
Density
0.0
0.3
0.6
0.9
2
3
4
5
6
7
log10(Reads)
B
Cumulative Reads
0.0e+00
5.0e+07
1.0e+08
1.5e+08
2.0e+08
0
1000
2000
3000
4000
Cell Index
C
Number of Reads
0.0e+00
5.0e+06
1.0e+07
1.5e+07
Cells
D
Genes Detected
5000
10000
15000
0e+00
1e+06
2e+06
3e+06
Sequencing Depth
Exon
Exon+Intron
E
Runtime (hours)
0
5
10
15
20
200
400
600
800
1000
Million Read−pairs
Counting
Filtering
Mapping
Summarizing
total

Figure 4

Click here to download Figure figure4v3.pdf

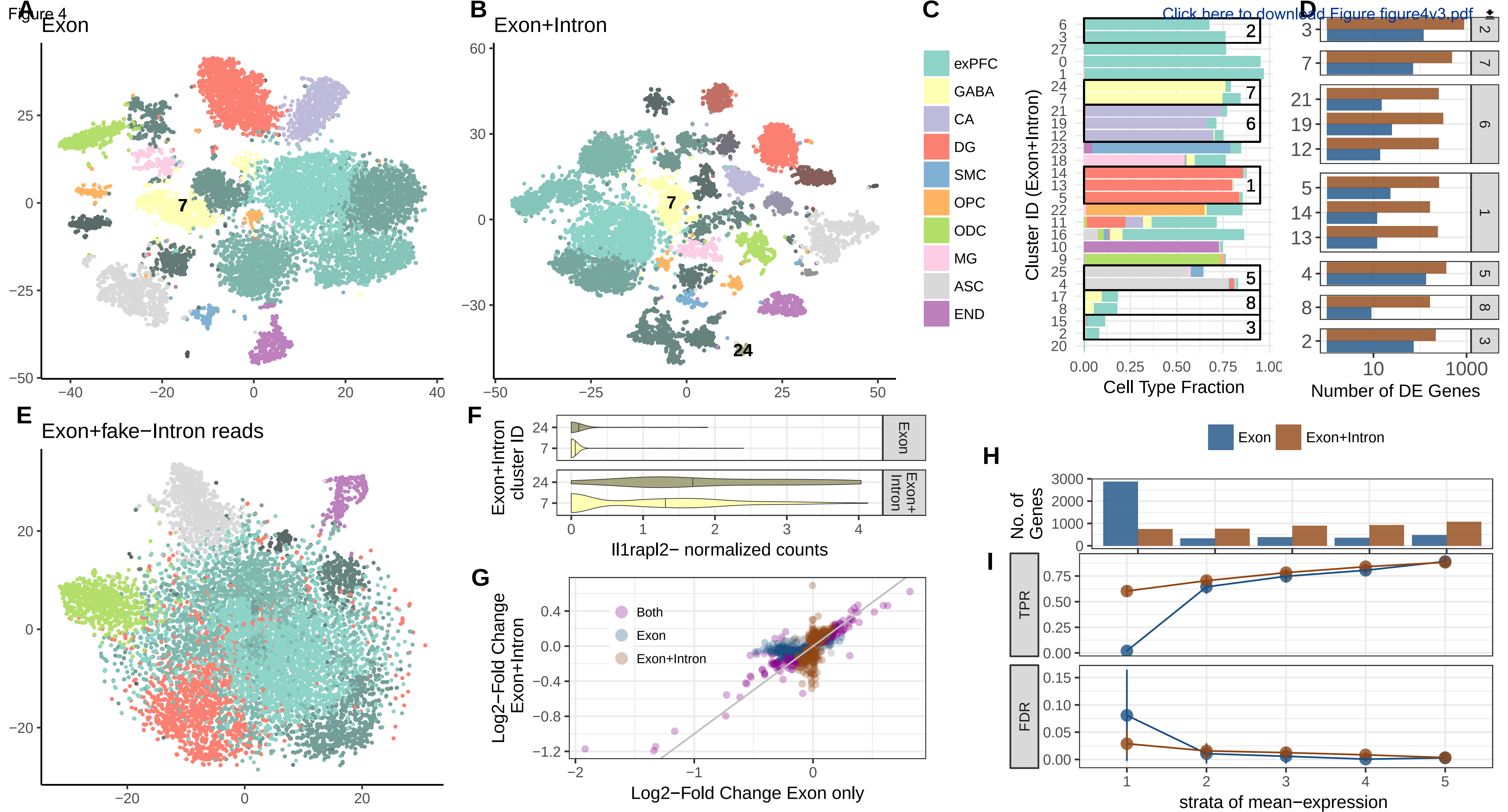

Reference PDF

Click here to access/download

Supplementary Material

zumis_fast_flexible_R2.pdf
