# Supplementary material for: zUMIs - A fast and flexible pipeline to process RNA sequencing data with UMIs
Source: Gigascience. 2018 May 26;7(6):giy059. doi: 10.1093/gigascience/giy059 (PMC6007394; doi:10.1093/gigascience/giy059)
Supplement: GIGA-D-17-00271_Revision_3.pdf [file giy059_giga-d-17-00271_revision_3.pdf]

GigaScience

zUMIs - A fast and flexible pipeline to process RNA sequencing data with UMIs

--Manuscript Draft--

**Manuscript Number:**	GIGA-D-17-00271R3
**Full Title:**	zUMIs - A fast and flexible pipeline to process RNA sequencing data with UMIs
**Article Type:**	Technical Note
**Funding Information:**	Deutsche Forschungsgemeinschaft (SFB1243 - A15) — Dr. Ines Hellmann<br>Deutsche Forschungsgemeinschaft (SFB1243 - A14) — Prof. Wolfgang Enard
**Abstract:**	Single cell RNA-seq (scRNA-seq) experiments typically analyze hundreds or thousands of cells after amplification of the cDNA. The high throughput is made possible by the early introduction of sample-specific barcodes (BCs) and the amplification bias is alleviated by unique molecular identifiers (UMIs). Thus the ideal analysis pipeline for scRNA-seq data needs to efficiently tabulate reads according to both BC and UMI.<br>zUMIs is such a pipeline, it can handle both known and random BCs and also efficiently collapses UMIs, either just for exon mapping reads or for both exon and intron mapping reads. Another unique feature of zUMIs is the adaptive downsampling function, that facilitates dealing with hugely varying library sizes, but also allows to evaluate whether the library has been sequenced to saturation. zUMIs flexibility allows to accommodate data generated with any of the major scRNA-seq protocols that use BCs and UMIs. To illustrate the utility of zUMIs, we analysed a single-nucleus RNA-seq dataset and show that more than 35% of all reads map to introns. We furthermore show that these intronic reads are informative about expression levels, significantly increasing the number of detected genes and improving the cluster resolution.
**Corresponding Author:**	Ines Hellmann<br>Ludwig-Maximilians-Universitat Munchen Fakultat fur Biologie<br>Martinsried, GERMANY
**Corresponding Author Secondary Information:**	
**Corresponding Author's Institution:**	Ludwig-Maximilians-Universitat Munchen Fakultat fur Biologie
**Corresponding Author's Secondary Institution:**	
**First Author:**	Swati Parekh
**First Author Secondary Information:**	
**Order of Authors:**	Swati Parekh<br>Christoph Ziegenhain<br>Beate Vieth<br>Wolfgang Enard<br>Ines Hellmann
**Order of Authors Secondary Information:**	
**Response to Reviewers:**	In order to increase readability, we have included only the remaining questions of the reviewer<br><br>***<br>REVIEWER RESPONSE 2:<br>Please clarify "all detected genes". Is this all genes detected in Exon or Exon+Intron? If including all detected genes, then why are there different numbers of genes in each quantile in Figure 4H (top panel)? By definition, a quantile bin should contain roughly equal numbers of observations, but this does not seem to be the case even if the

	quantiles were defined on the Exon+Intron data (the larger quantile bins have more genes for Exon+Intron).<br>***<br><br>---<br>Author response:<br>We have amended the text to clarify this point as requested.<br>"For a fair comparison, we include all detected genes, which is equivalent to the number of genes detected with Exon+Intron counting and since we call a gene detected as soon as one count is associated, Exon counting is necessarily a subset of Exon+Intron."<br>---<br><br>***<br>REVIEWER RESPONSE 2:<br>Thanks for the clarification regarding enrichment of marker genes as opposed to simply detecting more genes in general. I appreciate the explanation given by the paragraph in the authors' response beginning with "Concerning the additionally detected marker genes:...". It would be transparent to include this explanation in the manuscript, since as it reads currently, it seems to imply an enrichment for marker genes when including introns.<br>***<br><br>---<br>Author response:<br>We agree with making the explanation as transparent as possible and have amended the text in this regard.<br>"Here, we cross-reference the gene list with marker genes for transcriptomic subtypes detected for major cell types of the mouse brain and find that ~5% of the additional genes are also marker genes, which corresponds well to the general frequency of marker genes among the detected genes (4%). In the same vein, we also detect proportionally more DE genes with Exon+Intron counting as compared to Exon counting. Thus including introns simply allows us to better detect present transcripts, while it leaves the proportions of interest unaltered."<br>---
**Additional Information:**	
**Question**	**Response**
Are you submitting this manuscript to a special series or article collection?	No
**Experimental design and statistics**<br><br>Full details of the experimental design and statistical methods used should be given in the Methods section, as detailed in our Minimum Standards Reporting Checklist. Information essential to interpreting the data presented should be made available in the figure legends.<br><br>Have you included all the information requested in your manuscript?	Yes
**Resources**<br><br>A description of all resources used, including antibodies, cell lines, animals and software tools, with enough	Yes

information to allow them to be uniquely identified, should be included in the Methods section. Authors are strongly encouraged to cite Research Resource Identifiers (RRIDs) for antibodies, model organisms and tools, where possible.<br><br>Have you included the information requested as detailed in our Minimum Standards Reporting Checklist?	
**Availability of data and materials**<br><br>All datasets and code on which the conclusions of the paper rely must be either included in your submission or deposited in publicly available repositories (where available and ethically appropriate), referencing such data using a unique identifier in the references and in the "Availability of Data and Materials" section of your manuscript.<br><br>Have you have met the above requirement as detailed in our Minimum Standards Reporting Checklist?	Yes

Manuscript Click here to download Manuscript main.tex

```
This is pdfTeX, Version 3.14159265-2.6-1.40.16 (TeX Live 2015/W32TeX)
(preloaded format=pdflatex 2016.4.6)  22 APR 2018 10:20
entering extended mode
 restricted \write18 enabled.
 %&-line parsing enabled.
**./main.tex
(./main.tex
LaTeX2e <2016/03/31>
Babel <3.9q> and hyphenation patterns for 81 language(s) loaded.
(./oup-contemporary.cls
Document Class: oup-contemporary 2017/06/28, v1.1
(c:/TeXLive/2015/texmf-dist/tex/latex/base/article.cls
Document Class: article 2014/09/29 v1.4h Standard LaTeX document class
(c:/TeXLive/2015/texmf-dist/tex/latex/base/size10.clo
File: size10.clo 2014/09/29 v1.4h Standard LaTeX file (size option)
)
\c@part=\count79
\c@section=\count80
\c@subsection=\count81
\c@subsubsection=\count82
\c@paragraph=\count83
\c@subparagraph=\count84
\c@figure=\count85
\c@table=\count86
\abovecaptionskip=\skip41
\belowcaptionskip=\skip42
\bibindent=\dimen102
) (c:/TeXLive/2015/texmf-dist/tex/latex/base/inputenc.sty
Package: inputenc 2015/03/17 v1.2c Input encoding file
\inpenc@prehook=\toks14
\inpenc@posthook=\toks15
(c:/TeXLive/2015/texmf-dist/tex/latex/base/utf8.def
File: utf8.def 2016/02/28 v1.1s UTF-8 support for inputenc
Now handling font encoding OML ...
... no UTF-8 mapping file for font encoding OML
Now handling font encoding T1 ...
... processing UTF-8 mapping file for font encoding T1
(c:/TeXLive/2015/texmf-dist/tex/latex/base/t1enc.dfu
File: t1enc.dfu 2016/02/28 v1.1s UTF-8 support for inputenc
   defining Unicode char U+00A0 (decimal 160)
   defining Unicode char U+00A1 (decimal 161)
   defining Unicode char U+00A3 (decimal 163)
   defining Unicode char U+00AB (decimal 171)
   defining Unicode char U+00AD (decimal 173)
   defining Unicode char U+00BB (decimal 187)
   defining Unicode char U+00BF (decimal 191)
   defining Unicode char U+00C0 (decimal 192)
   defining Unicode char U+00C1 (decimal 193)
   defining Unicode char U+00C2 (decimal 194)
   defining Unicode char U+00C3 (decimal 195)
   defining Unicode char U+00C4 (decimal 196)
   defining Unicode char U+00C5 (decimal 197)
   defining Unicode char U+00C6 (decimal 198)
   defining Unicode char U+00C7 (decimal 199)
```

```
defining Unicode char U+00C8 (decimal 200)
defining Unicode char U+00C9 (decimal 201)
defining Unicode char U+00CA (decimal 202)
defining Unicode char U+00CB (decimal 203)
defining Unicode char U+00CC (decimal 204)
defining Unicode char U+00CD (decimal 205)
defining Unicode char U+00CE (decimal 206)
defining Unicode char U+00CF (decimal 207)
defining Unicode char U+00D0 (decimal 208)
defining Unicode char U+00D1 (decimal 209)
defining Unicode char U+00D2 (decimal 210)
defining Unicode char U+00D3 (decimal 211)
defining Unicode char U+00D4 (decimal 212)
defining Unicode char U+00D5 (decimal 213)
defining Unicode char U+00D6 (decimal 214)
defining Unicode char U+00D8 (decimal 216)
defining Unicode char U+00D9 (decimal 217)
defining Unicode char U+00DA (decimal 218)
defining Unicode char U+00DB (decimal 219)
defining Unicode char U+00DC (decimal 220)
defining Unicode char U+00DD (decimal 221)
defining Unicode char U+00DE (decimal 222)
defining Unicode char U+00DF (decimal 223)
defining Unicode char U+00E0 (decimal 224)
defining Unicode char U+00E1 (decimal 225)
defining Unicode char U+00E2 (decimal 226)
defining Unicode char U+00E3 (decimal 227)
defining Unicode char U+00E4 (decimal 228)
defining Unicode char U+00E5 (decimal 229)
defining Unicode char U+00E6 (decimal 230)
defining Unicode char U+00E7 (decimal 231)
defining Unicode char U+00E8 (decimal 232)
defining Unicode char U+00E9 (decimal 233)
defining Unicode char U+00EA (decimal 234)
defining Unicode char U+00EB (decimal 235)
defining Unicode char U+00EC (decimal 236)
defining Unicode char U+00ED (decimal 237)
defining Unicode char U+00EE (decimal 238)
defining Unicode char U+00EF (decimal 239)
defining Unicode char U+00F0 (decimal 240)
defining Unicode char U+00F1 (decimal 241)
defining Unicode char U+00F2 (decimal 242)
defining Unicode char U+00F3 (decimal 243)
defining Unicode char U+00F4 (decimal 244)
defining Unicode char U+00F5 (decimal 245)
defining Unicode char U+00F6 (decimal 246)
defining Unicode char U+00F8 (decimal 248)
defining Unicode char U+00F9 (decimal 249)
defining Unicode char U+00FA (decimal 250)
defining Unicode char U+00FB (decimal 251)
defining Unicode char U+00FC (decimal 252)
defining Unicode char U+00FD (decimal 253)
defining Unicode char U+00FE (decimal 254)
defining Unicode char U+00FF (decimal 255)
```

```
defining Unicode char U+0100 (decimal 256)
defining Unicode char U+0101 (decimal 257)
defining Unicode char U+0102 (decimal 258)
defining Unicode char U+0103 (decimal 259)
defining Unicode char U+0104 (decimal 260)
defining Unicode char U+0105 (decimal 261)
defining Unicode char U+0106 (decimal 262)
defining Unicode char U+0107 (decimal 263)
defining Unicode char U+0108 (decimal 264)
defining Unicode char U+0109 (decimal 265)
defining Unicode char U+010A (decimal 266)
defining Unicode char U+010B (decimal 267)
defining Unicode char U+010C (decimal 268)
defining Unicode char U+010D (decimal 269)
defining Unicode char U+010E (decimal 270)
defining Unicode char U+010F (decimal 271)
defining Unicode char U+0110 (decimal 272)
defining Unicode char U+0111 (decimal 273)
defining Unicode char U+0112 (decimal 274)
defining Unicode char U+0113 (decimal 275)
defining Unicode char U+0114 (decimal 276)
defining Unicode char U+0115 (decimal 277)
defining Unicode char U+0116 (decimal 278)
defining Unicode char U+0117 (decimal 279)
defining Unicode char U+0118 (decimal 280)
defining Unicode char U+0119 (decimal 281)
defining Unicode char U+011A (decimal 282)
defining Unicode char U+011B (decimal 283)
defining Unicode char U+011C (decimal 284)
defining Unicode char U+011D (decimal 285)
defining Unicode char U+011E (decimal 286)
defining Unicode char U+011F (decimal 287)
defining Unicode char U+0120 (decimal 288)
defining Unicode char U+0121 (decimal 289)
defining Unicode char U+0122 (decimal 290)
defining Unicode char U+0123 (decimal 291)
defining Unicode char U+0124 (decimal 292)
defining Unicode char U+0125 (decimal 293)
defining Unicode char U+0128 (decimal 296)
defining Unicode char U+0129 (decimal 297)
defining Unicode char U+012A (decimal 298)
defining Unicode char U+012B (decimal 299)
defining Unicode char U+012C (decimal 300)
defining Unicode char U+012D (decimal 301)
defining Unicode char U+012E (decimal 302)
defining Unicode char U+012F (decimal 303)
defining Unicode char U+0130 (decimal 304)
defining Unicode char U+0131 (decimal 305)
defining Unicode char U+0132 (decimal 306)
defining Unicode char U+0133 (decimal 307)
defining Unicode char U+0134 (decimal 308)
defining Unicode char U+0135 (decimal 309)
defining Unicode char U+0136 (decimal 310)
defining Unicode char U+0137 (decimal 311)
```

```
defining Unicode char U+0139 (decimal 313)
defining Unicode char U+013A (decimal 314)
defining Unicode char U+013B (decimal 315)
defining Unicode char U+013C (decimal 316)
defining Unicode char U+013D (decimal 317)
defining Unicode char U+013E (decimal 318)
defining Unicode char U+0141 (decimal 321)
defining Unicode char U+0142 (decimal 322)
defining Unicode char U+0143 (decimal 323)
defining Unicode char U+0144 (decimal 324)
defining Unicode char U+0145 (decimal 325)
defining Unicode char U+0146 (decimal 326)
defining Unicode char U+0147 (decimal 327)
defining Unicode char U+0148 (decimal 328)
defining Unicode char U+014A (decimal 330)
defining Unicode char U+014B (decimal 331)
defining Unicode char U+014C (decimal 332)
defining Unicode char U+014D (decimal 333)
defining Unicode char U+014E (decimal 334)
defining Unicode char U+014F (decimal 335)
defining Unicode char U+0150 (decimal 336)
defining Unicode char U+0151 (decimal 337)
defining Unicode char U+0152 (decimal 338)
defining Unicode char U+0153 (decimal 339)
defining Unicode char U+0154 (decimal 340)
defining Unicode char U+0155 (decimal 341)
defining Unicode char U+0156 (decimal 342)
defining Unicode char U+0157 (decimal 343)
defining Unicode char U+0158 (decimal 344)
defining Unicode char U+0159 (decimal 345)
defining Unicode char U+015A (decimal 346)
defining Unicode char U+015B (decimal 347)
defining Unicode char U+015C (decimal 348)
defining Unicode char U+015D (decimal 349)
defining Unicode char U+015E (decimal 350)
defining Unicode char U+015F (decimal 351)
defining Unicode char U+0160 (decimal 352)
defining Unicode char U+0161 (decimal 353)
defining Unicode char U+0162 (decimal 354)
defining Unicode char U+0163 (decimal 355)
defining Unicode char U+0164 (decimal 356)
defining Unicode char U+0165 (decimal 357)
defining Unicode char U+0168 (decimal 360)
defining Unicode char U+0169 (decimal 361)
defining Unicode char U+016A (decimal 362)
defining Unicode char U+016B (decimal 363)
defining Unicode char U+016C (decimal 364)
defining Unicode char U+016D (decimal 365)
defining Unicode char U+016E (decimal 366)
defining Unicode char U+016F (decimal 367)
defining Unicode char U+0170 (decimal 368)
defining Unicode char U+0171 (decimal 369)
defining Unicode char U+0172 (decimal 370)
defining Unicode char U+0173 (decimal 371)
```

```
defining Unicode char U+0174 (decimal 372)
defining Unicode char U+0175 (decimal 373)
defining Unicode char U+0176 (decimal 374)
defining Unicode char U+0177 (decimal 375)
defining Unicode char U+0178 (decimal 376)
defining Unicode char U+0179 (decimal 377)
defining Unicode char U+017A (decimal 378)
defining Unicode char U+017B (decimal 379)
defining Unicode char U+017C (decimal 380)
defining Unicode char U+017D (decimal 381)
defining Unicode char U+017E (decimal 382)
defining Unicode char U+01CD (decimal 461)
defining Unicode char U+01CE (decimal 462)
defining Unicode char U+01CF (decimal 463)
defining Unicode char U+01D0 (decimal 464)
defining Unicode char U+01D1 (decimal 465)
defining Unicode char U+01D2 (decimal 466)
defining Unicode char U+01D3 (decimal 467)
defining Unicode char U+01D4 (decimal 468)
defining Unicode char U+01E2 (decimal 482)
defining Unicode char U+01E3 (decimal 483)
defining Unicode char U+01E6 (decimal 486)
defining Unicode char U+01E7 (decimal 487)
defining Unicode char U+01E8 (decimal 488)
defining Unicode char U+01E9 (decimal 489)
defining Unicode char U+01EA (decimal 490)
defining Unicode char U+01EB (decimal 491)
defining Unicode char U+01F0 (decimal 496)
defining Unicode char U+01F4 (decimal 500)
defining Unicode char U+01F5 (decimal 501)
defining Unicode char U+0218 (decimal 536)
defining Unicode char U+0219 (decimal 537)
defining Unicode char U+021A (decimal 538)
defining Unicode char U+021B (decimal 539)
defining Unicode char U+1E02 (decimal 7682)
defining Unicode char U+1E03 (decimal 7683)
defining Unicode char U+200C (decimal 8204)
defining Unicode char U+2010 (decimal 8208)
defining Unicode char U+2011 (decimal 8209)
defining Unicode char U+2012 (decimal 8210)
defining Unicode char U+2013 (decimal 8211)
defining Unicode char U+2014 (decimal 8212)
defining Unicode char U+2015 (decimal 8213)
defining Unicode char U+2018 (decimal 8216)
defining Unicode char U+2019 (decimal 8217)
defining Unicode char U+201A (decimal 8218)
defining Unicode char U+201C (decimal 8220)
defining Unicode char U+201D (decimal 8221)
defining Unicode char U+201E (decimal 8222)
defining Unicode char U+2030 (decimal 8240)
defining Unicode char U+2031 (decimal 8241)
defining Unicode char U+2039 (decimal 8249)
defining Unicode char U+203A (decimal 8250)
defining Unicode char U+2423 (decimal 9251)
```

```
)
Now handling font encoding OT1 ...
... processing UTF-8 mapping file for font encoding OT1
(c:/TeXLive/2015/texmf-dist/tex/latex/base/ot1enc.dfu
File: ot1enc.dfu 2016/02/28 v1.1s UTF-8 support for inputenc
   defining Unicode char U+00A0 (decimal 160)
   defining Unicode char U+00A1 (decimal 161)
   defining Unicode char U+00A3 (decimal 163)
   defining Unicode char U+00AD (decimal 173)
   defining Unicode char U+00B8 (decimal 184)
   defining Unicode char U+00BF (decimal 191)
   defining Unicode char U+00C5 (decimal 197)
   defining Unicode char U+00C6 (decimal 198)
   defining Unicode char U+00D8 (decimal 216)
   defining Unicode char U+00DF (decimal 223)
   defining Unicode char U+00E6 (decimal 230)
   defining Unicode char U+00EC (decimal 236)
   defining Unicode char U+00ED (decimal 237)
   defining Unicode char U+00EE (decimal 238)
   defining Unicode char U+00EF (decimal 239)
   defining Unicode char U+00F8 (decimal 248)
   defining Unicode char U+0131 (decimal 305)
   defining Unicode char U+0141 (decimal 321)
   defining Unicode char U+0142 (decimal 322)
   defining Unicode char U+0152 (decimal 338)
   defining Unicode char U+0153 (decimal 339)
   defining Unicode char U+0174 (decimal 372)
   defining Unicode char U+0175 (decimal 373)
   defining Unicode char U+0176 (decimal 374)
   defining Unicode char U+0177 (decimal 375)
   defining Unicode char U+0218 (decimal 536)
   defining Unicode char U+0219 (decimal 537)
   defining Unicode char U+021A (decimal 538)
   defining Unicode char U+021B (decimal 539)
   defining Unicode char U+2013 (decimal 8211)
   defining Unicode char U+2014 (decimal 8212)
   defining Unicode char U+2018 (decimal 8216)
   defining Unicode char U+2019 (decimal 8217)
   defining Unicode char U+201C (decimal 8220)
   defining Unicode char U+201D (decimal 8221)
)
Now handling font encoding OMS ...
... processing UTF-8 mapping file for font encoding OMS
(c:/TeXLive/2015/texmf-dist/tex/latex/base/omsenc.dfu
File: omsenc.dfu 2016/02/28 v1.1s UTF-8 support for inputenc
   defining Unicode char U+00A7 (decimal 167)
   defining Unicode char U+00B6 (decimal 182)
   defining Unicode char U+00B7 (decimal 183)
   defining Unicode char U+2020 (decimal 8224)
   defining Unicode char U+2021 (decimal 8225)
   defining Unicode char U+2022 (decimal 8226)
)
Now handling font encoding OMX ...
... no UTF-8 mapping file for font encoding OMX
```

```
Now handling font encoding U ...
... no UTF-8 mapping file for font encoding U
   defining Unicode char U+00A9 (decimal 169)
   defining Unicode char U+00AA (decimal 170)
   defining Unicode char U+00AE (decimal 174)
   defining Unicode char U+00BA (decimal 186)
   defining Unicode char U+02C6 (decimal 710)
   defining Unicode char U+02DC (decimal 732)
   defining Unicode char U+200C (decimal 8204)
   defining Unicode char U+2026 (decimal 8230)
   defining Unicode char U+2122 (decimal 8482)
   defining Unicode char U+2423 (decimal 9251)
)) (c:/TeXLive/2015/texmf-dist/tex/latex/base/fontenc.sty
Package: fontenc 2005/09/27 v1.99g Standard LaTeX package
(c:/TeXLive/2015/texmf-dist/tex/latex/base/t1enc.def
File: t1enc.def 2005/09/27 v1.99g Standard LaTeX file
LaTeX Font Info:    Redeclaring font encoding T1 on input line 48.
)) (c:/TeXLive/2015/texmf-dist/tex/generic/oberdiek/ifpdf.sty
Package: ifpdf 2011/01/30 v2.3 Provides the ifpdf switch (HO)
Package ifpdf Info: pdfTeX in PDF mode is detected.
) (c:/TeXLive/2015/texmf-dist/tex/latex/microtype/microtype.sty
Package: microtype 2013/05/23 v2.5a Micro-typographical refinements (RS)
(c:/TeXLive/2015/texmf-dist/tex/latex/graphics/keyval.sty
Package: keyval 2014/10/28 v1.15 key=value parser (DPC)
\KV@toks@=\toks16
)
\MT@toks=\toks17
\MT@count=\count87
LaTeX Info: Redefining \textls on input line 766.
\MT@outer@kern=\dimen103
LaTeX Info: Redefining \textmicrotypecontext on input line 1285.
\MT@listname@count=\count88
(c:/TeXLive/2015/texmf-dist/tex/latex/microtype/microtype-pdftex.def
File: microtype-pdftex.def 2013/05/23 v2.5a Definitions specific to
pdftex (RS)

LaTeX Info: Redefining \lsstyle on input line 915.
LaTeX Info: Redefining \lslig on input line 915.
\MT@outer@space=\skip43
)
Package microtype Info: Loading configuration file microtype.cfg.
(c:/TeXLive/2015/texmf-dist/tex/latex/microtype/microtype.cfg
File: microtype.cfg 2013/05/23 v2.5a microtype main configuration file
(RS)
)) (c:/TeXLive/2015/texmf-dist/tex/latex/euler/euler.sty
Package: euler 1995/03/05 v2.5
Package: `euler' v2.5 <1995/03/05> (FJ and FMi)
LaTeX Font Info:    Redeclaring symbol font `letters' on input line 35.
LaTeX Font Info:    Encoding `OML' has changed to `U' for symbol font
(Font)              `letters' in the math version `normal' on input line
35.
LaTeX Font Info:    Overwriting symbol font `letters' in version `normal'
(Font)                  OML/cmm/m/it --> U/eur/m/n on input line 35.
LaTeX Font Info:    Encoding `OML' has changed to `U' for symbol font
```

```
(Font)              `letters' in the math version `bold' on input line
35.
LaTeX Font Info:    Overwriting symbol font `letters' in version `bold'
(Font)                  OML/cmm/b/it --> U/eur/m/n on input line 35.
LaTeX Font Info:    Overwriting symbol font `letters' in version `bold'
(Font)                  U/eur/m/n --> U/eur/b/n on input line 36.
LaTeX Font Info:    Redeclaring math symbol \Gamma on input line 47.
LaTeX Font Info:    Redeclaring math symbol \Delta on input line 48.
LaTeX Font Info:    Redeclaring math symbol \Theta on input line 49.
LaTeX Font Info:    Redeclaring math symbol \Lambda on input line 50.
LaTeX Font Info:    Redeclaring math symbol \Xi on input line 51.
LaTeX Font Info:    Redeclaring math symbol \Pi on input line 52.
LaTeX Font Info:    Redeclaring math symbol \Sigma on input line 53.
LaTeX Font Info:    Redeclaring math symbol \Upsilon on input line 54.
LaTeX Font Info:    Redeclaring math symbol \Phi on input line 55.
LaTeX Font Info:    Redeclaring math symbol \Psi on input line 56.
LaTeX Font Info:    Redeclaring math symbol \Omega on input line 57.
\symEulerFraktur=\mathgroup4
LaTeX Font Info:    Overwriting symbol font `EulerFraktur' in version
`bold'
(Font)                  U/euf/m/n --> U/euf/b/n on input line 63.
LaTeX Info: Redefining \oldstylenums on input line 85.
\symEulerScript=\mathgroup5
LaTeX Font Info:    Overwriting symbol font `EulerScript' in version
`bold'
(Font)                  U/eus/m/n --> U/eus/b/n on input line 93.
LaTeX Font Info:    Redeclaring math symbol \aleph on input line 97.
LaTeX Font Info:    Redeclaring math symbol \Re on input line 98.
LaTeX Font Info:    Redeclaring math symbol \Im on input line 99.
LaTeX Font Info:    Redeclaring math delimiter \vert on input line 101.
LaTeX Font Info:    Redeclaring math delimiter \backslash on input line
103.
LaTeX Font Info:    Redeclaring math symbol \neg on input line 106.
LaTeX Font Info:    Redeclaring math symbol \wedge on input line 108.
LaTeX Font Info:    Redeclaring math symbol \vee on input line 110.
LaTeX Font Info:    Redeclaring math symbol \setminus on input line 112.
LaTeX Font Info:    Redeclaring math symbol \sim on input line 113.
LaTeX Font Info:    Redeclaring math symbol \mid on input line 114.
LaTeX Font Info:    Redeclaring math delimiter \arrowvert on input line
116.
LaTeX Font Info:    Redeclaring math symbol \mathsection on input line
117.
\symEulerExtension=\mathgroup6
LaTeX Font Info:    Redeclaring math symbol \coprod on input line 125.
LaTeX Font Info:    Redeclaring math symbol \prod on input line 125.
LaTeX Font Info:    Redeclaring math symbol \sum on input line 125.
LaTeX Font Info:    Redeclaring math symbol \intop on input line 130.
LaTeX Font Info:    Redeclaring math symbol \ointop on input line 131.
LaTeX Font Info:    Redeclaring math symbol \braceld on input line 132.
LaTeX Font Info:    Redeclaring math symbol \bracerd on input line 133.
LaTeX Font Info:    Redeclaring math symbol \bracelu on input line 134.
LaTeX Font Info:    Redeclaring math symbol \braceru on input line 135.
LaTeX Font Info:    Redeclaring math symbol \infty on input line 136.
LaTeX Font Info:    Redeclaring math symbol \nearrow on input line 153.
```

```
LaTeX Font Info:    Redeclaring math symbol \searrow on input line 154.
LaTeX Font Info:    Redeclaring math symbol \nwarrow on input line 155.
LaTeX Font Info:    Redeclaring math symbol \swarrow on input line 156.
LaTeX Font Info:    Redeclaring math symbol \Leftrightarrow on input line
157.
LaTeX Font Info:    Redeclaring math symbol \Leftarrow on input line 158.
LaTeX Font Info:    Redeclaring math symbol \Rightarrow on input line
159.
LaTeX Font Info:    Redeclaring math symbol \leftrightarrow on input line
160.
LaTeX Font Info:    Redeclaring math symbol \leftarrow on input line 161.
LaTeX Font Info:    Redeclaring math symbol \rightarrow on input line
163.
LaTeX Font Info:    Redeclaring math delimiter \uparrow on input line
166.
LaTeX Font Info:    Redeclaring math delimiter \downarrow on input line
168.
LaTeX Font Info:    Redeclaring math delimiter \updownarrow on input line
170.
LaTeX Font Info:    Redeclaring math delimiter \Uparrow on input line
172.
LaTeX Font Info:    Redeclaring math delimiter \Downarrow on input line
174.
LaTeX Font Info:    Redeclaring math delimiter \Updownarrow on input line
176.
LaTeX Font Info:    Redeclaring math symbol \leftharpoonup on input line
177.
LaTeX Font Info:    Redeclaring math symbol \leftharpoondown on input
line 178.

LaTeX Font Info:    Redeclaring math symbol \rightharpoonup on input line
179.
LaTeX Font Info:    Redeclaring math symbol \rightharpoondown on input
line 180
.
LaTeX Font Info:    Redeclaring math delimiter \lbrace on input line 182.
LaTeX Font Info:    Redeclaring math delimiter \rbrace on input line 184.
\symcmmigroup=\mathgroup7
LaTeX Font Info:    Overwriting symbol font `cmmigroup' in version `bold'
(Font)                  OML/cmm/m/it --> OML/cmm/b/it on input line 200.
LaTeX Font Info:    Redeclaring math accent \vec on input line 201.
LaTeX Font Info:    Redeclaring math symbol \triangleleft on input line
202.
LaTeX Font Info:    Redeclaring math symbol \triangleright on input line
203.
LaTeX Font Info:    Redeclaring math symbol \star on input line 204.
LaTeX Font Info:    Redeclaring math symbol \lhook on input line 205.
LaTeX Font Info:    Redeclaring math symbol \rhook on input line 206.
LaTeX Font Info:    Redeclaring math symbol \flat on input line 207.
LaTeX Font Info:    Redeclaring math symbol \natural on input line 208.
LaTeX Font Info:    Redeclaring math symbol \sharp on input line 209.
LaTeX Font Info:    Redeclaring math symbol \smile on input line 210.
LaTeX Font Info:    Redeclaring math symbol \frown on input line 211.
LaTeX Font Info:    Redeclaring math accent \grave on input line 245.
```

```
LaTeX Font Info:    Redeclaring math accent \acute on input line 246.
LaTeX Font Info:    Redeclaring math accent \tilde on input line 247.
LaTeX Font Info:    Redeclaring math accent \ddot on input line 248.
LaTeX Font Info:    Redeclaring math accent \check on input line 249.
LaTeX Font Info:    Redeclaring math accent \breve on input line 250.
LaTeX Font Info:    Redeclaring math accent \bar on input line 251.
LaTeX Font Info:    Redeclaring math accent \dot on input line 252.
LaTeX Font Info:    Redeclaring math accent \hat on input line 254.
) (c:/TeXLive/2015/texmf-dist/tex/latex/merriweather/merriweather.sty
Package: merriweather 2014/01/22 (Bob Tennent) Supports
Merriweather(Sans) font
s for all LaTeX engines.
(c:/TeXLive/2015/texmf-dist/tex/generic/ifxetex/ifxetex.sty
Package: ifxetex 2010/09/12 v0.6 Provides ifxetex conditional
) (c:/TeXLive/2015/texmf-dist/tex/generic/oberdiek/ifluatex.sty
Package: ifluatex 2010/03/01 v1.3 Provides the ifluatex switch (HO)
Package ifluatex Info: LuaTeX not detected.
) (c:/TeXLive/2015/texmf-dist/tex/latex/base/textcomp.sty
Package: textcomp 2005/09/27 v1.99g Standard LaTeX package
Package textcomp Info: Sub-encoding information:
(textcomp)              5 = only ISO-Adobe without \textcurrency
(textcomp)              4 = 5 + \texteuro
(textcomp)              3 = 4 + \textohm
(textcomp)              2 = 3 + \textestimated + \textcurrency
(textcomp)              1 = TS1 - \textcircled - \t
(textcomp)              0 = TS1 (full)
(textcomp)             Font families with sub-encoding setting implement
(textcomp)             only a restricted character set as indicated.
(textcomp)             Family '?' is the default used for unknown fonts.
(textcomp)             See the documentation for details.
Package textcomp Info: Setting ? sub-encoding to TS1/1 on input line 79.
(c:/TeXLive/2015/texmf-dist/tex/latex/base/ts1enc.def
File: ts1enc.def 2001/06/05 v3.0e (jk/car/fm) Standard LaTeX file
Now handling font encoding TS1 ...
... processing UTF-8 mapping file for font encoding TS1
(c:/TeXLive/2015/texmf-dist/tex/latex/base/ts1enc.dfu
File: ts1enc.dfu 2016/02/28 v1.1s UTF-8 support for inputenc
   defining Unicode char U+00A2 (decimal 162)
   defining Unicode char U+00A3 (decimal 163)
   defining Unicode char U+00A4 (decimal 164)
   defining Unicode char U+00A5 (decimal 165)
   defining Unicode char U+00A6 (decimal 166)
   defining Unicode char U+00A7 (decimal 167)
   defining Unicode char U+00A8 (decimal 168)
   defining Unicode char U+00A9 (decimal 169)
   defining Unicode char U+00AA (decimal 170)
   defining Unicode char U+00AC (decimal 172)
   defining Unicode char U+00AE (decimal 174)
   defining Unicode char U+00AF (decimal 175)
   defining Unicode char U+00B0 (decimal 176)
   defining Unicode char U+00B1 (decimal 177)
   defining Unicode char U+00B2 (decimal 178)
   defining Unicode char U+00B3 (decimal 179)
   defining Unicode char U+00B4 (decimal 180)
```

```
    defining Unicode char U+00B5 (decimal 181)
    defining Unicode char U+00B6 (decimal 182)
    defining Unicode char U+00B7 (decimal 183)
    defining Unicode char U+00B9 (decimal 185)
    defining Unicode char U+00BA (decimal 186)
    defining Unicode char U+00BC (decimal 188)
    defining Unicode char U+00BD (decimal 189)
    defining Unicode char U+00BE (decimal 190)
    defining Unicode char U+00D7 (decimal 215)
    defining Unicode char U+00F7 (decimal 247)
    defining Unicode char U+0192 (decimal 402)
    defining Unicode char U+02C7 (decimal 711)
    defining Unicode char U+02D8 (decimal 728)
    defining Unicode char U+02DD (decimal 733)
    defining Unicode char U+0E3F (decimal 3647)
    defining Unicode char U+2016 (decimal 8214)
    defining Unicode char U+2020 (decimal 8224)
    defining Unicode char U+2021 (decimal 8225)
    defining Unicode char U+2022 (decimal 8226)
    defining Unicode char U+2030 (decimal 8240)
    defining Unicode char U+2031 (decimal 8241)
    defining Unicode char U+203B (decimal 8251)
    defining Unicode char U+203D (decimal 8253)
    defining Unicode char U+2044 (decimal 8260)
    defining Unicode char U+204E (decimal 8270)
    defining Unicode char U+2052 (decimal 8274)
    defining Unicode char U+20A1 (decimal 8353)
    defining Unicode char U+20A4 (decimal 8356)
    defining Unicode char U+20A6 (decimal 8358)
    defining Unicode char U+20A9 (decimal 8361)
    defining Unicode char U+20AB (decimal 8363)
    defining Unicode char U+20AC (decimal 8364)
    defining Unicode char U+20B1 (decimal 8369)
    defining Unicode char U+2103 (decimal 8451)
    defining Unicode char U+2116 (decimal 8470)
    defining Unicode char U+2117 (decimal 8471)
    defining Unicode char U+211E (decimal 8478)
    defining Unicode char U+2120 (decimal 8480)
    defining Unicode char U+2122 (decimal 8482)
    defining Unicode char U+2126 (decimal 8486)
    defining Unicode char U+2127 (decimal 8487)
    defining Unicode char U+212E (decimal 8494)
    defining Unicode char U+2190 (decimal 8592)
    defining Unicode char U+2191 (decimal 8593)
    defining Unicode char U+2192 (decimal 8594)
    defining Unicode char U+2193 (decimal 8595)
    defining Unicode char U+2329 (decimal 9001)
    defining Unicode char U+232A (decimal 9002)
    defining Unicode char U+2422 (decimal 9250)
    defining Unicode char U+25E6 (decimal 9702)
    defining Unicode char U+25EF (decimal 9711)
    defining Unicode char U+266A (decimal 9834)
))
LaTeX Info: Redefining \oldstylenums on input line 334.
```

```
Package textcomp Info: Setting cmr sub-encoding to TS1/0 on input line
349.
Package textcomp Info: Setting cmss sub-encoding to TS1/0 on input line
350.
Package textcomp Info: Setting cmtt sub-encoding to TS1/0 on input line
351.
Package textcomp Info: Setting cmvtt sub-encoding to TS1/0 on input line
352.
Package textcomp Info: Setting cmbr sub-encoding to TS1/0 on input line
353.
Package textcomp Info: Setting cmtl sub-encoding to TS1/0 on input line
354.
Package textcomp Info: Setting ccr sub-encoding to TS1/0 on input line
355.
Package textcomp Info: Setting ptm sub-encoding to TS1/4 on input line
356.
Package textcomp Info: Setting pcr sub-encoding to TS1/4 on input line
357.
Package textcomp Info: Setting phv sub-encoding to TS1/4 on input line
358.
Package textcomp Info: Setting ppl sub-encoding to TS1/3 on input line
359.
Package textcomp Info: Setting pag sub-encoding to TS1/4 on input line
360.
Package textcomp Info: Setting pbk sub-encoding to TS1/4 on input line
361.
Package textcomp Info: Setting pnc sub-encoding to TS1/4 on input line
362.
Package textcomp Info: Setting pzc sub-encoding to TS1/4 on input line
363.
Package textcomp Info: Setting bch sub-encoding to TS1/4 on input line
364.
Package textcomp Info: Setting put sub-encoding to TS1/5 on input line
365.
Package textcomp Info: Setting uag sub-encoding to TS1/5 on input line
366.
Package textcomp Info: Setting ugq sub-encoding to TS1/5 on input line
367.
Package textcomp Info: Setting ul8 sub-encoding to TS1/4 on input line
368.
Package textcomp Info: Setting ul9 sub-encoding to TS1/4 on input line
369.
Package textcomp Info: Setting augie sub-encoding to TS1/5 on input line
370.
Package textcomp Info: Setting dayrom sub-encoding to TS1/3 on input line
371.
Package textcomp Info: Setting dayroms sub-encoding to TS1/3 on input
line 372.

Package textcomp Info: Setting pxr sub-encoding to TS1/0 on input line
373.
Package textcomp Info: Setting pxss sub-encoding to TS1/0 on input line
374.
```

```
Package textcomp Info: Setting pxtt sub-encoding to TS1/0 on input line
375.
Package textcomp Info: Setting txr sub-encoding to TS1/0 on input line
376.
Package textcomp Info: Setting txss sub-encoding to TS1/0 on input line
377.
Package textcomp Info: Setting txtt sub-encoding to TS1/0 on input line
378.
Package textcomp Info: Setting lmr sub-encoding to TS1/0 on input line
379.
Package textcomp Info: Setting lmdh sub-encoding to TS1/0 on input line
380.
Package textcomp Info: Setting lmss sub-encoding to TS1/0 on input line
381.
Package textcomp Info: Setting lmssq sub-encoding to TS1/0 on input line
382.
Package textcomp Info: Setting lmvtt sub-encoding to TS1/0 on input line
383.
Package textcomp Info: Setting lmtt sub-encoding to TS1/0 on input line
384.
Package textcomp Info: Setting qhv sub-encoding to TS1/0 on input line
385.
Package textcomp Info: Setting qag sub-encoding to TS1/0 on input line
386.
Package textcomp Info: Setting qbk sub-encoding to TS1/0 on input line
387.
Package textcomp Info: Setting qcr sub-encoding to TS1/0 on input line
388.
Package textcomp Info: Setting qcs sub-encoding to TS1/0 on input line
389.
Package textcomp Info: Setting qpl sub-encoding to TS1/0 on input line
390.
Package textcomp Info: Setting qtm sub-encoding to TS1/0 on input line
391.
Package textcomp Info: Setting qzc sub-encoding to TS1/0 on input line
392.
Package textcomp Info: Setting qhvc sub-encoding to TS1/0 on input line
393.
Package textcomp Info: Setting futs sub-encoding to TS1/4 on input line
394.
Package textcomp Info: Setting futx sub-encoding to TS1/4 on input line
395.
Package textcomp Info: Setting futj sub-encoding to TS1/4 on input line
396.
Package textcomp Info: Setting hlh sub-encoding to TS1/3 on input line
397.
Package textcomp Info: Setting hls sub-encoding to TS1/3 on input line
398.
Package textcomp Info: Setting hlst sub-encoding to TS1/3 on input line
399.
Package textcomp Info: Setting hlct sub-encoding to TS1/5 on input line
400.
Package textcomp Info: Setting hlx sub-encoding to TS1/5 on input line
401.
```

```
Package textcomp Info: Setting hlce sub-encoding to TS1/5 on input line
402.
Package textcomp Info: Setting hlcn sub-encoding to TS1/5 on input line
403.
Package textcomp Info: Setting hlcw sub-encoding to TS1/5 on input line
404.
Package textcomp Info: Setting hlcf sub-encoding to TS1/5 on input line
405.
Package textcomp Info: Setting pplx sub-encoding to TS1/3 on input line
406.
Package textcomp Info: Setting pplj sub-encoding to TS1/3 on input line
407.
Package textcomp Info: Setting ptmx sub-encoding to TS1/4 on input line
408.
Package textcomp Info: Setting ptmj sub-encoding to TS1/4 on input line
409.
) (c:/TeXLive/2015/texmf-dist/tex/latex/xkeyval/xkeyval.sty
Package: xkeyval 2014/12/03 v2.7a package option processing (HA)
(c:/TeXLive/2015/texmf-dist/tex/generic/xkeyval/xkeyval.tex
(c:/TeXLive/2015/te
xmf-dist/tex/generic/xkeyval/xkvutils.tex
\XKV@toks=\toks18
\XKV@tempa@toks=\toks19
)
\XKV@depth=\count89
File: xkeyval.tex 2014/12/03 v2.7a key=value parser (HA)
)) (c:/TeXLive/2015/texmf-dist/tex/latex/base/fontenc.sty
Package: fontenc 2005/09/27 v1.99g Standard LaTeX package
) (c:/TeXLive/2015/texmf-dist/tex/latex/fontaxes/fontaxes.sty
Package: fontaxes 2014/03/23 v1.0d Font selection axes
LaTeX Info: Redefining \upshape on input line 29.
LaTeX Info: Redefining \itshape on input line 31.
LaTeX Info: Redefining \slshape on input line 33.
LaTeX Info: Redefining \scshape on input line 37.
) (c:/TeXLive/2015/texmf-dist/tex/latex/mweights/mweights.sty
Package: mweights 2013/07/21 (Bob Tennent) Support package for multiple-
weight
font packages.
LaTeX Info: Redefining \bfseries on input line 22.
LaTeX Info: Redefining \mdseries on input line 29.
LaTeX Info: Redefining \rmfamily on input line 36.
LaTeX Info: Redefining \sffamily on input line 53.
LaTeX Info: Redefining \ttfamily on input line 70.
)) (c:/TeXLive/2015/texmf-dist/tex/latex/mathastext/mathastext.sty
Package: mathastext 2016/04/02 v1.3m Use the text font in math mode (jfB)
\mst@exists@muskip=\muskip10
\mst@forall@muskip=\muskip11
\mst@prime@muskip=\muskip12
\mst@do@nonletters=\toks20
\mst@do@easynonletters=\toks21
\mst@do@az=\toks22
\mst@do@AZ=\toks23
\symmtoperatorfont=\mathgroup8
\symmtletterfont=\mathgroup9
```

```
LaTeX Font Info:    Overwriting math alphabet `\mathnormalbold' in
version `nor
mal'
(Font)                  T1/Merriweather-OsF/bx/it --> T1/Merriweather-
OsF/bx/it
 on input line 1007.
LaTeX Font Info:    Overwriting math alphabet `\mathnormalbold' in
version `bol
d'
(Font)                  T1/Merriweather-OsF/bx/it --> T1/Merriweather-
OsF/bx/it
 on input line 1007.
LaTeX Font Info:    Overwriting symbol font `mtletterfont' in version
`normal'
(Font)                  T1/Merriweather-OsF/m/it --> T1/Merriweather-
OsF/m/it o
n input line 1007.
LaTeX Font Info:    Overwriting symbol font `mtletterfont' in version
`bold'
(Font)                  T1/Merriweather-OsF/m/it --> T1/Merriweather-
OsF/bx/it
on input line 1007.
LaTeX Font Info:    Overwriting symbol font `mtoperatorfont' in version
`normal
'
(Font)                  T1/Merriweather-OsF/m/n --> T1/Merriweather-
OsF/m/n on
input line 1007.
LaTeX Font Info:    Overwriting symbol font `mtoperatorfont' in version
`bold'
(Font)                  T1/Merriweather-OsF/m/n --> T1/Merriweather-
OsF/bx/n on
 input line 1007.
LaTeX Font Info:    Overwriting math alphabet `\Mathbf' in version
`normal'
(Font)                  T1/Merriweather-OsF/bx/n --> T1/Merriweather-
OsF/bx/n o
n input line 1007.
LaTeX Font Info:    Overwriting math alphabet `\Mathbf' in version `bold'
(Font)                  T1/Merriweather-OsF/bx/n --> T1/Merriweather-
OsF/bx/n o
n input line 1007.
LaTeX Font Info:    Overwriting math alphabet `\Mathit' in version
`normal'
(Font)                  T1/Merriweather-OsF/m/it --> T1/Merriweather-
OsF/m/it o
n input line 1007.
LaTeX Font Info:    Overwriting math alphabet `\Mathit' in version `bold'
(Font)                  T1/Merriweather-OsF/m/it --> T1/Merriweather-
OsF/bx/it
on input line 1007.
LaTeX Font Info:    Overwriting math alphabet `\Mathsf' in version
`normal'
```

```
(Font)                  T1/MerriweatherSans-TLF/m/n -->
T1/MerriweatherSans-TLF
/m/n on input line 1007.
LaTeX Font Info:    Overwriting math alphabet `\Mathsf' in version `bold'
(Font)                  T1/MerriweatherSans-TLF/m/n -->
T1/MerriweatherSans-TLF
/bx/n on input line 1007.
LaTeX Font Info:    Overwriting math alphabet `\Mathtt' in version
`normal'
(Font)                  T1/lmtt/m/n --> T1/lmtt/m/n on input line 1007.
LaTeX Font Info:    Overwriting math alphabet `\Mathtt' in version `bold'
(Font)                  T1/lmtt/m/n --> T1/lmtt/bx/n on input line 1007.
** Latin letters in the normal (resp. bold) math versions are now
** set up to use the fonts T1/Merriweather-OsF/m(bx)/it
** Other characters (digits, ...) and \log-like names will be
** typeset with the n shape.
** ! and ?
** punctuation: , . : ; and \colon
LaTeX Info: Redefining \relbar on input line 1153.
** minus as endash
** \hbar
** + and =
LaTeX Info: Redefining \Relbar on input line 1255.
** adding = ; and + to \nfss@catcodes
** parentheses ( ) [ ] and slash /
** alldelims: < > \backslash \setminus | \vert \mid \{ and \}
LaTeX Font Info:    Redeclaring math delimiter \backslash on input line
1301.
LaTeX Font Info:    Redeclaring math symbol \setminus on input line 1313.
LaTeX Info: Redefining \models on input line 1322.
** \# \mathdollar \% \&
** \i and \j
LaTeX Info: Redefining \i on input line 1893.
LaTeX Info: Redefining \j on input line 1894.
** \HUGE has been (re)-defined.
** mathastext has declared larger sizes for subscripts.
** To keep LaTeX defaults, use option `defaultmathsizes'.
) (c:/TeXLive/2015/texmf-dist/tex/latex/relsize/relsize.sty
Package: relsize 2013/03/29 ver 4.1
) (c:/TeXLive/2015/texmf-dist/tex/latex/ms/ragged2e.sty
Package: ragged2e 2009/05/21 v2.1 ragged2e Package (MS)
(c:/TeXLive/2015/texmf-dist/tex/latex/ms/everysel.sty
Package: everysel 2011/10/28 v1.2 EverySelectfont Package (MS)
)
\CenteringLeftskip=\skip44
\RaggedLeftLeftskip=\skip45
\RaggedRightLeftskip=\skip46
\CenteringRightskip=\skip47
\RaggedLeftRightskip=\skip48
\RaggedRightRightskip=\skip49
\CenteringParfillskip=\skip50
\RaggedLeftParfillskip=\skip51
\RaggedRightParfillskip=\skip52
\JustifyingParfillskip=\skip53
```

```
\CenteringParindent=\skip54
\RaggedLeftParindent=\skip55
\RaggedRightParindent=\skip56
\JustifyingParindent=\skip57
) (c:/TeXLive/2015/texmf-dist/tex/latex/xcolor/xcolor.sty
Package: xcolor 2007/01/21 v2.11 LaTeX color extensions (UK)
(c:/TeXLive/2015/texmf-dist/tex/latex/latexconfig/color.cfg
File: color.cfg 2007/01/18 v1.5 color configuration of teTeX/TeXLive
)
Package xcolor Info: Driver file: pdftex.def on input line 225.
(c:/TeXLive/2015/texmf-dist/tex/latex/pdftex-def/pdftex.def
File: pdftex.def 2011/05/27 v0.06d Graphics/color for pdfTeX
(c:/TeXLive/2015/texmf-dist/tex/generic/oberdiek/infwarerr.sty
Package: infwarerr 2010/04/08 v1.3 Providing info/warning/error messages
(HO)
) (c:/TeXLive/2015/texmf-dist/tex/generic/oberdiek/ltxcmds.sty
Package: ltxcmds 2011/11/09 v1.22 LaTeX kernel commands for general use
(HO)
)
\Gread@gobject=\count90
)
Package xcolor Info: Model `cmy' substituted by `cmy0' on input line
1337.
Package xcolor Info: Model `hsb' substituted by `rgb' on input line 1341.
Package xcolor Info: Model `RGB' extended on input line 1353.
Package xcolor Info: Model `HTML' substituted by `rgb' on input line
1355.
Package xcolor Info: Model `Hsb' substituted by `hsb' on input line 1356.
Package xcolor Info: Model `tHsb' substituted by `hsb' on input line
1357.
Package xcolor Info: Model `HSB' substituted by `hsb' on input line 1358.
Package xcolor Info: Model `Gray' substituted by `gray' on input line
1359.
Package xcolor Info: Model `wave' substituted by `hsb' on input line
1360.
) (c:/TeXLive/2015/texmf-dist/tex/latex/colortbl/colortbl.sty
Package: colortbl 2012/02/13 v1.0a Color table columns (DPC)
(c:/TeXLive/2015/texmf-dist/tex/latex/tools/array.sty
Package: array 2014/10/28 v2.4c Tabular extension package (FMi)
\col@sep=\dimen104
\extrarowheight=\dimen105
\NC@list=\toks24
\extratabsurround=\skip58
\backup@length=\skip59
)
\everycr=\toks25
\minrowclearance=\skip60
) (c:/TeXLive/2015/texmf-dist/tex/latex/graphics/graphicx.sty
Package: graphicx 2014/10/28 v1.0g Enhanced LaTeX Graphics (DPC,SPQR)
(c:/TeXLive/2015/texmf-dist/tex/latex/graphics/graphics.sty
Package: graphics 2016/01/03 v1.0q Standard LaTeX Graphics (DPC,SPQR)
(c:/TeXLive/2015/texmf-dist/tex/latex/graphics/trig.sty
Package: trig 2016/01/03 v1.10 sin cos tan (DPC)
) (c:/TeXLive/2015/texmf-dist/tex/latex/latexconfig/graphics.cfg
```

```
File: graphics.cfg 2010/04/23 v1.9 graphics configuration of TeX Live
)
Package graphics Info: Driver file: pdftex.def on input line 95.
)
\Gin@req@height=\dimen106
\Gin@req@width=\dimen107
) (c:/TeXLive/2015/texmf-dist/tex/latex/etoolbox/etoolbox.sty
Package: etoolbox 2015/08/02 v2.2a e-TeX tools for LaTeX (JAW)
\etb@tempcnta=\count91
) (c:/TeXLive/2015/texmf-dist/tex/latex/xpatch/xpatch.sty
(c:/TeXLive/2015/texm
f-dist/tex/latex/l3kernel/expl3.sty
Package: expl3 2016/03/28 v6468 L3 programming layer (loader)
(c:/TeXLive/2015/texmf-dist/tex/latex/l3kernel/expl3-code.tex
Package: expl3 2016/03/28 v6468 L3 programming layer (code)
L3 Module: l3bootstrap 2016/02/12 v6412 L3 Bootstrap code
L3 Module: l3names 2016/03/11 v6433 L3 Namespace for primitives
L3 Module: l3basics 2015/11/22 v6315 L3 Basic definitions
L3 Module: l3expan 2015/09/10 v5983 L3 Argument expansion
L3 Module: l3tl 2016/03/26 v6465 L3 Token lists
L3 Module: l3str 2016/03/24 v6441 L3 Strings
L3 Module: l3seq 2015/08/05 v5777 L3 Sequences and stacks
L3 Module: l3int 2016/03/24 v6441 L3 Integers
\c_max_int=\count92
\l_tmpa_int=\count93
\l_tmpb_int=\count94
\g_tmpa_int=\count95
\g_tmpb_int=\count96
L3 Module: l3quark 2015/08/17 v5855 L3 Quarks
L3 Module: l3prg 2015/11/01 v6216 L3 Control structures
\g__prg_map_int=\count97
L3 Module: l3clist 2015/09/02 v5901 L3 Comma separated lists
L3 Module: l3token 2016/03/26 v6465 L3 Experimental token manipulation
L3 Module: l3prop 2016/01/05 v6366 L3 Property lists
L3 Module: l3msg 2016/03/26 v6464 L3 Messages
L3 Module: l3file 2016/03/25 v6458 L3 File and I/O operations
\l_iow_line_count_int=\count98
\l__iow_target_count_int=\count99
\l__iow_current_line_int=\count100
\l__iow_current_word_int=\count101
\l__iow_current_indentation_int=\count102
L3 Module: l3skip 2016/01/05 v6366 L3 Dimensions and skips
\c_zero_dim=\dimen108
\c_max_dim=\dimen109
\l_tmpa_dim=\dimen110
\l_tmpb_dim=\dimen111
\g_tmpa_dim=\dimen112
\g_tmpb_dim=\dimen113
\c_zero_skip=\skip61
\c_max_skip=\skip62
\l_tmpa_skip=\skip63
\l_tmpb_skip=\skip64
\g_tmpa_skip=\skip65
\g_tmpb_skip=\skip66
```

```
\c_zero_muskip=\muskip13
\c_max_muskip=\muskip14
\l_tmpa_muskip=\muskip15
\l_tmpb_muskip=\muskip16
\g_tmpa_muskip=\muskip17
\g_tmpb_muskip=\muskip18
L3 Module: l3keys 2015/11/17 v6284 L3 Key-value interfaces
\g__keyval_level_int=\count103
\l_keys_choice_int=\count104
L3 Module: l3fp 2016/03/26 v6465 L3 Floating points
\c__fp_leading_shift_int=\count105
\c__fp_middle_shift_int=\count106
\c__fp_trailing_shift_int=\count107
\c__fp_big_leading_shift_int=\count108
\c__fp_big_middle_shift_int=\count109
\c__fp_big_trailing_shift_int=\count110
\c__fp_Bigg_leading_shift_int=\count111
\c__fp_Bigg_middle_shift_int=\count112
\c__fp_Bigg_trailing_shift_int=\count113
L3 Module: l3box 2015/08/09 v5822 L3 Experimental boxes
\c_empty_box=\box26
\l_tmpa_box=\box27
\l_tmpb_box=\box28
\g_tmpa_box=\box29
\g_tmpb_box=\box30
L3 Module: l3coffins 2016/03/24 v6440 L3 Coffin code layer
\l__coffin_internal_box=\box31
\l__coffin_internal_dim=\dimen114
\l__coffin_offset_x_dim=\dimen115
\l__coffin_offset_y_dim=\dimen116
\l__coffin_x_dim=\dimen117
\l__coffin_y_dim=\dimen118
\l__coffin_x_prime_dim=\dimen119
\l__coffin_y_prime_dim=\dimen120
\c_empty_coffin=\box32
\l__coffin_aligned_coffin=\box33
\l__coffin_aligned_internal_coffin=\box34
\l_tmpa_coffin=\box35
\l_tmpb_coffin=\box36
\l__coffin_display_coffin=\box37
\l__coffin_display_coord_coffin=\box38
\l__coffin_display_pole_coffin=\box39
\l__coffin_display_offset_dim=\dimen121
\l__coffin_display_x_dim=\dimen122
\l__coffin_display_y_dim=\dimen123
L3 Module: l3color 2014/08/23 v5354 L3 Experimental color support
L3 Module: l3sys 2015/09/25 v6087 L3 Experimental system/runtime
functions
L3 Module: l3candidates 2016/03/25 v6456 L3 Experimental additions to
l3kernel
\l__box_top_dim=\dimen124
\l__box_bottom_dim=\dimen125
\l__box_left_dim=\dimen126
\l__box_right_dim=\dimen127
```

```
\l__box_top_new_dim=\dimen128
\l__box_bottom_new_dim=\dimen129
\l__box_left_new_dim=\dimen130
\l__box_right_new_dim=\dimen131
\l__box_internal_box=\box40
\l__coffin_bounding_shift_dim=\dimen132
\l__coffin_left_corner_dim=\dimen133
\l__coffin_right_corner_dim=\dimen134
\l__coffin_bottom_corner_dim=\dimen135
\l__coffin_top_corner_dim=\dimen136
\l__coffin_scaled_total_height_dim=\dimen137
\l__coffin_scaled_width_dim=\dimen138
L3 Module: l3luatex 2016/03/26 v6465 L3 Experimental LuaTeX-specific
functions
) (c:/TeXLive/2015/texmf-dist/tex/latex/l3kernel/l3pdfmode.def
File: l3pdfmode.def 2016/03/26 v6465 L3 Experimental driver: PDF mode
\l__driver_color_stack_int=\count114
))
Package: xpatch 2012/10/02 v0.3 Extending etoolbox patching commands
(c:/TeXLive/2015/texmf-dist/tex/latex/l3packages/xparse/xparse.sty
Package: xparse 2016/03/28 v6468 L3 Experimental document command parser
\l__xparse_current_arg_int=\count115
\l__xparse_m_args_int=\count116
\l__xparse_mandatory_args_int=\count117
\l__xparse_processor_int=\count118
\l__xparse_v_nesting_int=\count119
)
.................................................
. LaTeX info: "xparse/define-command"
.
. Defining command \xpatchcmd with sig. '' on line 125.
.................................................
.................................................
. LaTeX info: "xparse/define-command"
.
. Defining command \xpretocmd with sig. '' on line 126.
.................................................
.................................................
. LaTeX info: "xparse/define-command"
.
. Defining command \xapptocmd with sig. '' on line 127.
.................................................
.................................................
. LaTeX info: "xparse/define-command"
.
. Defining command \xshowcmd with sig. '' on line 128.
.................................................
.................................................
. LaTeX info: "xparse/define-command"
.
. Defining command \xpatchbibmacro with sig. 'm' on line 131.
.................................................
.................................................
. LaTeX info: "xparse/define-command"
```

```
.
. Defining command \xpretobibmacro with sig. 'm' on line 133.
.................................................
.................................................
. LaTeX info: "xparse/define-command"
.
. Defining command \xapptobibmacro with sig. 'm' on line 135.
.................................................
.................................................
. LaTeX info: "xparse/define-command"
.
. Defining command \xshowbibmacro with sig. 'm' on line 137.
.................................................
.................................................
. LaTeX info: "xparse/define-command"
.
. Defining command \xpatchfieldformat with sig. 'O{*}m' on line 139.
.................................................
.................................................
. LaTeX info: "xparse/define-command"
.
. Defining command \xpretofieldformat with sig. 'O{*}m' on line 141.
.................................................
.................................................
. LaTeX info: "xparse/define-command"
.
. Defining command \xapptofieldformat with sig. 'O{*}m' on line 143.
.................................................
.................................................
. LaTeX info: "xparse/define-command"
.
. Defining command \xshowfieldformat with sig. 'O{*}m' on line 145.
.................................................
.................................................
. LaTeX info: "xparse/define-command"
.
. Defining command \xpatchnameformat with sig. 'O{*}m' on line 147.
.................................................
.................................................
. LaTeX info: "xparse/define-command"
.
. Defining command \xpretonameformat with sig. 'O{*}m' on line 149.
.................................................
.................................................
. LaTeX info: "xparse/define-command"
.
. Defining command \xapptonameformat with sig. 'O{*}m' on line 151.
.................................................
.................................................
. LaTeX info: "xparse/define-command"
.
. Defining command \xshownameformat with sig. 'O{*}m' on line 153.
.................................................
.................................................
```

```
. LaTeX info: "xparse/define-command"
.
. Defining command \xpatchlistformat with sig. 'O{*}m' on line 155.
.................................................
.................................................
. LaTeX info: "xparse/define-command"
.
. Defining command \xpretolistformat with sig. 'O{*}m' on line 157.
.................................................
.................................................
. LaTeX info: "xparse/define-command"
.
. Defining command \xapptolistformat with sig. 'O{*}m' on line 159.
.................................................
.................................................
. LaTeX info: "xparse/define-command"
.
. Defining command \xshowlistformat with sig. 'O{*}m' on line 161.
.................................................
.................................................
. LaTeX info: "xparse/define-command"
.
. Defining command \xpatchindexfieldformat with sig. 'O{*}m' on line 163.
.................................................
.................................................
. LaTeX info: "xparse/define-command"
.
. Defining command \xpretoindexfieldformat with sig. 'O{*}m' on line 165.
.................................................
.................................................
. LaTeX info: "xparse/define-command"
.
. Defining command \xapptoindexfieldformat with sig. 'O{*}m' on line 167.
.................................................
.................................................
. LaTeX info: "xparse/define-command"
.
. Defining command \xshowindexfieldformat with sig. 'O{*}m' on line 169.
.................................................
.................................................
. LaTeX info: "xparse/define-command"
.
. Defining command \xpatchindexnameformat with sig. 'O{*}m' on line 171.
.................................................
.................................................
. LaTeX info: "xparse/define-command"
.
. Defining command \xpretoindexnameformat with sig. 'O{*}m' on line 173.
.................................................
.................................................
. LaTeX info: "xparse/define-command"
.
. Defining command \xapptoindexnameformat with sig. 'O{*}m' on line 175.
.................................................
```

```
.................................................
. LaTeX info: "xparse/define-command"
.
. Defining command \xshowindexnameformat with sig. 'O{*}m' on line 177.
.................................................
.................................................
. LaTeX info: "xparse/define-command"
.
. Defining command \xpatchindexlistformat with sig. 'O{*}m' on line 179.
.................................................
.................................................
. LaTeX info: "xparse/define-command"
.
. Defining command \xpretoindexlistformat with sig. 'O{*}m' on line 181.
.................................................
.................................................
. LaTeX info: "xparse/define-command"
.
. Defining command \xappindextolistformat with sig. 'O{*}m' on line 183.
.................................................
.................................................
. LaTeX info: "xparse/define-command"
.
. Defining command \xshowindexlistformat with sig. 'O{*}m' on line 185.
.................................................
.................................................
. LaTeX info: "xparse/define-command"
.
. Defining command \xpatchbibdriver with sig. 'm' on line 187.
.................................................
.................................................
. LaTeX info: "xparse/define-command"
.
. Defining command \xpretobibdriver with sig. 'm' on line 189.
.................................................
.................................................
. LaTeX info: "xparse/define-command"
.
. Defining command \xapptobibdriver with sig. 'm' on line 191.
.................................................
.................................................
. LaTeX info: "xparse/define-command"
.
. Defining command \xshowbibdriver with sig. 'm' on line 193.
.................................................
) (c:/TeXLive/2015/texmf-dist/tex/latex/environ/environ.sty
Package: environ 2014/05/04 v0.3 A new way to define environments
(c:/TeXLive/2015/texmf-dist/tex/latex/trimspaces/trimspaces.sty
Package: trimspaces 2009/09/17 v1.1 Trim spaces around a token list
)
\@envbody=\toks26
) (c:/TeXLive/2015/texmf-dist/tex/latex/lastpage/lastpage.sty
Package: lastpage 2015/03/29 v1.2m Refers to last page's name (HMM; JPG)
) (c:/TeXLive/2015/texmf-dist/tex/latex/rotating/rotating.sty
```

```
Package: rotating 2009/03/28 v2.16a rotated objects in LaTeX
(c:/TeXLive/2015/texmf-dist/tex/latex/base/ifthen.sty
Package: ifthen 2014/09/29 v1.1c Standard LaTeX ifthen package (DPC)
)
\c@r@tfl@t=\count120
\rotFPtop=\skip67
\rotFPbot=\skip68
\rot@float@box=\box41
\rot@mess@toks=\toks27
) (c:/TeXLive/2015/texmf-dist/tex/latex/graphics/lscape.sty
Package: lscape 2000/10/22 v3.01 Landscape Pages (DPC)
) (c:/TeXLive/2015/texmf-dist/tex/latex/tools/afterpage.sty
Package: afterpage 2014/10/28 v1.08 After-Page Package (DPC)
\AP@output=\toks28
\AP@partial=\box42
\AP@footins=\box43
) (c:/TeXLive/2015/texmf-dist/tex/latex/textpos/textpos.sty
Package: textpos 2014/01/03 v1.7j
Package: textpos 2014/01/03 1.7j, absolute positioning of text on the
page
(c:/TeXLive/2015/texmf-dist/tex/latex/ms/everyshi.sty
Package: everyshi 2001/05/15 v3.00 EveryShipout Package (MS)
)
\TP@textbox=\box44
\TP@holdbox=\box45
\TPHorizModule=\dimen139
\TPVertModule=\dimen140
\TP@margin=\dimen141
\TP@absmargin=\dimen142
Grid set 16 x 16 = 37.34424pt x 52.81541pt
\TPboxrulesize=\dimen143
\TP@ox=\dimen144
\TP@oy=\dimen145
\TP@tbargs=\toks29
\TP@prevdepth=\dimen146
TextBlockOrigin set to 0pt x 0pt
) (c:/TeXLive/2015/texmf-dist/tex/latex/url/url.sty
\Urlmuskip=\muskip19
Package: url 2013/09/16  ver 3.4  Verb mode for urls, etc.
) (c:/TeXLive/2015/texmf-dist/tex/latex/caption/newfloat.sty
Package: newfloat 2015/09/17 v1.1-109 Defining new floating environments
(AR)
) (c:/TeXLive/2015/texmf-dist/tex/latex/mdframed/mdframed.sty
Package: mdframed 2013/07/01 1.9b: mdframed
(c:/TeXLive/2015/texmf-dist/tex/latex/oberdiek/kvoptions.sty
Package: kvoptions 2011/06/30 v3.11 Key value format for package options
(HO)
(c:/TeXLive/2015/texmf-dist/tex/generic/oberdiek/kvsetkeys.sty
Package: kvsetkeys 2012/04/25 v1.16 Key value parser (HO)
(c:/TeXLive/2015/texmf-dist/tex/generic/oberdiek/etexcmds.sty
Package: etexcmds 2011/02/16 v1.5 Avoid name clashes with e-TeX commands
(HO)
Package etexcmds Info: Could not find \expanded.
```

```
(etexcmds)              That can mean that you are not using pdfTeX 1.50
or
(etexcmds)              that some package has redefined \expanded.
(etexcmds)              In the latter case, load this package earlier.
))) (c:/TeXLive/2015/texmf-dist/tex/latex/oberdiek/zref-abspage.sty
Package: zref-abspage 2012/04/04 v2.24 Module abspage for zref (HO)
(c:/TeXLive/2015/texmf-dist/tex/latex/oberdiek/zref-base.sty
Package: zref-base 2012/04/04 v2.24 Module base for zref (HO)
(c:/TeXLive/2015/texmf-dist/tex/generic/oberdiek/kvdefinekeys.sty
Package: kvdefinekeys 2011/04/07 v1.3 Define keys (HO)
) (c:/TeXLive/2015/texmf-dist/tex/generic/oberdiek/pdftexcmds.sty
Package: pdftexcmds 2011/11/29 v0.20 Utility functions of pdfTeX for
LuaTeX (HO
)
Package pdftexcmds Info: LuaTeX not detected.
Package pdftexcmds Info: \pdf@primitive is available.
Package pdftexcmds Info: \pdf@ifprimitive is available.
Package pdftexcmds Info: \pdfdraftmode found.
) (c:/TeXLive/2015/texmf-dist/tex/latex/oberdiek/auxhook.sty
Package: auxhook 2011/03/04 v1.3 Hooks for auxiliary files (HO)
)
Package zref Info: New property list: main on input line 759.
Package zref Info: New property: default on input line 760.
Package zref Info: New property: page on input line 761.
) (c:/TeXLive/2015/texmf-dist/tex/generic/oberdiek/atbegshi.sty
Package: atbegshi 2011/10/05 v1.16 At begin shipout hook (HO)
)
\c@abspage=\count121
Package zref Info: New property: abspage on input line 62.
) (c:/TeXLive/2015/texmf-dist/tex/latex/needspace/needspace.sty
Package: needspace 2010/09/12 v1.3d reserve vertical space
)
\mdf@templength=\skip69
\c@mdf@globalstyle@cnt=\count122
\mdf@skipabove@length=\skip70
\mdf@skipbelow@length=\skip71
\mdf@leftmargin@length=\skip72
\mdf@rightmargin@length=\skip73
\mdf@innerleftmargin@length=\skip74
\mdf@innerrightmargin@length=\skip75
\mdf@innertopmargin@length=\skip76
\mdf@innerbottommargin@length=\skip77
\mdf@splittopskip@length=\skip78
\mdf@splitbottomskip@length=\skip79
\mdf@outermargin@length=\skip80
\mdf@innermargin@length=\skip81
\mdf@linewidth@length=\skip82
\mdf@innerlinewidth@length=\skip83
\mdf@middlelinewidth@length=\skip84
\mdf@outerlinewidth@length=\skip85
\mdf@roundcorner@length=\skip86
\mdf@footenotedistance@length=\skip87
\mdf@userdefinedwidth@length=\skip88
\mdf@needspace@length=\skip89
```

```
\mdf@frametitleaboveskip@length=\skip90
\mdf@frametitlebelowskip@length=\skip91
\mdf@frametitlerulewidth@length=\skip92
\mdf@frametitleleftmargin@length=\skip93
\mdf@frametitlerightmargin@length=\skip94
\mdf@shadowsize@length=\skip95
\mdf@extratopheight@length=\skip96
\mdf@subtitleabovelinewidth@length=\skip97
\mdf@subtitlebelowlinewidth@length=\skip98
\mdf@subtitleaboveskip@length=\skip99
\mdf@subtitlebelowskip@length=\skip100
\mdf@subtitleinneraboveskip@length=\skip101
\mdf@subtitleinnerbelowskip@length=\skip102
\mdf@subsubtitleabovelinewidth@length=\skip103
\mdf@subsubtitlebelowlinewidth@length=\skip104
\mdf@subsubtitleaboveskip@length=\skip105
\mdf@subsubtitlebelowskip@length=\skip106
\mdf@subsubtitleinneraboveskip@length=\skip107
\mdf@subsubtitleinnerbelowskip@length=\skip108
(c:/TeXLive/2015/texmf-dist/tex/latex/mdframed/md-frame-0.mdf
File: md-frame-0.mdf 2013/07/01\ 1.9b: md-frame-0
)
\mdf@frametitlebox=\box46
\mdf@footnotebox=\box47
\mdf@splitbox@one=\box48
\mdf@splitbox@two=\box49
\mdf@splitbox@save=\box50
\mdfsplitboxwidth=\skip109
\mdfsplitboxtotalwidth=\skip110
\mdfsplitboxheight=\skip111
\mdfsplitboxdepth=\skip112
\mdfsplitboxtotalheight=\skip113
\mdfframetitleboxwidth=\skip114
\mdfframetitleboxtotalwidth=\skip115
\mdfframetitleboxheight=\skip116
\mdfframetitleboxdepth=\skip117
\mdfframetitleboxtotalheight=\skip118
\mdffootnoteboxwidth=\skip119
\mdffootnoteboxtotalwidth=\skip120
\mdffootnoteboxheight=\skip121
\mdffootnoteboxdepth=\skip122
\mdffootnoteboxtotalheight=\skip123
\mdftotallinewidth=\skip124
\mdfboundingboxwidth=\skip125
\mdfboundingboxtotalwidth=\skip126
\mdfboundingboxheight=\skip127
\mdfboundingboxdepth=\skip128
\mdfboundingboxtotalheight=\skip129
\mdf@freevspace@length=\skip130
\mdf@horizontalwidthofbox@length=\skip131
\mdf@verticalmarginwhole@length=\skip132
\mdf@horizontalspaceofbox=\skip133
\mdfsubtitleheight=\skip134
\mdfsubsubtitleheight=\skip135
```

```
\c@mdfcountframes=\count123

****** mdframed patching \endmdf@trivlist

****** -- success******

.................................................
. LaTeX info: "xparse/define-command"
.
. Defining command \newmdtheoremenv with sig. 'O{} m o m o ' on line 601.
.................................................
.................................................
. LaTeX info: "xparse/define-command"
.
. Defining command \mdtheorem with sig. ' O{} m o m o ' on line 701.
.................................................
\mdf@envdepth=\count124
\c@mdf@env@i=\count125
\c@mdf@env@ii=\count126
\c@mdf@zref@counter=\count127
Package zref Info: New property: mdf@pagevalue on input line 895.
) (c:/TeXLive/2015/texmf-dist/tex/latex/titlesec/titlesec.sty
Package: titlesec 2016/03/21 v2.10.2 Sectioning titles
\ttl@box=\box51
\beforetitleunit=\skip136
\aftertitleunit=\skip137
\ttl@plus=\dimen147
\ttl@minus=\dimen148
\ttl@toksa=\toks30
\titlewidth=\dimen149
\titlewidthlast=\dimen150
\titlewidthfirst=\dimen151
) (c:/TeXLive/2015/texmf-dist/tex/latex/koma-script/scrextend.sty
Package: scrextend 2015/10/03 v3.19a KOMA-Script package (extend other
classes
with features of KOMA-Script classes)
(c:/TeXLive/2015/texmf-dist/tex/latex/koma-script/scrkbase.sty
Package: scrkbase 2015/10/03 v3.19a KOMA-Script package (KOMA-Script-
dependent
basics and keyval usage)
(c:/TeXLive/2015/texmf-dist/tex/latex/koma-script/scrbase.sty
Package: scrbase 2015/10/03 v3.19a KOMA-Script package (KOMA-Script-
independent
 basics and keyval usage)
(c:/TeXLive/2015/texmf-dist/tex/latex/koma-script/scrlfile.sty
Package: scrlfile 2015/10/03 v3.19a KOMA-Script package (loading files)
Package scrlfile, 2015/10/03 v3.19a KOMA-Script package (loading files)
                  Copyright (C) Markus Kohm

)))
LaTeX Info: Redefining \textsubscript on input line 1277.
Package scrextend Info: unexpected definition of `\@makefnmark'.
(scrextend)             Trying to patch it on input line 1391.
Package scrextend Info: patch seems to be successfull on input line 1391.
```

```
)

LaTeX Font Warning: Font shape `T1/cmr/m/n' in size <7.5> not available
(Font)              size <7> substituted on input line 65.

(c:/TeXLive/2015/texmf-dist/tex/latex/tools/calc.sty
Package: calc 2014/10/28 v4.3 Infix arithmetic (KKT,FJ)
\calc@Acount=\count128
\calc@Bcount=\count129
\calc@Adimen=\dimen152
\calc@Bdimen=\dimen153
\calc@Askip=\skip138
\calc@Bskip=\skip139
LaTeX Info: Redefining \setlength on input line 80.
LaTeX Info: Redefining \addtolength on input line 81.
\calc@Ccount=\count130
\calc@Cskip=\skip140
) (c:/TeXLive/2015/texmf-dist/tex/latex/geometry/geometry.sty
Package: geometry 2010/09/12 v5.6 Page Geometry
(c:/TeXLive/2015/texmf-dist/tex/generic/oberdiek/ifvtex.sty
Package: ifvtex 2010/03/01 v1.5 Detect VTeX and its facilities (HO)
Package ifvtex Info: VTeX not detected.
)
\Gm@cnth=\count131
\Gm@cntv=\count132
\c@Gm@tempcnt=\count133
\Gm@bindingoffset=\dimen154
\Gm@wd@mp=\dimen155
\Gm@odd@mp=\dimen156
\Gm@even@mp=\dimen157
\Gm@layoutwidth=\dimen158
\Gm@layoutheight=\dimen159
\Gm@layouthoffset=\dimen160
\Gm@layoutvoffset=\dimen161
\Gm@dimlist=\toks31
) (c:/TeXLive/2015/texmf-dist/tex/latex/hyperref/hyperref.sty
Package: hyperref 2012/11/06 v6.83m Hypertext links for LaTeX
(c:/TeXLive/2015/texmf-dist/tex/generic/oberdiek/hobsub-hyperref.sty
Package: hobsub-hyperref 2012/05/28 v1.13 Bundle oberdiek, subset
hyperref (HO)

(c:/TeXLive/2015/texmf-dist/tex/generic/oberdiek/hobsub-generic.sty
Package: hobsub-generic 2012/05/28 v1.13 Bundle oberdiek, subset generic
(HO)
Package: hobsub 2012/05/28 v1.13 Construct package bundles (HO)
Package hobsub Info: Skipping package `infwarerr' (already loaded).
Package hobsub Info: Skipping package `ltxcmds' (already loaded).
Package hobsub Info: Skipping package `ifluatex' (already loaded).
Package hobsub Info: Skipping package `ifvtex' (already loaded).
Package: intcalc 2007/09/27 v1.1 Expandable calculations with integers
(HO)
Package hobsub Info: Skipping package `ifpdf' (already loaded).
Package hobsub Info: Skipping package `etexcmds' (already loaded).
Package hobsub Info: Skipping package `kvsetkeys' (already loaded).
```

```
Package hobsub Info: Skipping package `kvdefinekeys' (already loaded).
Package hobsub Info: Skipping package `pdftexcmds' (already loaded).
Package: pdfescape 2011/11/25 v1.13 Implements pdfTeX's escape features
(HO)
Package: bigintcalc 2012/04/08 v1.3 Expandable calculations on big
integers (HO
)
Package: bitset 2011/01/30 v1.1 Handle bit-vector datatype (HO)
Package: uniquecounter 2011/01/30 v1.2 Provide unlimited unique counter
(HO)
)
Package hobsub Info: Skipping package `hobsub' (already loaded).
Package: letltxmacro 2010/09/02 v1.4 Let assignment for LaTeX macros (HO)
Package: hopatch 2012/05/28 v1.2 Wrapper for package hooks (HO)
Package: xcolor-patch 2011/01/30 xcolor patch
Package: atveryend 2011/06/30 v1.8 Hooks at the very end of document (HO)
Package hobsub Info: Skipping package `atbegshi' (already loaded).
Package: refcount 2011/10/16 v3.4 Data extraction from label references
(HO)
Package: hycolor 2011/01/30 v1.7 Color options for hyperref/bookmark (HO)
)
\@linkdim=\dimen162
\Hy@linkcounter=\count134
\Hy@pagecounter=\count135
(c:/TeXLive/2015/texmf-dist/tex/latex/hyperref/pd1enc.def
File: pd1enc.def 2012/11/06 v6.83m Hyperref: PDFDocEncoding definition
(HO)
Now handling font encoding PD1 ...
... no UTF-8 mapping file for font encoding PD1
)
\Hy@SavedSpaceFactor=\count136
(c:/TeXLive/2015/texmf-dist/tex/latex/latexconfig/hyperref.cfg
File: hyperref.cfg 2002/06/06 v1.2 hyperref configuration of TeXLive
)
Package hyperref Info: Option `colorlinks' set `true' on input line 4319.
Package hyperref Info: Hyper figures OFF on input line 4443.
Package hyperref Info: Link nesting OFF on input line 4448.
Package hyperref Info: Hyper index ON on input line 4451.
Package hyperref Info: Plain pages OFF on input line 4458.
Package hyperref Info: Backreferencing OFF on input line 4463.
Package hyperref Info: Implicit mode ON; LaTeX internals redefined.
Package hyperref Info: Bookmarks ON on input line 4688.
\c@Hy@tempcnt=\count137
LaTeX Info: Redefining \url on input line 5041.
\XeTeXLinkMargin=\dimen163
\Fld@menulength=\count138
\Field@Width=\dimen164
\Fld@charsize=\dimen165
Package hyperref Info: Hyper figures OFF on input line 6295.
Package hyperref Info: Link nesting OFF on input line 6300.
Package hyperref Info: Hyper index ON on input line 6303.
Package hyperref Info: backreferencing OFF on input line 6310.
Package hyperref Info: Link coloring ON on input line 6313.
Package hyperref Info: Link coloring with OCG OFF on input line 6320.
```

```
Package hyperref Info: PDF/A mode OFF on input line 6325.
LaTeX Info: Redefining \ref on input line 6365.
LaTeX Info: Redefining \pageref on input line 6369.
\Hy@abspage=\count139
\c@Item=\count140
\c@Hfootnote=\count141
)

Package hyperref Message: Driver (autodetected): hpdftex.

(c:/TeXLive/2015/texmf-dist/tex/latex/hyperref/hpdftex.def
File: hpdftex.def 2012/11/06 v6.83m Hyperref driver for pdfTeX
\HyAnn@Count=\count142
\Fld@listcount=\count143
\c@bookmark@seq@number=\count144
(c:/TeXLive/2015/texmf-dist/tex/latex/oberdiek/rerunfilecheck.sty
Package: rerunfilecheck 2011/04/15 v1.7 Rerun checks for auxiliary files
(HO)
Package uniquecounter Info: New unique counter `rerunfilecheck' on input
line 2
82.
)
\Hy@SectionHShift=\skip141
) (c:/TeXLive/2015/texmf-dist/tex/latex/preprint/authblk.sty
Package: authblk 2001/02/27 1.3 (PWD)
\affilsep=\skip142
\@affilsep=\skip143
\c@Maxaffil=\count145
\c@authors=\count146
\c@affil=\count147
) (c:/TeXLive/2015/texmf-dist/tex/latex/footmisc/footmisc.sty
Package: footmisc 2011/06/06 v5.5b a miscellany of footnote facilities
\FN@temptoken=\toks32
\footnotemargin=\dimen166
\c@pp@next@reset=\count148
Package footmisc Info: Declaring symbol style bringhurst on input line
855.
Package footmisc Info: Declaring symbol style chicago on input line 863.
Package footmisc Info: Declaring symbol style wiley on input line 872.
Package footmisc Info: Declaring symbol style lamport-robust on input
line 883.

Package footmisc Info: Declaring symbol style lamport* on input line 903.
Package footmisc Info: Declaring symbol style lamport*-robust on input
line 924
.
) (c:/TeXLive/2015/texmf-dist/tex/latex/fancyhdr/fancyhdr.sty
\fancy@headwidth=\skip144
\f@ncyO@elh=\skip145
\f@ncyO@erh=\skip146
\f@ncyO@olh=\skip147
\f@ncyO@orh=\skip148
\f@ncyO@elf=\skip149
\f@ncyO@erf=\skip150
```

```
\f@ncyO@olf=\skip151
\f@ncyO@orf=\skip152
) (c:/TeXLive/2015/texmf-dist/tex/generic/oberdiek/alphalph.sty
Package: alphalph 2011/05/13 v2.4 Convert numbers to letters (HO)
)
\c@authorfn=\count149
(c:/TeXLive/2015/texmf-dist/tex/latex/abstract/abstract.sty
Package: abstract 2009/06/08 v1.2a configurable abstracts
\abstitleskip=\skip153
\absleftindent=\skip154
\absrightindent=\skip155
\absparindent=\skip156
\absparsep=\skip157
)
Package newfloat Info: New float `keypoints' with options
`placement=t!,name=kp
t' on input line 286.
\c@keypoints=\count150
\newfloat@ftype=\count151
Package newfloat Info: float type `keypoints'=8 on input line 286.
(c:/TeXLive/2015/texmf-dist/tex/latex/enumitem/enumitem.sty
Package: enumitem 2011/09/28 v3.5.2 Customized lists
\labelindent=\skip158
\enit@outerparindent=\dimen167
\enit@toks=\toks33
\enit@inbox=\box52
\enitdp@description=\count152
) (c:/TeXLive/2015/texmf-dist/tex/latex/quoting/quoting.sty
Package: quoting 2014/01/28 v0.1c Consolidated environment for displayed
text
\quo@toppartop=\skip159
) (c:/TeXLive/2015/texmf-dist/tex/latex/sttools/stfloats.sty
Package: stfloats 2016/02/08 v2.0 Improve float mechanism and
baselineskip sett
ings
\@dblbotnum=\count153
\c@dblbotnumber=\count154
) (c:/TeXLive/2015/texmf-dist/tex/latex/booktabs/booktabs.sty
Package: booktabs 2005/04/14 v1.61803 publication quality tables
\heavyrulewidth=\dimen168
\lightrulewidth=\dimen169
\cmidrulewidth=\dimen170
\belowrulesep=\dimen171
\belowbottomsep=\dimen172
\aboverulesep=\dimen173
\abovetopsep=\dimen174
\cmidrulesep=\dimen175
\cmidrulekern=\dimen176
\defaultaddspace=\dimen177
\@cmidla=\count155
\@cmidlb=\count156
\@aboverulesep=\dimen178
\@belowrulesep=\dimen179
\@thisruleclass=\count157
```

```
\@lastruleclass=\count158
\@thisrulewidth=\dimen180
) (c:/TeXLive/2015/texmf-dist/tex/latex/tools/tabularx.sty
Package: tabularx 2016/02/03 v2.11 `tabularx' package (DPC)
\TX@col@width=\dimen181
\TX@old@table=\dimen182
\TX@old@col=\dimen183
\TX@target=\dimen184
\TX@delta=\dimen185
\TX@cols=\count159
\TX@ftn=\toks34
)
\enitdp@tablenotes=\count160
(c:/TeXLive/2015/texmf-dist/tex/latex/caption/caption.sty
Package: caption 2016/02/21 v3.3-144 Customizing captions (AR)
(c:/TeXLive/2015/texmf-dist/tex/latex/caption/caption3.sty
Package: caption3 2016/02/04 v1.7-139 caption3 kernel (AR)
Package caption3 Info: TeX engine: e-TeX on input line 67.
\captionmargin=\dimen186
\captionmargin@=\dimen187
\captionwidth=\dimen188
\caption@tempdima=\dimen189
\caption@indent=\dimen190
\caption@parindent=\dimen191
\caption@hangindent=\dimen192
)
\c@ContinuedFloat=\count161
Package caption Info: hyperref package is loaded.
Package caption Info: rotating package is loaded.
) (c:/TeXLive/2015/texmf-dist/tex/latex/natbib/natbib.sty
Package: natbib 2010/09/13 8.31b (PWD, AO)
\bibhang=\skip160
\bibsep=\skip161
LaTeX Info: Redefining \cite on input line 694.
\c@NAT@ctr=\count162
)) (c:/TeXLive/2015/texmf-dist/tex/latex/siunitx/siunitx.sty
Package: siunitx 2016/03/01 v2.6q A comprehensive (SI) units package
(c:/TeXLive/2015/texmf-dist/tex/latex/amsmath/amstext.sty
Package: amstext 2000/06/29 v2.01 AMS text
(c:/TeXLive/2015/texmf-dist/tex/latex/amsmath/amsgen.sty
File: amsgen.sty 1999/11/30 v2.0 generic functions
\@emptytoks=\toks35
\ex@=\dimen193
)) (c:/TeXLive/2015/texmf-dist/tex/latex/l3packages/l3keys2e/l3keys2e.sty
Package: l3keys2e 2016/03/28 v6468 LaTeX2e option processing using LaTeX3
keys
)
\l__siunitx_tmp_box=\box53
\l__siunitx_tmp_dim=\dimen194
\l__siunitx_tmp_int=\count163
\l__siunitx_number_mantissa_length_int=\count164
\l__siunitx_number_uncert_length_int=\count165
\l__siunitx_round_int=\count166
\l__siunitx_process_decimal_int=\count167
```

```
\l__siunitx_process_uncertainty_int=\count168
\l__siunitx_process_fixed_int=\count169
\l__siunitx_process_integer_min_int=\count170
\l__siunitx_process_precision_int=\count171
\l__siunitx_group_min_int=\count172
\l__siunitx_angle_marker_box=\box54
\l__siunitx_angle_unit_box=\box55
\l__siunitx_angle_marker_dim=\dimen195
\l__siunitx_angle_unit_dim=\dimen196
\l__siunitx_unit_int=\count173
\l__siunitx_unit_denominator_int=\count174
\l__siunitx_unit_numerator_int=\count175
\l__siunitx_unit_prefix_int=\count176
\l__siunitx_unit_prefix_base_int=\count177
\l__siunitx_unit_prefix_gram_int=\count178
\l__siunitx_number_product_int=\count179
\c__siunitx_one_fill_skip=\skip162
\l__siunitx_table_unit_align_skip=\skip163
\l__siunitx_table_exponent_dim=\dimen197
\l__siunitx_table_integer_dim=\dimen198
\l__siunitx_table_mantissa_dim=\dimen199
\l__siunitx_table_marker_dim=\dimen256
\l__siunitx_table_result_dim=\dimen257
\l__siunitx_table_uncert_dim=\dimen258
\l__siunitx_table_fill_pre_dim=\dimen259
\l__siunitx_table_fill_post_dim=\dimen260
\l__siunitx_table_fill_mid_dim=\dimen261
\l__siunitx_table_pre_box=\box56
\l__siunitx_table_post_box=\box57
\l__siunitx_table_mantissa_box=\box58
\l__siunitx_table_result_box=\box59
\l__siunitx_table_number_align_skip=\skip164
\l__siunitx_table_text_align_skip=\skip165
.................................................
. LaTeX info: "xparse/define-command"
.
. Defining command \DeclareBinaryPrefix with sig. 'mmm' on line 7218.
.................................................
.................................................
. LaTeX info: "xparse/define-command"
.
. Defining command \DeclareSIPostPower with sig. 'mm' on line 7221.
.................................................
.................................................
. LaTeX info: "xparse/define-command"
.
. Defining command \DeclareSIPrefix with sig. 'mmm' on line 7224.
.................................................
.................................................
. LaTeX info: "xparse/define-command"
.
. Defining command \DeclareSIPrePower with sig. 'mm' on line 7227.
.................................................
.................................................
```

```
. LaTeX info: "xparse/define-command"
.
. Defining command \DeclareSIQualifier with sig. 'mm' on line 7230.
................................................
................................................
. LaTeX info: "xparse/define-command"
.
. Defining command \DeclareSIUnit with sig. 'O{}mm' on line 7233.
................................................
................................................
. LaTeX info: "xparse/define-command"
.
. Defining command \DeclareSIUnitWithOptions with sig. 'mmm' on line
7236.
................................................
................................................
. LaTeX info: "xparse/define-command"
.
. Defining command \ang with sig. 'o>{\SplitArgument {2}{;}}m' on line
7251.
................................................
................................................
. LaTeX info: "xparse/define-command"
.
. Defining command \num with sig. 'om' on line 7260.
................................................
................................................
. LaTeX info: "xparse/define-command"
.
. Defining command \numlist with sig. 'o>{\SplitList {;}}m' on line 7269.
................................................
................................................
. LaTeX info: "xparse/define-command"
.
. Defining command \numrange with sig. 'omm' on line 7278.
................................................
................................................
. LaTeX info: "xparse/define-command"
.
. Defining command \SIlist with sig. 'o>{\SplitList {;}}mm' on line 7290.
................................................
................................................
. LaTeX info: "xparse/define-command"
.
. Defining command \SIrange with sig. 'ommm' on line 7302.
................................................
................................................
. LaTeX info: "xparse/define-command"
.
. Defining command \SI with sig. 'omom' on line 7314.
................................................
................................................
. LaTeX info: "xparse/define-command"
.
```

```
. Defining command \sisetup with sig. 'm' on line 7317.
.................................................
.................................................
. LaTeX info: "xparse/define-command"
.
. Defining command \tablenum with sig. 'om' on line 7332.
.................................................
.................................................
. LaTeX info: "xparse/define-command"
.
. Defining command \si with sig. 'om' on line 7344.
.................................................
.................................................
. LaTeX info: "xparse/define-command"
.
. Defining command \__siunitx_bookmark_num:w with sig. 'om' on line 7391.
.................................................
.................................................
. LaTeX info: "xparse/define-command"
.
. Defining command \__siunitx_bookmark_numrange:w with sig. 'omm' on line
. 7393.
.................................................
.................................................
. LaTeX info: "xparse/define-command"
.
. Defining command \__siunitx_bookmark_SI:w with sig. 'omom' on line
7395.
.................................................
.................................................
. LaTeX info: "xparse/define-command"
.
. Defining command \__siunitx_bookmark_SIlist:w with sig. 'omm' on line
7397.
.................................................
.................................................
. LaTeX info: "xparse/define-command"
.
. Defining command \__siunitx_bookmark_SIrange:w with sig. 'ommm' on line
. 7399.
.................................................
.................................................
. LaTeX info: "xparse/define-command"
.
. Defining command \__siunitx_bookmark_si:w with sig. 'om' on line 7400.
.................................................
\g__file_internal_ior=\read1
(c:/TeXLive/2015/texmf-dist/tex/latex/beamer/translator/translator.sty
Package: translator 2010/06/12 ver 1.10

(c:/TeXLive/2015/texmf-dist/tex/latex/beamer/translator/translator-
language-map
pings.tex))) (./main.aux)
\openout1 = `main.aux'.
```

```
LaTeX Font Info:    Checking defaults for OML/cmm/m/it on input line 51.
LaTeX Font Info:    ... okay on input line 51.
LaTeX Font Info:    Checking defaults for T1/cmr/m/n on input line 51.
LaTeX Font Info:    ... okay on input line 51.
LaTeX Font Info:    Checking defaults for OT1/cmr/m/n on input line 51.
LaTeX Font Info:    ... okay on input line 51.
LaTeX Font Info:    Checking defaults for OMS/cmsy/m/n on input line 51.
LaTeX Font Info:    ... okay on input line 51.
LaTeX Font Info:    Checking defaults for OMX/cmex/m/n on input line 51.
LaTeX Font Info:    ... okay on input line 51.
LaTeX Font Info:    Checking defaults for U/cmr/m/n on input line 51.
LaTeX Font Info:    ... okay on input line 51.
LaTeX Font Info:    Checking defaults for TS1/cmr/m/n on input line 51.
LaTeX Font Info:    Try loading font information for TS1+cmr on input
line 51.
(c:/TeXLive/2015/texmf-dist/tex/latex/base/ts1cmr.fd
File: ts1cmr.fd 2014/09/29 v2.5h Standard LaTeX font definitions
)
LaTeX Font Info:    ... okay on input line 51.
LaTeX Font Info:    Checking defaults for PD1/pdf/m/n on input line 51.
LaTeX Font Info:    ... okay on input line 51.
LaTeX Font Info:    Try loading font information for T1+Merriweather-OsF
on inp
ut line 51.
(c:/TeXLive/2015/texmf-dist/tex/latex/merriweather/t1merriweather-osf.fd
File: T1Merriweather-OsF.fd 2014/01/22 (autoinst) Font definitions for
T1/Merri
weather-OsF.
)
LaTeX Font Info:    Font shape `T1/Merriweather-OsF/m/n' will be
(Font)              scaled to size 7.5pt on input line 51.
LaTeX Info: Redefining \microtypecontext on input line 51.
Package microtype Info: Generating PDF output.
Package microtype Info: Character protrusion enabled (level 2).
Package microtype Info: Using default protrusion set `alltext'.
Package microtype Info: Automatic font expansion enabled (level 2),
(microtype)             stretch: 20, shrink: 20, step: 1, non-selected.
Package microtype Info: Using default expansion set `basictext'.
Package microtype Info: No adjustment of tracking.
Package microtype Info: No adjustment of interword spacing.
Package microtype Info: No adjustment of character kerning.
Package microtype Info: Loading generic settings for font family
(microtype)             `Merriweather-OsF' (encoding: T1).
(microtype)             For optimal results, create family-specific
settings.
(microtype)             See the microtype manual for details.
LaTeX Font Info:    Redeclaring symbol font `operators' on input line 51.
LaTeX Font Info:    Encoding `OT1' has changed to `T1' for symbol font
(Font)              `operators' in the math version `normal' on input
line 51.
LaTeX Font Info:    Overwriting symbol font `operators' in version
`normal'
```

```
(Font)                  OT1/cmr/m/n --> T1/Merriweather-OsF/m/n on input
line 5
1.
LaTeX Font Info:    Encoding `OT1' has changed to `T1' for symbol font
(Font)              `operators' in the math version `bold' on input line
51.
LaTeX Font Info:    Overwriting symbol font `operators' in version `bold'
(Font)                  OT1/cmr/bx/n --> T1/Merriweather-OsF/m/n on input
line
51.
LaTeX Font Info:    Overwriting symbol font `operators' in version `bold'
(Font)                  T1/Merriweather-OsF/m/n --> T1/Merriweather-
OsF/bx/n on
 input line 51.
LaTeX Font Info:    Redeclaring math alphabet \mathbf on input line 51.
LaTeX Font Info:    Overwriting math alphabet `\mathbf' in version
`normal'
(Font)                  OT1/cmr/bx/n --> T1/Merriweather-OsF/bx/n on
input line
 51.
LaTeX Font Info:    Overwriting math alphabet `\mathbf' in version `bold'
(Font)                  OT1/cmr/bx/n --> T1/Merriweather-OsF/bx/n on
input line
 51.
LaTeX Font Info:    Redeclaring math alphabet \mathsf on input line 51.
LaTeX Font Info:    Overwriting math alphabet `\mathsf' in version
`normal'
(Font)                  OT1/cmss/m/n --> T1/MerriweatherSans-TLF/m/n on
input l
ine 51.
LaTeX Font Info:    Overwriting math alphabet `\mathsf' in version `bold'
(Font)                  OT1/cmss/bx/n --> T1/MerriweatherSans-TLF/m/n on
input
line 51.
LaTeX Font Info:    Redeclaring math alphabet \mathit on input line 51.
LaTeX Font Info:    Overwriting math alphabet `\mathit' in version
`normal'
(Font)                  OT1/cmr/m/it --> T1/Merriweather-OsF/m/it on
input line
 51.
LaTeX Font Info:    Overwriting math alphabet `\mathit' in version `bold'
(Font)                  OT1/cmr/bx/it --> T1/Merriweather-OsF/m/it on
input lin
e 51.
LaTeX Font Info:    Redeclaring math alphabet \mathtt on input line 51.
LaTeX Font Info:    Overwriting math alphabet `\mathtt' in version
`normal'
(Font)                  OT1/cmtt/m/n --> T1/lmtt/m/n on input line 51.
LaTeX Font Info:    Overwriting math alphabet `\mathtt' in version `bold'
(Font)                  OT1/cmtt/m/n --> T1/lmtt/m/n on input line 51.
LaTeX Font Info:    Overwriting math alphabet `\mathsf' in version `bold'
(Font)                  T1/MerriweatherSans-TLF/m/n -->
T1/MerriweatherSans-TLF
/bx/n on input line 51.
```

```
LaTeX Font Info:    Overwriting math alphabet `\mathit' in version `bold'
(Font)                  T1/Merriweather-OsF/m/it --> T1/Merriweather-
OsF/bx/it
on input line 51.
\c@mv@tabular=\count180
\c@mv@boldtabular=\count181
ABD: EverySelectfont initializing macros
LaTeX Info: Redefining \selectfont on input line 51.
(c:/TeXLive/2015/texmf-dist/tex/context/base/supp-pdf.mkii
[Loading MPS to PDF converter (version 2006.09.02).]
\scratchcounter=\count182
\scratchdimen=\dimen262
\scratchbox=\box60
\nofMPsegments=\count183
\nofMParguments=\count184
\everyMPshowfont=\toks36
\MPscratchCnt=\count185
\MPscratchDim=\dimen263
\MPnumerator=\count186
\makeMPintoPDFobject=\count187
\everyMPtoPDFconversion=\toks37
) (c:/TeXLive/2015/texmf-dist/tex/latex/oberdiek/epstopdf-base.sty
Package: epstopdf-base 2010/02/09 v2.5 Base part for package epstopdf
(c:/TeXLive/2015/texmf-dist/tex/latex/oberdiek/grfext.sty
Package: grfext 2010/08/19 v1.1 Manage graphics extensions (HO)
)
Package grfext Info: Graphics extension search list:
(grfext)
[.png,.pdf,.jpg,.mps,.jpeg,.jbig2,.jb2,.PNG,.PDF,.JPG,.JPE
G,.JBIG2,.JB2,.eps]
(grfext)             \AppendGraphicsExtensions on input line 452.
(c:/TeXLive/2015/texmf-dist/tex/latex/latexconfig/epstopdf-sys.cfg
File: epstopdf-sys.cfg 2010/07/13 v1.3 Configuration of (r)epstopdf for
TeX Liv
e
))
Package lastpage Info: Please have a look at the pageslts package at
(lastpage)             https://www.ctan.org/pkg/pageslts
(lastpage)             ! on input line 51.
ABD: EveryShipout initializing macros
\AtBeginShipoutBox=\box61
*geometry* driver: auto-detecting
*geometry* detected driver: pdftex
*geometry* verbose mode - [ preamble ] result:
* driver: pdftex
* paper: a4paper
* layout: <same size as paper>
* layoutoffset:(h,v)=(0.0pt,0.0pt)
* modes: includefoot twoside
* h-part:(L,W,R)=(54.64pt, 488.22787pt, 54.64pt)
* v-part:(T,H,B)=(66.0pt, 745.04684pt, 34.0pt)
* \paperwidth=597.50787pt
* \paperheight=845.04684pt
* \textwidth=488.22787pt
```

```
* \textheight=715.04684pt
* \oddsidemargin=-17.62999pt
* \evensidemargin=-17.62999pt
* \topmargin=-47.76999pt
* \headheight=17.5pt
* \headsep=24.0pt
* \topskip=10.0pt
* \footskip=30.0pt
* \marginparwidth=48.0pt
* \marginparsep=10.0pt
* \columnsep=18.0pt
* \skip\footins=22.0pt plus 2.0pt
* \hoffset=0.0pt
* \voffset=0.0pt
* \mag=1000
* \@twocolumntrue
* \@twosidetrue
* \@mparswitchtrue
* \@reversemarginfalse
* (1in=72.27pt=25.4mm, 1cm=28.453pt)

Package hyperref Info: Link coloring ON on input line 51.
(c:/TeXLive/2015/texmf-dist/tex/latex/hyperref/nameref.sty
Package: nameref 2012/10/27 v2.43 Cross-referencing by name of section
(c:/TeXLive/2015/texmf-dist/tex/generic/oberdiek/gettitlestring.sty
Package: gettitlestring 2010/12/03 v1.4 Cleanup title references (HO)
)
\c@section@level=\count188
)
LaTeX Info: Redefining \ref on input line 51.
LaTeX Info: Redefining \pageref on input line 51.
LaTeX Info: Redefining \nameref on input line 51.
(./main.out) (./main.out)
\@outlinefile=\write3
\openout3 = `main.out'.

\@gscitedetails=\box62
\@gscitedetailsheight=\skip166
\@gsheadbox=\box63
\@gsheadboxheight=\skip167
LaTeX Font Info:    Font shape `T1/Merriweather-OsF/b/n' will be
(Font)              scaled to size 6.5pt on input line 51.
LaTeX Font Info:    Calculating math sizes for size <7.5> on input line
51.
LaTeX Font Info:    Font shape `T1/Merriweather-OsF/m/n' will be
(Font)              scaled to size 6.24973pt on input line 51.
LaTeX Font Info:    Font shape `T1/Merriweather-OsF/m/n' will be
(Font)              scaled to size 5.24997pt on input line 51.
LaTeX Font Info:    Try loading font information for U+eur on input line
51.
(c:/TeXLive/2015/texmf-dist/tex/latex/amsfonts/ueur.fd
File: ueur.fd 2013/01/14 v3.01 Euler Roman
) (c:/TeXLive/2015/texmf-dist/tex/latex/microtype/mt-eur.cfg
```

```
File: mt-eur.cfg 2006/07/31 v1.1 microtype config. file: AMS Euler Roman
(RS)
)

LaTeX Font Warning: Font shape `OMS/cmsy/m/n' in size <7.5> not available
(Font)              size <7> substituted on input line 51.

LaTeX Font Info:    External font `cmex10' loaded for size
(Font)              <7.5> on input line 51.
LaTeX Font Info:    External font `cmex10' loaded for size
(Font)              <6.24973> on input line 51.
LaTeX Font Info:    External font `cmex10' loaded for size
(Font)              <5.24997> on input line 51.
LaTeX Font Info:    Try loading font information for U+euf on input line
51.
(c:/TeXLive/2015/texmf-dist/tex/latex/amsfonts/ueuf.fd
File: ueuf.fd 2013/01/14 v3.01 Euler Fraktur
) (c:/TeXLive/2015/texmf-dist/tex/latex/microtype/mt-euf.cfg
File: mt-euf.cfg 2006/07/03 v1.1 microtype config. file: AMS Euler
Fraktur (RS)

)
LaTeX Font Info:    Try loading font information for U+eus on input line
51.
(c:/TeXLive/2015/texmf-dist/tex/latex/amsfonts/ueus.fd
File: ueus.fd 2013/01/14 v3.01 Euler Script
) (c:/TeXLive/2015/texmf-dist/tex/latex/microtype/mt-eus.cfg
File: mt-eus.cfg 2006/07/28 v1.2 microtype config. file: AMS Euler Script
(RS)
)
LaTeX Font Info:    Try loading font information for U+euex on input line
51.
(c:/TeXLive/2015/texmf-dist/tex/latex/amsfonts/ueuex.fd
File: ueuex.fd 2013/01/14 v3.01 Euler extra symbols
)

LaTeX Font Warning: Font shape `OML/cmm/m/it' in size <7.5> not available
(Font)              size <7> substituted on input line 51.

LaTeX Font Info:    Font shape `T1/Merriweather-OsF/m/it' will be
(Font)              scaled to size 7.5pt on input line 51.
LaTeX Font Info:    Font shape `T1/Merriweather-OsF/m/it' will be
(Font)              scaled to size 6.24973pt on input line 51.
LaTeX Font Info:    Font shape `T1/Merriweather-OsF/m/it' will be
(Font)              scaled to size 5.24997pt on input line 51.
LaTeX Font Info:    Font shape `T1/Merriweather-OsF/m/n' will be
(Font)              scaled to size 8.0pt on input line 51.
LaTeX Font Info:    Font shape `T1/Merriweather-OsF/m/it' will be
(Font)              scaled to size 8.0pt on input line 51.
LaTeX Font Info:    Font shape `T1/Merriweather-OsF/b/it' will be
(Font)              scaled to size 8.0pt on input line 51.
Package caption Info: Begin \AtBeginDocument code.
Package caption Info: End \AtBeginDocument code.
```

```
(c:/TeXLive/2015/texmf-dist/tex/latex/beamer/translator/dicts/translator-
basic-
dictionary/translator-basic-dictionary-English.dict
Dictionary: translator-basic-dictionary, Language: English
) (c:/TeXLive/2015/texmf-dist/tex/latex/siunitx/siunitx-abbreviations.cfg
File: siunitx-abbreviations.cfg 2016/03/01 v2.6q siunitx: Abbreviated
units
)
\c__siunitx_mathsf_int=\count189
LaTeX Font Info:    Try loading font information for T1+MerriweatherSans-
TLF on
 input line 51.
(c:/TeXLive/2015/texmf-dist/tex/latex/merriweather/t1merriweathersans-
tlf.fd
File: T1MerriweatherSans-TLF.fd 2014/01/22 (autoinst) Font definitions
for T1/M
erriweatherSans-TLF.
)
LaTeX Font Info:    Font shape `T1/MerriweatherSans-TLF/m/n' will be
(Font)              scaled to size 7.5pt on input line 51.
Package microtype Info: Loading generic settings for font family
(microtype)             `MerriweatherSans-TLF' (encoding: T1).
(microtype)             For optimal results, create family-specific
settings.
(microtype)             See the microtype manual for details.
LaTeX Font Info:    Font shape `T1/MerriweatherSans-TLF/m/n' will be
(Font)              scaled to size 6.24973pt on input line 51.
LaTeX Font Info:    Font shape `T1/MerriweatherSans-TLF/m/n' will be
(Font)              scaled to size 5.24997pt on input line 51.
\c__siunitx_mathtt_int=\count190
LaTeX Font Info:    Try loading font information for T1+lmtt on input
line 51.
(c:/TeXLive/2015/texmf-dist/tex/latex/lm/t1lmtt.fd
File: t1lmtt.fd 2009/10/30 v1.6 Font defs for Latin Modern
)
Package microtype Info: Loading generic settings for font family
(microtype)             `lmtt' (encoding: T1).
(microtype)             For optimal results, create family-specific
settings.
(microtype)             See the microtype manual for details.
TextBlockOrigin set to 4pc+6.64pt x 4pc+6pt
! Undefined control sequence.
<argument> \@ouplogo

l.63 \end{frontmatter}

The control sequence at the end of the top line
of your error message was never \def'ed. If you have
misspelled it (e.g., `\hobx'), type `I' and the correct
spelling (e.g., `I\hbox'). Otherwise just continue,
and I'll forget about whatever was undefined.
```

```
LaTeX Warning: Command \textasciiacute invalid in math mode on input line
63.

LaTeX Font Info:    Try loading font information for TS1+Merriweather-OsF
on in
put line 63.
(c:/TeXLive/2015/texmf-dist/tex/latex/merriweather/ts1merriweather-osf.fd
File: TS1Merriweather-OsF.fd 2014/01/22 (autoinst) Font definitions for
TS1/Mer
riweather-OsF.
)
LaTeX Font Info:    Font shape `TS1/Merriweather-OsF/m/n' will be
(Font)              scaled to size 7.5pt on input line 63.
Package microtype Info: Loading generic settings for font family
(microtype)             `Merriweather-OsF' (encoding: TS1).
(microtype)             For optimal results, create family-specific
settings.
(microtype)             See the microtype manual for details.

LaTeX Warning: Command \textasciiacute invalid in math mode on input line
63.

! Extra }, or forgotten $.
\TP@commonendtextblock ...oxrulesize }\fi \egroup
                                                  \TP@prevdepth
=\prevdepth ...
l.63 \end{frontmatter}

I've deleted a group-closing symbol because it seems to be
spurious, as in `$x}$'. But perhaps the } is legitimate and
you forgot something else, as in `\hbox{$x}'. In such cases
the way to recover is to insert both the forgotten and the
deleted material, e.g., by typing `I$}'.

! Improper \prevdepth.
\TP@commonendtextblock ...P@prevdepth =\prevdepth
                                                  \prevdepth =-1000pt
\expan...
l.63 \end{frontmatter}

You can refer to \spacefactor only in horizontal mode;
you can refer to \prevdepth only in vertical mode; and
neither of these is meaningful inside \write. So
I'm forgetting what you said and using zero instead.

! You can't use `\prevdepth' in math mode.
\TP@commonendtextblock ... =\prevdepth \prevdepth
                                                  =-1000pt \expandafter
\TP@...
l.63 \end{frontmatter}

Sorry, but I'm not programmed to handle this case;
I'll just pretend that you didn't ask for it.
If you're in the wrong mode, you might be able to
```

```
return to the right one by typing `I}' or `I$' or `I\par'.

! Missing $ inserted.
<inserted text> 
                $
l.63 \end{frontmatter}

I've inserted something that you may have forgotten.
(See the <inserted text> above.)
With luck, this will get me unwedged. But if you
really didn't forget anything, try typing `2' now; then
my insertion and my current dilemma will both disappear.

! Missing } inserted.
<inserted text> 
                }
l.63 \end{frontmatter}

I've inserted something that you may have forgotten.
(See the <inserted text> above.)
With luck, this will get me unwedged. But if you
really didn't forget anything, try typing `2' now; then
my insertion and my current dilemma will both disappear.


Overfull \hbox (54.64pt too wide) in paragraph at lines 63--63
[][]
 []

LaTeX Font Info:    Font shape `T1/Merriweather-OsF/m/n' will be
(Font)              scaled to size 14.0pt on input line 63.
LaTeX Font Info:    Font shape `T1/Merriweather-OsF/m/n' will be
(Font)              scaled to size 8.99997pt on input line 63.
LaTeX Font Info:    Font shape `T1/Merriweather-OsF/b/n' will be
(Font)              scaled to size 18.0pt on input line 63.
LaTeX Font Info:    Font shape `T1/Merriweather-OsF/m/n' will be
(Font)              scaled to size 13.0pt on input line 63.
LaTeX Font Info:    Calculating math sizes for size <13> on input line
63.
LaTeX Font Info:    Font shape `T1/Merriweather-OsF/m/n' will be
(Font)              scaled to size 10.83287pt on input line 63.
LaTeX Font Info:    Font shape `T1/Merriweather-OsF/m/n' will be
(Font)              scaled to size 9.09996pt on input line 63.

LaTeX Font Warning: Font shape `OMS/cmsy/m/n' in size <13> not available
(Font)              size <12> substituted on input line 63.

LaTeX Font Info:    External font `cmex10' loaded for size
(Font)              <13> on input line 63.
LaTeX Font Info:    External font `cmex10' loaded for size
(Font)              <10.83287> on input line 63.
LaTeX Font Info:    External font `cmex10' loaded for size
(Font)              <9.09996> on input line 63.
```

```
LaTeX Font Warning: Font shape `OML/cmm/m/it' in size <13> not available
(Font)              size <12> substituted on input line 63.

LaTeX Font Info:    Font shape `T1/Merriweather-OsF/m/it' will be
(Font)              scaled to size 13.0pt on input line 63.
LaTeX Font Info:    Font shape `T1/Merriweather-OsF/m/it' will be
(Font)              scaled to size 10.83287pt on input line 63.
LaTeX Font Info:    Font shape `T1/Merriweather-OsF/m/it' will be
(Font)              scaled to size 9.09996pt on input line 63.
LaTeX Font Info:    Font shape `T1/MerriweatherSans-TLF/m/n' will be
(Font)              scaled to size 13.0pt on input line 63.
LaTeX Font Info:    Font shape `T1/MerriweatherSans-TLF/m/n' will be
(Font)              scaled to size 10.83287pt on input line 63.
LaTeX Font Info:    Font shape `T1/MerriweatherSans-TLF/m/n' will be
(Font)              scaled to size 9.09996pt on input line 63.
LaTeX Font Info:    Font shape `TS1/Merriweather-OsF/m/n' will be
(Font)              scaled to size 10.83287pt on input line 63.
LaTeX Font Info:    Font shape `T1/Merriweather-OsF/m/n' will be
(Font)              scaled to size 9.0pt on input line 63.
LaTeX Font Info:    Font shape `T1/Merriweather-OsF/m/n' will be
(Font)              scaled to size 7.0pt on input line 63.
LaTeX Font Info:    Font shape `T1/Merriweather-OsF/m/n' will be
(Font)              scaled to size 5.0pt on input line 63.
LaTeX Font Info:    External font `cmex10' loaded for size
(Font)              <9> on input line 63.
LaTeX Font Info:    External font `cmex10' loaded for size
(Font)              <7> on input line 63.
LaTeX Font Info:    External font `cmex10' loaded for size
(Font)              <5> on input line 63.
LaTeX Font Info:    Font shape `T1/Merriweather-OsF/m/it' will be
(Font)              scaled to size 9.0pt on input line 63.
LaTeX Font Info:    Font shape `T1/Merriweather-OsF/m/it' will be
(Font)              scaled to size 7.0pt on input line 63.
LaTeX Font Info:    Font shape `T1/Merriweather-OsF/m/it' will be
(Font)              scaled to size 5.0pt on input line 63.
LaTeX Font Info:    Font shape `T1/MerriweatherSans-TLF/m/n' will be
(Font)              scaled to size 9.0pt on input line 63.
LaTeX Font Info:    Font shape `T1/MerriweatherSans-TLF/m/n' will be
(Font)              scaled to size 7.0pt on input line 63.
LaTeX Font Info:    Font shape `T1/MerriweatherSans-TLF/m/n' will be
(Font)              scaled to size 5.0pt on input line 63.
LaTeX Font Info:    Font shape `T1/Merriweather-OsF/m/n' will be
(Font)              scaled to size 6.5pt on input line 63.
LaTeX Font Info:    Calculating math sizes for size <6.5> on input line
63.
LaTeX Font Info:    Font shape `T1/Merriweather-OsF/m/n' will be
(Font)              scaled to size 5.41643pt on input line 63.
LaTeX Font Info:    Font shape `T1/Merriweather-OsF/m/n' will be
(Font)              scaled to size 4.54997pt on input line 63.

LaTeX Font Warning: Font shape `OMS/cmsy/m/n' in size <6.5> not available
(Font)              size <6> substituted on input line 63.
```

```
LaTeX Font Warning: Font shape `OMS/cmsy/m/n' in size <5.41643> not
available
(Font)              size <5> substituted on input line 63.


LaTeX Font Warning: Font shape `OMS/cmsy/m/n' in size <4.54997> not
available
(Font)              size <5> substituted on input line 63.

LaTeX Font Info:    External font `cmex10' loaded for size
(Font)              <6.5> on input line 63.
LaTeX Font Info:    External font `cmex10' loaded for size
(Font)              <5.41643> on input line 63.
LaTeX Font Info:    External font `cmex10' loaded for size
(Font)              <4.54997> on input line 63.

LaTeX Font Warning: Font shape `OML/cmm/m/it' in size <6.5> not available
(Font)              size <6> substituted on input line 63.


LaTeX Font Warning: Font shape `OML/cmm/m/it' in size <5.41643> not
available
(Font)              size <5> substituted on input line 63.


LaTeX Font Warning: Font shape `OML/cmm/m/it' in size <4.54997> not
available
(Font)              size <5> substituted on input line 63.

LaTeX Font Info:    Font shape `T1/Merriweather-OsF/m/it' will be
(Font)              scaled to size 6.5pt on input line 63.
LaTeX Font Info:    Font shape `T1/Merriweather-OsF/m/it' will be
(Font)              scaled to size 5.41643pt on input line 63.
LaTeX Font Info:    Font shape `T1/Merriweather-OsF/m/it' will be
(Font)              scaled to size 4.54997pt on input line 63.
LaTeX Font Info:    Font shape `T1/MerriweatherSans-TLF/m/n' will be
(Font)              scaled to size 6.5pt on input line 63.
LaTeX Font Info:    Font shape `T1/MerriweatherSans-TLF/m/n' will be
(Font)              scaled to size 5.41643pt on input line 63.
LaTeX Font Info:    Font shape `T1/MerriweatherSans-TLF/m/n' will be
(Font)              scaled to size 4.54997pt on input line 63.
LaTeX Font Info:    Font shape `TS1/Merriweather-OsF/m/n' will be
(Font)              scaled to size 5.41643pt on input line 63.

Overfull \hbox (54.64pt too wide) in paragraph at lines 63--63
[][][]
 []

LaTeX Font Info:    Font shape `T1/Merriweather-OsF/b/n' will be
(Font)              scaled to size 10.0pt on input line 63.
LaTeX Font Info:    Font shape `T1/Merriweather-OsF/b/n' will be
(Font)              scaled to size 8.0pt on input line 63.

Overfull \hbox (54.64pt too wide) in paragraph at lines 63--63
```

```
[][][]
 []

Package mdframed Info: mdframed works in twoside mode on input line 66.
LaTeX Font Info:    Font shape `T1/Merriweather-OsF/b/n' will be
(Font)              scaled to size 8.2pt on input line 66.
LaTeX Font Info:    Font shape `T1/Merriweather-OsF/b/n' will be
(Font)              scaled to size 7.5pt on input line 72.
Package mdframed Info: mdframed inside float
 mdframed uses option nobreak mdframed on input line 79.
Package mdframed Info: mdframed inside a box
 mdframed uses option nobreak mdframed on input line 79.

Package natbib Warning: Citation `Sandberg2014-dq' on page 1 undefined on
input
 line 83.


Package natbib Warning: Citation `Zheng2017-zx' on page 1 undefined on
input li
ne 83.


Package natbib Warning: Citation `Rosenberg2018-aj' on page 1 undefined
on inpu
t line 83.


Package natbib Warning: Citation `Wagner2016-ga' on page 1 undefined on
input l
ine 83.


Package natbib Warning: Citation `Regev2017-hc' on page 1 undefined on
input li
ne 83.


Package natbib Warning: Citation `Parekh2016-lk' on page 1 undefined on
input l
ine 84.


Package natbib Warning: Citation `Kivioja2012-cy' on page 1 undefined on
input
line 84.


Package natbib Warning: Citation `Ziegenhain2017-ww' on page 1 undefined
on inp
ut line 84.
```

```
Package natbib Warning: Citation `Vieth2017-yl' on page 1 undefined on
input li
ne 84.


Package natbib Warning: Citation `Ziegenhain2018-qz' on page 1 undefined
on inp
ut line 85.


Package natbib Warning: Citation `Parekh2016-lk' on page 1 undefined on
input l
ine 85.


Package natbib Warning: Citation `Lake2016-ql' on page 1 undefined on
input lin
e 86.


Package natbib Warning: Citation `Habib2017-sn' on page 1 undefined on
input li
ne 86.


Package natbib Warning: Citation `Lake2016-ql' on page 1 undefined on
input lin
e 86.


Package natbib Warning: Citation `Macosko2015-yd' on page 1 undefined on
input
line 87.


Package natbib Warning: Citation `Svensson2017-gy' on page 1 undefined on
input
 line 87.


Package natbib Warning: Citation `Hashimshony2016-nk' on page 1 undefined
on in
put line 87.


Package natbib Warning: Citation `Petukhov2017-ui' on page 1 undefined on
input
 line 87.


Underfull \vbox (badness 10000) has occurred while \output is active []


Underfull \vbox (badness 10000) has occurred while \output is active []
```

```
LaTeX Font Info:    Font shape `T1/Merriweather-OsF/m/n' will be
(Font)              scaled to size 7.8pt on input line 88.
LaTeX Font Info:    Font shape `T1/Merriweather-OsF/b/n' will be
(Font)              scaled to size 7.8pt on input line 88.
[1{c:/TeXLive/2015/texmf-var/fonts/map/pdftex/updmap/pdftex.map}


]
LaTeX Font Info:    Font shape `T1/Merriweather-OsF/b/n' will be
(Font)              scaled to size 8.5pt on input line 89.

Package natbib Warning: Citation `Soumillon2014-hd' on page 2 undefined
on inpu
t line 92.


Package natbib Warning: Citation `Jaitin2014-na' on page 2 undefined on
input l
ine 92.


Package natbib Warning: Citation `Macosko2015-yd' on page 2 undefined on
input
line 92.


Package natbib Warning: Citation `Hashimshony2016-nk' on page 2 undefined
on in
put line 92.


Package natbib Warning: Citation `Rosenberg2018-aj' on page 2 undefined
on inpu
t line 92.


Package natbib Warning: Citation `Zheng2017-zx' on page 2 undefined on
input li
ne 92.


Package natbib Warning: Citation `Habib2017-sn' on page 2 undefined on
input li
ne 92.


Package natbib Warning: Citation `Klein2015-mj' on page 2 undefined on
input li
ne 92.


Package natbib Warning: Citation `Zilionis2017-ox' on page 2 undefined on
input
```

```
 line 92.


Package natbib Warning: Citation `Hochgerner2017-wt' on page 2 undefined
on inp
ut line 92.

LaTeX Font Info:    Font shape `T1/Merriweather-OsF/b/sl' in size <7.5>
not ava
ilable
(Font)              Font shape `T1/Merriweather-OsF/b/it' tried instead
on inpu
t line 96.
LaTeX Font Info:    Font shape `T1/Merriweather-OsF/b/it' will be
(Font)              scaled to size 7.5pt on input line 96.

Package natbib Warning: Citation `Dobin2013-ha' on page 2 undefined on
input li
ne 100.


Package natbib Warning: Citation `Liao2014-pg' on page 2 undefined on
input lin
e 103.


Package natbib Warning: Citation `data.table-xy' on page 2 undefined on
input l
ine 103.


Package natbib Warning: Citation `Smith2017-xd' on page 2 undefined on
input li
ne 105.


Package natbib Warning: Citation `Smith2017-xd' on page 2 undefined on
input li
ne 105.


Package natbib Warning: Citation `Smith2017-xd' on page 2 undefined on
input li
ne 107.


Package natbib Warning: Citation `Macosko2015-yd' on page 2 undefined on
input
line 107.

! Undefined control sequence.
l.111 ...distribution using the R-package \texttt
                                                  {mclust}
\cite{Fraley2002-...
```

```
The control sequence at the end of the top line
of your error message was never \def'ed. If you have
misspelled it (e.g., `\hobx'), type `I' and the correct
spelling (e.g., `I\hbox'). Otherwise just continue,
and I'll forget about whatever was undefined.


Package natbib Warning: Citation `Fraley2002-wc' on page 2 undefined on
input l
ine 111.


Package natbib Warning: Citation `Fraley2012-bb' on page 2 undefined on
input l
ine 111.


Package natbib Warning: Citation `Habib2017-sn' on page 2 undefined on
input li
ne 112.

LaTeX Font Info:    Font shape `T1/Merriweather-OsF/m/it' will be
(Font)              scaled to size 7.8pt on input line 114.

Overfull \hbox (253.11394pt too wide) has occurred while \output is
active
[][]
 []

[2]

Package natbib Warning: Citation `Vallejos2017-gp' on page 3 undefined on
input
 line 117.


Package natbib Warning: Citation `Evans2017-hj' on page 3 undefined on
input li
ne 117.


Package natbib Warning: Citation `Grun2015-tk' on page 3 undefined on
input lin
e 117.


Package natbib Warning: Citation `Ziegenhain2017-ww' on page 3 undefined
on inp
ut line 119.


Package natbib Warning: Citation `Svensson2017-gy' on page 3 undefined on
input
 line 119.
```

```
Package natbib Warning: Citation `Hendriks2014-lj' on page 3 undefined on
input
 line 131.


Package natbib Warning: Citation `Gaidatzis2015-lx' on page 3 undefined
on inpu
t line 131.


Package natbib Warning: Citation `La_Manno2017-mu' on page 3 undefined on
input
 line 131.


Package natbib Warning: Citation `Habib2017-sn' on page 3 undefined on
input li
ne 131.


Package natbib Warning: Citation `Lake2016-ql' on page 3 undefined on
input lin
e 132.


Package natbib Warning: Citation `Lake2017-dn' on page 3 undefined on
input lin
e 132.


Package natbib Warning: Citation `Satija2015-nn' on page 3 undefined on
input l
ine 132.


Package natbib Warning: Citation `Butler2017-kv' on page 3 undefined on
input l
ine 132.


Package natbib Warning: Citation `Tasic2016-ms' on page 3 undefined on
input li
ne 136.


Package natbib Warning: Citation `Tasic2016-ms' on page 3 undefined on
input li
ne 137.


Package natbib Warning: Citation `Vieth2017-yl' on page 3 undefined on
input li
```

```
ne 139.


Package natbib Warning: Citation `Habib2017-sn' on page 3 undefined on
input li
ne 139.


Package natbib Warning: Citation `The_Tabula_Muris_Consortium2018-wm' on
page 3
 undefined on input line 139.


Package natbib Warning: Citation `Han2018-ce' on page 3 undefined on
input line
 139.


Overfull \hbox (253.11394pt too wide) has occurred while \output is
active
[][]
 []

[3]

Package natbib Warning: Citation `Bagnoli2017-ex' on page 4 undefined on
input
line 155.


Underfull \hbox (badness 3039) in paragraph at lines 153--156
\T1/Merriweather-OsF/m/n/7.5 Serum (Thermo Fisher) and 1 % Peni-
cillin/Streptom
ycin
 []


Package natbib Warning: Citation `Zheng2017-zx' on page 4 undefined on
input li
ne 157.


Package natbib Warning: Citation `Habib2017-sn' on page 4 undefined on
input li
ne 159.


Package natbib Warning: Citation `noauthor_undated-nd' on page 4
undefined on i
nput line 159.


Package natbib Warning: Citation `Vieth2017-yl' on page 4 undefined on
input li
```

```
ne 164.


Package natbib Warning: Citation `Law2014-yx' on page 4 undefined on
input line
 164.


Package natbib Warning: Citation `Lun2016-qr' on page 4 undefined on
input line
 164.


Package natbib Warning: Citation `Soneson2018-ms' on page 4 undefined on
input
line 166.


Package natbib Warning: Citation `Habib2017-sn' on page 4 undefined on
input li
ne 170.


Package natbib Warning: Citation `Satija2015-nn' on page 4 undefined on
input l
ine 170.


Package natbib Warning: Citation `Butler2017-kv' on page 4 undefined on
input l
ine 170.


Package natbib Warning: Citation `Macosko2015-yd' on page 4 undefined on
input
line 174.


Package natbib Warning: Citation `Smith2017-xd' on page 4 undefined on
input li
ne 174.


Overfull \hbox (253.11394pt too wide) has occurred while \output is
active
[][]
 []

[4]

Package natbib Warning: Citation `Parekh2018-db' on page 5 undefined on
input l
ine 192.
```

```
Underfull \hbox (badness 10000) in paragraph at lines 197--201

 []

No file main.bbl.
Package epstopdf Info: Source file: <zUMI_pipeline.eps>
(epstopdf)                    date: 2018-04-22 10:19:55
(epstopdf)                    size: 10640774 bytes
(epstopdf)             Output file: <zUMI_pipeline-eps-converted-to.pdf>
(epstopdf)                    date: 2018-04-22 10:20:17
(epstopdf)                    size: 1667698 bytes
(epstopdf)             Command: <repstopdf --outfile=zUMI_pipeline-eps-
converte
d-to.pdf zUMI_pipeline.eps>
(epstopdf)             \includegraphics on input line 225.
Package epstopdf Info: Output file is already uptodate.
<zUMI_pipeline-eps-converted-to.pdf, id=148, 2137.9875pt x 723.70375pt>
File: zUMI_pipeline-eps-converted-to.pdf Graphic file (type pdf)
<use zUMI_pipeline-eps-converted-to.pdf>
Package pdftex.def Info: zUMI_pipeline-eps-converted-to.pdf used on input
line
225.
(pdftex.def)             Requested size: 463.81499pt x 156.99571pt.
LaTeX Font Info:    Font shape `T1/Merriweather-OsF/m/n' will be
(Font)              scaled to size 6.0pt on input line 227.
LaTeX Font Info:    Font shape `T1/Merriweather-OsF/b/n' will be
(Font)              scaled to size 6.0pt on input line 227.
LaTeX Font Info:    Font shape `T1/Merriweather-OsF/m/it' will be
(Font)              scaled to size 6.0pt on input line 227.
<figure2.pdf, id=149, 794.97pt x 614.295pt>
File: figure2.pdf Graphic file (type pdf)
<use figure2.pdf>
Package pdftex.def Info: figure2.pdf used on input line 234.
(pdftex.def)             Requested size: 244.11394pt x 188.62979pt.
<zUMIsFigure_utilities.pdf, id=150, 867.24pt x 476.96884pt>
File: zUMIsFigure_utilities.pdf Graphic file (type pdf)
<use zUMIsFigure_utilities.pdf>
Package pdftex.def Info: zUMIsFigure_utilities.pdf used on input line
242.
(pdftex.def)             Requested size: 439.4021pt x 241.66429pt.
<figure4v3.pdf, id=151, 939.51pt x 505.89pt>
File: figure4v3.pdf Graphic file (type pdf)
<use figure4v3.pdf>
Package pdftex.def Info: figure4v3.pdf used on input line 251.
(pdftex.def)             Requested size: 488.22787pt x 262.88676pt.

Package natbib Warning: Citation `Habib2017-sn' on page 5 undefined on
input li
ne 257.


Package natbib Warning: Citation `Satija2015-nn' on page 5 undefined on
input l
```

```
ine 257.


Package natbib Warning: Citation `Butler2017-kv' on page 5 undefined on
input l
ine 257.


Package natbib Warning: Citation `Habib2017-sn' on page 5 undefined on
input li
ne 257.


Package natbib Warning: Citation `Habib2017-sn' on page 5 undefined on
input li
ne 257.

LaTeX Font Info:    External font `cmex10' loaded for size
(Font)              <6> on input line 257.
LaTeX Font Info:    Font shape `T1/MerriweatherSans-TLF/m/n' will be
(Font)              scaled to size 6.0pt on input line 257.

Underfull \hbox (badness 10000) in paragraph at lines 279--279
[]|\T1/Merriweather-OsF/m/n/7 Open
 []


Underfull \hbox (badness 10000) in paragraph at lines 279--279
[]|\T1/Merriweather-OsF/m/n/7 UMI col-
 []


Underfull \hbox (badness 6775) in paragraph at lines 279--279
[]|\T1/Merriweather-OsF/m/n/7 BC de-tec-
 []


Underfull \hbox (badness 10000) in paragraph at lines 279--279
[]|\T1/Merriweather-OsF/m/n/7 Down-
 []


Overfull \hbox (2.83214pt too wide) in paragraph at lines 279--279
[]|\T1/Merriweather-OsF/m/n/7 Compatible
 []


Underfull \hbox (badness 10000) in paragraph at lines 279--279
\T1/Merriweather-OsF/m/n/7 UMI li-
 []


Underfull \hbox (badness 10000) in paragraph at lines 279--279
\T1/Merriweather-OsF/m/n/7 brary
```

```
 []


Underfull \hbox (badness 10000) in paragraph at lines 279--279
[]|\T1/Merriweather-OsF/m/n/7 Cell
 []


Package natbib Warning: Citation `Zheng2017-zx' on page 5 undefined on
input li
ne 279.


Underfull \hbox (badness 10000) in paragraph at lines 279--279
[]|\T1/Merriweather-OsF/m/n/7 Hamming
 []


Package natbib Warning: Citation `Zheng2017-zx' on page 5 undefined on
input li
ne 279.


Package natbib Warning: Citation `Hashimshony2016-nk' on page 5 undefined
on in
put line 279.


Underfull \hbox (badness 10000) in paragraph at lines 279--279
[]|\T1/Merriweather-OsF/m/n/7 identity
 []


Package natbib Warning: Citation `Grun2014-ez' on page 5 undefined on
input lin
e 279.


Package natbib Warning: Citation `Hashimshony2016-nk' on page 5 undefined
on in
put line 279.


Package natbib Warning: Citation `Petukhov2017-ui' on page 5 undefined on
input
 line 279.


Underfull \hbox (badness 10000) in paragraph at lines 279--279
[]|\T1/Merriweather-OsF/m/n/7 TopHat2
 []


Underfull \hbox (badness 10000) in paragraph at lines 279--279
```

```
[]|\T1/Merriweather-OsF/m/n/7 WL,top-
 []


Package natbib Warning: Citation `Macosko2015-yd' on page 5 undefined on 
input
line 279.


Package natbib Warning: Citation `Klein2015-mj' on page 5 undefined on 
input li
ne 279.


Package natbib Warning: Citation `Zheng2017-zx' on page 5 undefined on 
input li
ne 279.


Underfull \hbox (badness 10000) in paragraph at lines 279--279
[]|\T1/Merriweather-OsF/m/n/7 Drop-seq-
 []


Package natbib Warning: Citation `Macosko2015-yd' on page 5 undefined on 
input
line 279.


Underfull \hbox (badness 10000) in paragraph at lines 279--279
[]|\T1/Merriweather-OsF/m/n/7 Hamming
 []


Package natbib Warning: Citation `Macosko2015-yd' on page 5 undefined on 
input
line 279.


Package natbib Warning: Citation `Soumillon2014-hd' on page 5 undefined 
on inpu
t line 279.


Package natbib Warning: Citation `Hashimshony2016-nk' on page 5 undefined 
on in
put line 279.


Package natbib Warning: Citation `Tian2017-eh' on page 5 undefined on 
input lin
e 279.
```

```
Underfull \hbox (badness 10000) in paragraph at lines 279--279
[]|\T1/Merriweather-OsF/m/n/7 Hamming
 []


Package natbib Warning: Citation `Grun2014-ez' on page 5 undefined on
input lin
e 279.


Package natbib Warning: Citation `Jaitin2014-na' on page 5 undefined on
input l
ine 279.


Package natbib Warning: Citation `Soumillon2014-hd' on page 5 undefined
on inpu
t line 279.


Package natbib Warning: Citation `Macosko2015-yd' on page 5 undefined on
input
line 279.


Package natbib Warning: Citation `Svensson2017-gy' on page 5 undefined on
input
 line 279.


Underfull \hbox (badness 10000) in paragraph at lines 279--279
[]|\T1/Merriweather-OsF/m/n/7 WL,top-
 []


Package natbib Warning: Citation `Soumillon2014-hd' on page 5 undefined
on inpu
t line 279.


Package natbib Warning: Citation `Grun2014-ez' on page 5 undefined on
input lin
e 279.


Package natbib Warning: Citation `Islam2014-kv' on page 5 undefined on
input li
ne 279.


Package natbib Warning: Citation `Jaitin2014-na' on page 5 undefined on
input l
ine 279.
```

```
Package natbib Warning: Citation `Macosko2015-yd' on page 5 undefined on
input
line 279.


Package natbib Warning: Citation `Klein2015-mj' on page 5 undefined on
input li
ne 279.


Package natbib Warning: Citation `Zheng2017-zx' on page 5 undefined on
input li
ne 279.


Package natbib Warning: Citation `Smith2017-xd' on page 5 undefined on
input li
ne 279.


Underfull \hbox (badness 10000) in paragraph at lines 279--279
[]|\T1/Merriweather-OsF/m/n/7 network-
 []


Package natbib Warning: Citation `Soumillon2014-hd' on page 5 undefined
on inpu
t line 279.


Package natbib Warning: Citation `Klein2015-mj' on page 5 undefined on
input li
ne 279.


Underfull \hbox (badness 10000) in paragraph at lines 279--279
[]|\T1/Merriweather-OsF/m/n/7 Hamming
 []


Underfull \hbox (badness 10000) in paragraph at lines 279--279
[]|\T1/Merriweather-OsF/m/n/7 A,WL,top-
 []


Package natbib Warning: Citation `Soumillon2014-hd' on page 5 undefined
on inpu
t line 279.


Package natbib Warning: Citation `Grun2014-ez' on page 5 undefined on
input lin
e 279.
```

```
Package natbib Warning: Citation `Islam2014-kv' on page 5 undefined on
input li
ne 279.


Package natbib Warning: Citation `Jaitin2014-na' on page 5 undefined on
input l
ine 279.


Package natbib Warning: Citation `Macosko2015-yd' on page 5 undefined on
input
line 279.


Package natbib Warning: Citation `Hashimshony2016-nk' on page 5 undefined
on in
put line 279.


Package natbib Warning: Citation `Hochgerner2017-wt' on page 5 undefined
on inp
ut line 279.


Package natbib Warning: Citation `Habib2017-sn' on page 5 undefined on
input li
ne 279.


Package natbib Warning: Citation `Rosenberg2018-aj' on page 5 undefined
on inpu
t line 279.


Package natbib Warning: Citation `Zheng2017-zx' on page 5 undefined on
input li
ne 279.


LaTeX Warning: `h' float specifier changed to `ht'.


AED: lastpage setting LastPage

Overfull \hbox (253.11394pt too wide) has occurred while \output is
active
[][]
 []

[5
```

```
]
Overfull \hbox (253.11394pt too wide) has occurred while \output is
active
[][]
 []

[6 <./zUMI_pipeline-eps-converted-to.pdf> <./figure2.pdf>]
Overfull \hbox (253.11394pt too wide) has occurred while \output is
active
[][]
 []

[7 <./zUMIsFigure_utilities.pdf>]
Overfull \hbox (253.11394pt too wide) has occurred while \output is
active
[][]
 []

[8 <./figure4v3.pdf>]

Package natbib Warning: There were undefined citations.

Package atveryend Info: Empty hook `BeforeClearDocument' on input line
283.
Package atveryend Info: Empty hook `AfterLastShipout' on input line 283.
(./main.aux)
Package atveryend Info: Executing hook `AtVeryEndDocument' on input line
283.
Package atveryend Info: Executing hook `AtEndAfterFileList' on input line
283.
Package rerunfilecheck Info: File `main.out' has not changed.
(rerunfilecheck)             Checksum:
2535D27E612411330F05046A086B7C4B;1574.

LaTeX Font Warning: Size substitutions with differences
(Font)              up to 1.0pt have occurred.

 )
Here is how much of TeX's memory you used:
 24718 strings out of 493027
 457134 string characters out of 6137679
 620786 words of memory out of 5000000
 27762 multiletter control sequences out of 15000+600000
 68206 words of font info for 205 fonts, out of 8000000 for 9000
 1141 hyphenation exceptions out of 8191
 64i,12n,102p,10407b,865s stack positions out of
5000i,500n,10000p,200000b,80000s
{c:/TeXLive/2015/texmf-dist/fonts/enc/dvips/lm/lm-
ec.enc}{c:/TeXLive/2015/tex
mf-
dist/fonts/enc/dvips/merriweather/mwth_jnnjab.enc}{c:/TeXLive/2015/texmf-
dis
t/fonts/enc/dvips/merriweather/mwth_ywgpba.enc}<c:/TeXLive/2015/texmf-
dist/font
```

```
s/type1/sorkin/merriweather/Merriweather-Bold.pfb><c:/TeXLive/2015/texmf-
dist/f
onts/type1/sorkin/merriweather/Merriweather-
BoldIt.pfb><c:/TeXLive/2015/texmf-d
ist/fonts/type1/sorkin/merriweather/Merriweather-
Italic.pfb><c:/TeXLive/2015/te
xmf-dist/fonts/type1/sorkin/merriweather/Merriweather-
Regular.pfb><c:/TeXLive/2
015/texmf-
dist/fonts/type1/public/amsfonts/cm/cmsy7.pfb><c:/TeXLive/2015/texmf-
dist/fonts/type1/public/amsfonts/euler/eusm7.pfb><c:/TeXLive/2015/texmf-
dist/fo
nts/type1/public/lm/lmtt8.pfb>
Output written on main.pdf (8 pages, 4895790 bytes).
PDF statistics:
 301 PDF objects out of 1000 (max. 8388607)
 234 compressed objects within 3 object streams
 38 named destinations out of 1000 (max. 500000)
 51909 words of extra memory for PDF output out of 61914 (max. 10000000)
```

Reference PDF

Click here to access/download
Supplementary Material
zumis_fast_flexible.pdf
