# Supplementary material for: zUMIs - A fast and flexible pipeline to process RNA sequencing data with UMIs
Source: Gigascience. 2018 May 26;7(6):giy059. doi: 10.1093/gigascience/giy059 (PMC6007394; doi:10.1093/gigascience/giy059)
Supplement: Response_to_Reviewer_Comments_Original_Submission.pdf [file giy059_response_to_reviewer_comments_original_submission.pdf]

Author's Response To Reviewer Comments

Close

In particular, both reviewers feel that some of your results that have been achieved by simulation need to be backed up with an analysis of real data (reviewer 1, #2; reviewer 2, #6). I also agree with reviewer 2 that it is important to compare the performance of (parts of) your pipeline with existing tools that perform steps in the zUMI pipeline.

AUTHOR RESPONSE:
Thank you for the useful comments. We have now backed up the comparison of UMI collapsing approaches by analysis of real data. To this end, we have added descriptive statistics plots in a new Figure 2. This data also shows how well gene expression estimates correspond between published pipelines and zUMIs. Furthermore, we have added a Table showing presence or absence of important features in existing tools and zUMIs.

Reviewer 2's comments regarding technical and biological sources of variation (#2 in the report) is another crucial point that needs careful consideration when you are preparing a revised submission.

AUTHOR RESPONSE:
We have added a deeper analysis of the DroNc-seq dataset to show more in detail the biological relevance of adding Intronic counts (see detailed answer in the Response to Reviewers) below. Additionally, we show that the Intronic counts are not artifacts by sampling fake random Intronic reads and showing that this actually decreases cluster resolution (for more details see response to Reviewer 1, comment 3).

It is important that the description of your methods allows full reproducibility - please include missing details, as outlined by our reviewers.

AUTHOR RESPONSE:
We have added a detailed Methods section in the paper to carefully describe the datasets and analysis strategies.

Reviewer #1: Parekh and coworkers introduce a pipeline to process high throughput scRNA-seq data consisting of cell barcodes and UMIs. This is an open-source software that also supports features such as reads downsampling and Intron counting - the latter is important for single nucleus RNA-seq data. Overall, this is a useful study and I would like to support its publication, but the current manuscript could greatly benefit with additional biological analysis (related to Fig 4) that would prompt users to take notice. I would like to support its publication, contingent on the authors addressing the following comments.

1. The pipeline appears to collapse only identical UMIs. We have found in our experience that this can lead to overcounting of transcripts, and that it is necessary to collapse UMIs mapping to the

same gene in the same cell within an edit distance of 1. I would be curious to see how this impacts the number of transcripts detected per cell.

AUTHOR RESPONSE:
zUMIs now also offers the option to collapse UMIs based on Hamming distance and add a plot comparing the number of UMIs/cell for 4 different approaches (updated Figure 1). We also extended the text accordingly:

“Per default, we only collapse UMIs by sequence identity. If there is a risk that a large proportion of UMIs remains under-collapsed due to sequence errors, zUMIs provides the option to collapse UMIs within a given Hamming distance. We compare the two zUMIs UMI-collapsing options to the recommended directional adjacency approach implemented in UMI-tools [15], using our in-house example dataset (see Methods). zUMIs identity collapsing yields nearly identical UMI counts per cell as UMI-tools, while Hamming distance yields increasingly fewer UMIs/cell with increasing sequencing depth (Figure 2C). Smith et al. [15] suggest that edit distance collapsing without considering the relative frequencies of UMIs might indeed overreach and over-collapse the UMIs. We suspect that this is indeed what happens in our example data, where we find that gene-wise dispersion estimates appear suspiciously truncated as expected if several counts are unduly reduced to one, the minimal number after collapsing (Figure 2D).
However, note that the above described differences are minor. By and large, there is good agreement between UMI counts obtained by UMI-tools [15], the Drop-seq pipeline [24] and zUMIs. The correlation between gene-wise counts of the same cell is > 0.99 for all comparisons (Figure 2B). In light of this, we would consider the > 3 times higher processing speed of zUMIs a decisive advantage (Figure 2A)“
---

2. The authors use simulations to describe the impact of Intron counting on differential expression. I would instead like to see this on real data. In particular I would like to see examples of "before/after" plots (e.g. violin plots/heatmaps) of specific genes that (1) were called out as differentially expressed (DE) but no longer are once introns are incorporated, (2) the reverse of (1), and (3) those that remain DE but with significantly different statistical significances.

AUTHOR RESPONSE:
To analyse real data we use the DroNc-seq data from Habib et al. (2017). We analyse the log2 fold changes (LFC) for the groups that were split up more when using Exon+Intron counting (see the new Figure 4F) and we added a description of our findings to the main text.

“Following the Seurat pipeline to cluster cells [30, 31], we find that using Exon+Intron counts discriminates 28 clusters, while we could only discriminate 19 clusters using Exon counts (Figure 4A+B). We then continue to further characterize the 7 clusters that were further subdivided by the addition of Intron counts (Figure 4D). First, we identify differentially expressed (DE) genes between the newly formed clusters. If we count only Exon reads there appears on average only 10 genes to be DE between the sub-groups, while Exon+Intron counting yields ~10x more DE genes, thus corroborating the signal found with clustering. The log2-fold changes estimated for the additional DE genes estimated with either counting strategy are generally in good agreement, especially large

log2-fold changes are detected with both Exon and Exon+Intron counting (Figure 4F).“
---

3. I am also not convinced of the result claiming more clusters when introns are included. What is the evidence that these clusters are not spurious? The detection of additional clusters is not evidence enough that these are real. It would be useful to show a heatmap demonstrating that there is true, biologically significant differential expression between the novel clusters detected by the Intron counting.

AUTHOR RESPONSE:
We now added plots with the numbers of DE genes distinguishing the newly split clusters for both Exon+Intron as well as Exon counting (Figure 4D). Note that with the number of DE genes also the more informative marker genes such as the example in Figure 4E are detected. Thus, even though we do not fully understand the biological meaning of the more fine grained clusters, we are confident that they are indeed based on a biological signal from the RNA-seq data. Additionally, we demonstrate this, by sampling randomly from the distribution of intronic counts and adding to the exonic counts. The resulting extra-noise in fact leads to a lower number of clusters detected: 19 Exon 28 Intron+Exon 7 fake Intron+Exon (see plot in attached “Additional File 1”).
---

4. For completeness, I would like if the authors could include a section comparing their "exon-counts" matrix with the count matrix produced by either the cellranger or Drop-seq-tools for datasets that have been classically analyzed by the latter methods. This would produce some confidence in the base reproducibility of the methods. If on the other hand, zUMIs produces a different Exon count matrix, then the authors must explain why this is the case.

AUTHOR RESPONSE:
Unfortunately, we could not run the cellranger pipeline on our example dataset, because it does not allow to freely specify cell barcodes. Instead we compare to the Drop-seq and the UMI-tools pipelines. We generally find a high correlation between the number of UMIs per gene detected in a cell. The slight discrepancies between zUMIs and the Drop-seq-tools are due to how reads are associated with genes. For example zUMIs does not count ambiguously mapped reads, i.e. reads that overlap with multiple genes, while Drop-seq counts them for all genes.
UMI-tools on the other hand also uses featureCounts for read association, however their recommended method to collapse UMIs by directional adjacency with edit distance 1 differs from the options in zUMIs. Here, our newly added feature of collapsing UMIs Hamming distance yields as expected the most similar counts.
These results are now included in Figure 2C.
---

5. In Figure 4, the authors show to show a confusion matrix to compare how clusters in A map to clusters in B. Also for those clusters that multi-map (i.e. those resolved by intron-Exon mapping but not by exon-mapping alone), is there biologically meaningful differential expression? Some examples of specific cell types and their gene expression differences in A vs B would be very informative.

AUTHOR RESPONSE:
We added an example for a subsplit of a mainly GABAergic cluster that also has significantly DE

Marker gene for Pvalb GABAergic neurons when considering Intron+Exon counts in Figure 4E and discuss this in the main text:

"Having a closer look at cluster 7, it was split into a bigger (7) and a smaller cluster (24) using exon+intron counting (Figure 4A-C), we find one marker gene (Il1rapl2) to be DE between the subclusters using Exon+Intron counting, while Il1rapl2 had only spurious counts using Exon counts. Il1rapl2 is a marker for transcriptomic subtypes of GABAergic Pvalb-type Neurons, suggesting that the split of cluster 7 might be biological meaningful (Figure 4E)."

Reviewer #2: Review of "zUMIs - A fast and flexible pipeline to process RNA sequencing data with UMIs"

Summary:
Parekh et al. describe a computational pipeline to preprocess single-cell RNA-seq data that contains UMIs and cell barcodes. The main components of the pipeline include sequence quality filtering of UMIs and barcodes, a wrapper to call the mapping software STAR, selection of cell barcodes, and downsampling of reads to lower library size. While other tools exist that perform all of these steps either all together or individually for one or more platforms, the novelty of zUMIs is that it performs all of these steps at once for data from any UMI platform. Such a tool would likely be useful for the single-cell community, however many methodological details are missing. In addition the manuscript could benefit from additional comparison to existing tools.

The authors also argue that in general quantification of gene expression should incorporate intron-mapping reads, a task which is enabled by the use of their software. However, I have reservations about the evidence upon which this conclusion is based.

AUTHOR RESPONSE:
We want to clarify that we do not wish to claim that counting introns is a good idea in general. However, we argue that for extremely sparse datasets such as generated by single nuclei sequencing, having Intron counts is better than losing even more genes. We hope that we could make this clearer in the text, as such:

"Furthermore, we think that although noisy, the large number of additionally detected genes makes Exon+Intron counting worthwhile for extremely sparse data."

I have identified several issues that the authors should address in order to improve the manuscript, which are detailed below and divided into major (of critical importance) and minor (to improve clarity) categories.

Major Comments:
1. Methodological details are missing throughout. The method should be described more completely and clearly, and any analyses or comparisons should be made reproducible. For example:
* What differential expression method was used in the simulation study to compare UMItools and zUMI?
* What options were used with powsimR in the simulation study?
* How is the k-dimensional multivariate normal distribution fit in the cell barcode selection step?
* How is k determined in the cell barcode selection step?

* How was data simulated for the Intron evaluation?
* What options were used in applying the Seurat pipeline to cluster cells?

AUTHOR RESPONSE:
We added a methods section (Page:3-4) that includes subsections for (1) data generation of the HEK dataset as well as data processing of other used datasets, (2) the powsimR simulations and (3) the use of the Seurat pipeline. The passage about the Cell-Barcode selection was changed in the main text (Page:2). We hope to have made our barcode selection clearer in the main text.

"To this end, we fit a k-dimensional multivariate normal distribution using the R-package mclust [25, 26] for the number of reads/BC, where k is empirically determined by mclust via the Bayesian Information Criterion (BIC). We reason that only the kth normal distribution with the largest mean contains barcodes that identify reads originating from intact cells."
---

2. One major conclusion of the paper is that incorporation of intron-mapping reads significantly improves cluster resolution. It is perhaps not surprising that including the Intron counts results in a higher mean number of genes detected, but the authors conclude that since more clusters are also found that this means the additional reads are biologically meaningful. Unfortunately, the authors have not provided any evidence that this is the case. The fact that more clusters are seen says nothing about the difference between technical and biological sources of variation. If these additional clusters also corresponded to some independently measured biological covariate, the argument would have basis.

AUTHOR RESPONSE:
While we do not wish to claim that counting of intron-mapping reads is recommended in all cases of scRNA-seq, we do think it is valid and helpful for extremely sparse datasets such as the DroNc-seq data from Habib et al. (2017). We now provide detailed analyses of differences between newly formed subclusters using Exon+Intron counting. We find not only more genes, but also more significantly differentially expressed genes between subclusters when using Exon+Intron UMI data (Figure 4D). Furthermore, log2 fold changes (LFC) for the groups that were split up more when using Exon+Intron counting corresponded well to the Exon-only LFC (see the new Figure 4F). Additionally, we illustrate the biological relevance of subclusters found with Exon+Intron data by the example of the transcriptomic subtypes of GABAergic Pvalb-type Neurons marked by Il1rapl2 expression. We have added this evidence to the 'Intron Counting' section and included methodological details in the appropriate Methods sections.
Lastly, we have excluded the possibility of Intron-mapping reads being spurious by sampling fake intronic reads and attempting cluster identification (see response to Reviewer 1, point 3).
---

3. Many central conclusions of the article were made based on an analysis of a dataset of 96 cells that is never described. It is referred to as "the HEK dataset" throughout the manuscript, but no citation, details of data generation, or description of the experimental design is given.

AUTHOR RESPONSE:
We added this information to the new Method section (Page:3-4).
---

4. Several open-source tools exist that perform many of the steps in the zUMI pipeline [1, 2, 3]. It would be nice to see how these perform in comparison to zUMI.

AUTHOR RESPONSE:
While several tools exist that can perform some of the steps of the zUMIs pipeline, none of them provides a comprehensive combination as zUMIs. We have added a Table to compare available features of six other pipelines geared towards scRNA-seq data with UMIs. The tool “UMI-Reducer” with reference [2] suggested by the reviewer was omitted because it seemed like a tool geared towards one specific application outside of single-cell RNA-seq. Furthermore, “UMI-Reducer” only de-duplicates UMIs with the same mapping position, which would be inappropriate for scRNA-seq protocols that fragment after preamplification, such as SCRB-seq.
Furthermore, we added a comparison of the count-tables produced by zUMIs, Drop-seq-tools and UMI-tools and generally find very good correspondence (see response to Reviewer 1).
---

5. The conclusion that a UMI distance filter (using UMI-tools) is unnecessary is only based on a single simulated dataset of up to 90 cells per condition. It is also based on a single metric (power to identify differentially expressed genes in simulated data). If we are only interested in differential expression analyses, this might be a reasonable metric. However to be widely applicable to the analysis of single cell RNA-seq, the authors should consider additional metrics such as replicate reproducibility, number of detected genes, etc. The authors should also consider additional datasets.

AUTHOR RESPONSE:
We substantially extended our comparison of different UMI-collapsing method. In Fig. 2 B,C, we also compare the correlation of gene expression values and numbers of detected UMIs per cell between various different filtering methods and find that there is generally a high consensus among all UMI collapsing methods in our HEK example dataset. An analysis of the DroNc-seq data gave basically the same results (see plot in attached “Additional File 1”).
Furthermore, we added the possibility to collapse UMIs with a specified Hamming-distance to zUMIs, giving users more choice over UMI filtering. All these new analysis are also described in the section “Transcript Counting” of the main text.
---

6. It is not clear how the simulation parameters in the comparison to UMI-tools directly relate to the UMI quantification. Specifically, estimating the mean and dispersion of the processed data and then using these as the basis for a simulated dataset seems pretty far removed from the observed UMI counts. The authors should also investigate differences in differential expression analysis of the actual data (not simulated data). They could also generate a simulated null comparison by randomly permuting sample labels. The same comments hold for the second simulation (evaluating Intron count inclusion).

AUTHOR RESPONSE:
We removed the simulations from the description of UMI-collapsing methods and focus our reporting on the descriptive statistics suggested by the reviewer (Figure 2 & section “Transcript counting”).

Minor Comments:
1. The results of the simulation evaluating Intron usage are summarized broadly in the text, but the specific results are not shown. For example what does "power to detect differentially expressed genes was similar for the Exon and Exon+Intron counts" mean? How similar? What were the values?

AUTHOR RESPONSE:
This is now better described in the main text (Page 3 Passage:Intron Counting) along with specific settings for the powsimR package listed in the method section. Additionally, power simulation results are shown in Figure 4 with the true positive rate (TPR) and false discovery rate (FDR) shown for 5 stratas of gene expression (Figure 4G). Furthermore, we display the number of genes per stratum for Exon and Exon+Intron counting (Figure 4H).
---

2. The pipeline requires the user to specify many parameters for each step, however the implementation is run with one command. This means that if a user wants to change a single parameter in one of the later steps, they would still have to rerun the entire pipeline, wasting time and computational resources. It would be useful if the pipeline could alternatively be run as a series of individual steps so that the same exact steps don't need to be carried out multiple times in these situations.

AUTHOR RESPONSE:
This feature is implemented as “-w” option. One can invoke zUMIs at any step, eg to just re-run the counting of gene expression the user can give “-w counting”.
---

3. In the cell barcode selection step, the authors state that they remove "all barcodes that fall in the lower 1% tail of this distribution." What is the justification for this? What does this correspond to in practice? This threshold should also be denoted in Figure 3A.

AUTHOR RESPONSE:
The blue line in figure 3A corresponds to the calculated read cut-off. The normal distribution identified by mclust with the highest mean number of reads contains actual cell barcodes. Thus, setting the read cut-off to the lower 1% of this distribution is an empirical value that gives good correspondence to the known cell-barcodes for the HEK dataset (cut-off value: 52634 reads/barcode) and gave similarly good results for the DroNc-seq data analysed here. Still, in practice we recommend to always look at the elbow-plots output by zUMIs (Figure 3B). This will show whether our empirical cut-off was also valid for the dataset at hand.
---

4. What are the practical guidelines for downsampling? How should it be used in practice to normalize for sequencing depth?

AUTHOR RESPONSE:
We found the downsampling function extremely useful for method comparisons as we showed in our previous study (Ziegenhain et al. 2017). This also allows to evaluate whether the single cell libraries were sequenced to saturation (Figure 3D). For normalization purposes, the built-in MAD

cut-offs as indicated by the dashed red lines in Figure 3C should be sufficient.
---

5. In the documentation online, section on cell barcode selection (here: https://github.com/sdparekh/zUMIs/wiki/Cell-barcodes-selection), Figure A is contradictory to Figure 3A in the manuscript. Specifically, the online documentation says "cells left to the blue line are selected" and the manuscript says "cell barcodes with reads above the blue line are selected."

AUTHOR RESPONSE:
This was indeed a mistake and we corrected it on GitHub.
---

6. As a main advantage of zUMIs is the ability to apply on any UMI platform, the documentation should clearly state how to use the software in each case. Currently, this is unclear, as for example in the case of the "-c" option the wiki on GitHub (https://github.com/sdparekh/zUMIs/wiki/Usage) states that "For STRT-seq/InDrops give this as 1-n where n is your first cell barcode(-f) length." But it also states in the very next line "For InDrops give this as 1-n where n is the total length of cell barcode(e.g. 1-22)," which is contradictory to what the previous line states about InDrops.

AUTHOR RESPONSE:
This was indeed a mistake and we corrected it on GitHub.

References:
[1] Luyi Tian, Shian Su, Daniela Amann-Zalcenstein, Christine Biben, Shalin H. Naik, Matthew E. Ritchie. scPipe: a flexible data preprocessing pipeline for single-cell RNA-sequencing data. bioRxiv 175927; doi: https://doi.org/10.1101/175927

[2] Serghei Mangul, Sarah Van Driesche, Lana S. Martin, Kelsey C. Martin, Eleazar Eskin. UMI-Reducer: Collapsing duplicate sequencing reads via Unique Molecular Identifiers. bioRxiv 103267; doi: https://doi.org/10.1101/103267

[3] Viktor Petukhov, Jimin Guo, Ninib Baryawno, Nicolas Severe, David Scadden, Maria G. Samsonova, Peter V. Kharchenko. Accurate estimation of molecular counts in droplet-based single-cell RNA-seq experiments. bioRxiv 171496; doi: https://doi.org/10.1101/171496

Close
