# Supplementary material for: zUMIs - A fast and flexible pipeline to process RNA sequencing data with UMIs
Source: Gigascience. 2018 May 26;7(6):giy059. doi: 10.1093/gigascience/giy059 (PMC6007394; doi:10.1093/gigascience/giy059)
Supplement: Response_to_Reviewer_Comments_Revision_1.pdf [file giy059_response_to_reviewer_comments_revision_1.pdf]

Author's Response To Reviewer Comments

Close

Note: For better readability, we only include the reviewer reports with remaining concerns in the point by point response below.

Reviewer reports:
Reviewer #2: Review of Revised version R1 of "zUMIs - A fast and flexible pipeline to process RNA sequencing data with UMIs"

Parekh et al. have partially addressed my concerns, however there remain unresolved issues that have critical importance to the conclusions of the manuscript. In particular, although the authors have included a Methods section with the revision, some methodological details for reproducibility are still missing. In addition, although the authors have compared one aspect of the pipeline to existing tools, other steps of the pipeline that are also carried out by other tools are not compared. Finally, the biological relevance of the differences in clustering with and without introns is still not convincingly demonstrated. These remaining concerns are detailed below (new responses marked by ***).

AUTHOR RESPONSE:
We want to clarify that we do not wish to claim that counting introns is a good idea in general. However, we argue that for extremely sparse datasets such as generated by single nuclei sequencing, having Intron counts is better than losing even more genes. We hope that we could make this clearer in the text, as such:

"Furthermore, we think that although noisy, the large number of additionally detected genes makes Exon+Intron counting worthwhile for extremely sparse data."

***
REVIEWER RESPONSE:
Thank you for the clarification. However, this recommendation remains vague. It would be useful to define what is meant by "extremely sparse data". It may not be clear to the single-cell community, since all single-cell data is quite sparse by nature. But it seems this recommendation is focused on a particular subset of protocols (DroNc-seq)? Please clarify.
***

AUTHOR RESPONSE 2:
We have revised this text passage to be more specific, so readers know when to consider Exon+Intron counting. Our recommendation is now differentiated between nucleus sequencing that is enriched in intronic sequences because of nascent mRNA molecules and low coverage sparse data generated in high throughput applications.

“Furthermore, we think that, although potentially noisy, the large number of additionally detected genes makes Exon+Intron counting worthwhile, especially for single- nuclei sequencing techniques that are enriched for nuclear nascent RNA transcripts, such as DroNc-seq [12]. Additionally, Exon+Intron counting may help extracting as much information as possible from low coverage data

as generated in the context of high-throughput cell atlas efforts (eg 10,000- 20,000 reads/cell [37, 38]. Lastly, users should always exclude the possibility of intronic reads stemming from genomic DNA contamination in the library preparation by confirming low intergenic mapping fractions using the statistics output provided by zUMIs."

I have identified several issues that the authors should address in order to improve the manuscript, which are detailed below and divided into major (of critical importance) and minor (to improve clarity) categories.

Major Comments:
1. Methodological details are missing throughout. The method should be described more completely and clearly, and any analyses or comparisons should be made reproducible. For example:
* What differential expression method was used in the simulation study to compare UMItools and zUMI?
* What options were used with powsimR in the simulation study?
* How is the k-dimensional multivariate normal distribution fit in the cell barcode selection step?
* How is k determined in the cell barcode selection step?
* How was data simulated for the Intron evaluation?
* What options were used in applying the Seurat pipeline to cluster cells?

AUTHOR RESPONSE:
We added a methods section (Page:3-4) that includes subsections for (1) data generation of the HEK dataset as well as data processing of other used datasets, (2) the powsimR simulations and (3) the use of the Seurat pipeline. The passage about the Cell-Barcode selection was changed in the main text (Page:2). We hope to have made our barcode selection clearer in the main text.

"To this end, we fit a k-dimensional multivariate normal distribution using the R-package mclust [25, 26] for the number of reads/BC, where k is empirically determined by mclust via the Bayesian Information Criterion (BIC). We reason that only the kth normal distribution with the largest mean contains barcodes that identify reads originating from intact cells."

***
REVIEWER RESPONSE:
The authors added a Methods section. Most of the details seem to have been added, but there are still some details that I have questions about. For example, how was the Intron-sampling experiment carried out (Figure 1 in Additional File 1)?
What is meant by "sufficiently many cells for DE analysis" (page 3)?
***

AUTHOR RESPONSE 2:
We now added a description of the Intron-Sampling to Clustering part of the Methods section. Sorry, for the confusion about the statement "sufficiently many cells ..." - In this analysis we included all genes that were detected, i.e. had at least one read mapped. We changed this sentence.
"For a fair comparison, we include all detected genes".
---

2. One major conclusion of the paper is that incorporation of intron-mapping reads significantly

improves cluster resolution. It is perhaps not surprising that including the Intron counts results in a higher mean number of genes detected, but the authors conclude that since more clusters are also found that this means the additional reads are biologically meaningful. Unfortunately, the authors have not provided any evidence that this is the case. The fact that more clusters are seen says nothing about the difference between technical and biological sources of variation. If these additional clusters also corresponded to some independently measured biological covariate, the argument would have basis.

AUTHOR RESPONSE:
While we do not wish to claim that counting of intron-mapping reads is recommended in all cases of scRNA-seq, we do think it is valid and helpful for extremely sparse datasets such as the DroNc-seq data from Habib et al. (2017). We now provide detailed analyses of differences between newly formed subclusters using Exon+Intron counting. We find not only more genes, but also more significantly differentially expressed genes between subclusters when using Exon+Intron UMI data (Figure 4D). Furthermore, log2 fold changes (LFC) for the groups that were split up more when using Exon+Intron counting corresponded well to the Exon-only LFC (see the new Figure 4F). Additionally, we illustrate the biological relevance of subclusters found with Exon+Intron data by the example of the transcriptomic subtypes of GABAergic Pvalb-type Neurons marked by Il1rapl2 expression. We have added this evidence to the 'Intron Counting' section and included methodological details in the appropriate
Methods sections.
Lastly, we have excluded the possibility of Intron-mapping reads being spurious by sampling fake intronic reads and attempting cluster identification (see response to Reviewer 1, point 3).

***
REVIEWER RESPONSE:
The authors have not fully addressed the concern of biologically meaningful clustering results. They highlight the example of a single gene, which is not convincing that the results are systematically meaningful. In main text they state that 5% of the additional genes found are marker genes, but no baseline is given to be able to judge if that is a significant result. What percentage of genes overall are marker genes? What percentage of DE genes by exon only are marker genes?

Furthermore while they have shown that there are more DE genes between sub-clusters with introns included (again this is not surprising since more genes are detected), this result could be influenced to a degree by the use of a model (limma-trend) that is not appropriate for sparse RNA-seq data (see also response to Major comment 6).

In addition, it seems that the figures or additional results presented in the Additional File (including the "sampling fake intronic reads") are never mentioned in the manuscript. To me, the sampling fake intronic reads analysis could be a valuable addition to supporting the conclusion that utilizing the intronic reads gives biologically meaningful clustering results (as long as the details of this simulation are realistic, but these details are currently not provided).

As an additional note, it is not clear what color represents in newly added Figures 4D, G, and H and this is not defined in the legend. It is also not clear what color represents in Figure 4A and B - some colors seem to map to the legend of 4C, but not all of them.
***

AUTHOR RESPONSE 2:
We extended the section where we cite a number of papers that utilize intron counts in the analysis of single cell RNA-seq and in particular single nuclei sequencing data. What information can be gained from intron counting is indeed a hot topic in the single cell community and even though we believe to contribute some evidence to support the notion that intron counts add biologically meaningful information, our paper is hardly the place where this issue can be fully resolved. However, the intron counting utility of zUMIs will facilitate research in this area and thus ultimately help other researchers to address this question.

Concerning the additionally detected marker genes: We do not expect any enrichment of marker genes when we use Exon+Intron counting and the 5% additional markers are roughly the level expected: 4 % of all detected Exon+Intron genes are also marker genes. However, detecting more of them gives us a better chance to later classify the cells.
We also agree that it is not surprising that we find more DE genes if we detect more genes overall. Including introns simply allows us to better detect present transcripts. On the contrary, we would be reluctant to recommend the inclusion of Introns if they would lead to a major shift in the expression profiles, i.e. many more DE- or marker genes than expected.

We now added the fake intron analysis to Figure 4 and also extended the methods section for Cluster Identification to describe our sampling:

"To illustrate that the additional clusters found by counting Exon+Intron reads are not spurious, we use Intron-only UMI-counts from the same data to add to the observed Exon only counts. More specifically, to each gene we add scran-sizeFactor corrected Intron counts from the same gene after permuting them across cells. We assessed the cluster numbers from 100 such permutations."

We now explain the color schemes of Figure 4 A,B &E in the figure legend and added a color legend for D,H &I (formerly D,G & H).
"Different shades of those clusters indicate that multiple clusters had the same major cell-type assigned."
---

4. Several open-source tools exist that perform many of the steps in the zUMI pipeline [1, 2, 3]. It would be nice to see how these perform in comparison to zUMI.

AUTHOR RESPONSE:
While several tools exist that can perform some of the steps of the zUMIs pipeline, none of them provides a comprehensive combination as zUMIs. We have added a Table to compare available features of six other pipelines geared towards scRNA-seq data with UMIs. The tool "UMI-Reducer" with reference [2] suggested by the reviewer was omitted because it seemed like a tool geared towards one specific application outside of single-cell RNA-seq. Furthermore, "UMI-Reducer" only de-duplicates UMIs with the same mapping position, which would be inappropriate for scRNA-seq protocols that fragment after preamplification, such as SCRB-seq.
Furthermore, we added a comparison of the count-tables produced by zUMIs, Drop-seq-tools and UMI-tools and generally find very good correspondence (see response to Reviewer 1).

***
REVIEWER RESPONSE

The authors have included a comparison to two other tools for the UMI-collapsing strategy. However, these other tools are not included in any other aspect. For example, how do other methods perform in cell barcode selection (Page 2)? How do other methods perform in running time (Figure 3E)?
***

AUTHOR RESPONSE 2:
The purpose of Figure 2 was never a comprehensive comparison of all UMI-collapsing tools, but it should merely provide perspective on the possible choices of UMI collapsing within zUMIs so that the user can make an informed choice given the run time and the added information.

A systematic comparison of runtimes among pipelines is beyond the scope of this Technical Note. More specifically, the input data vary widely and we would be hard pressed to find data-sets that can be fed into each of the pipelines, thus making it virtually impossible to provide a fair overall run-time comparison.

In fact zUMIs is the only tool that can handle data from all major UMI-based scRNA-seq library protocols (Table 1). Besides, we found that no other pipeline had the combination of processing featured that we found to be useful. This said, the main advantage of zUMIs is its unique combination of features and not the performance details of the separate steps.
That's why we feel that the comparison of features of 7 UMI pipelines that we provide in Table 1 is an adequate 'performance' evaluation. Table 1 now also includes a column detailing the various Barcode selection options.
---

6. It is not clear how the simulation parameters in the comparison to UMI-tools directly relate to the UMI quantification. Specifically, estimating the mean and dispersion of the processed data and then using these as the basis for a simulated dataset seems pretty far removed from the observed UMI counts. The authors should also investigate differences in differential expression analysis of the actual data (not simulated data). They could also generate a simulated null comparison by randomly permuting sample labels. The same comments hold for the second simulation (evaluating Intron count inclusion).

AUTHOR RESPONSE:
We removed the simulations from the description of UMI-collapsing methods and focus our reporting on the descriptive statistics suggested by the reviewer (Figure 2 & section "Transcript counting").

***
REVIEWER RESPONSE
The authors have removed one of the simulations from the manuscript, but the second simulation remains. Unfortunately, I still have concerns regarding the intron count inclusion simulation. In particular, now that the authors have added methodological details, they have used a differential expression method that was developed for bulk RNA-seq and is not appropriate for sparse single-cell RNA-seq data. It could be that the differential expression results are due to a poorly fitting model with more sparse exon-only data. The authors should use a method appropriate for sparse single-cell RNA-seq data, such as MAST (Finak et al. 2015, Genome Biology) or zingeR (Van den Berge et al. 2018, Genome Biology).

***

--- AUTHOR RESPONSE 2:
It is correct that limma-trend is a method developed for bulk RNA-seq. However, if applied correctly, bulk methods are also suitable for single cell data (Dal Molin, Baruzzo, and Di Camillo 2017). A recent, thorough comparison by Soneson & Robinson (Soneson and Robinson 2018) has shown that limma-trend is one of the best available methods for single-cell differential expression analysis. It outperformed many specialised single cell methods. Most notably, limma-trend was one of the most consistent DE tools across a wide range of data sets (see figure 5 of Soneson & Robinson). TPR and FDR were comparable to MAST (see figure 4), while limma-trend has a vastly superior runtime. Furthermore, many of the problems due to the sparsity of single cell data are introduced during normalization and we applied a normalisation method specifically developed for sparse gene expression data (scran) (Vallejos et al. 2017).

References:
Dal Molin, Alessandra, Giacomo Baruzzo, and Barbara Di Camillo. 2017. “Single-Cell RNA-Sequencing: Assessment of Differential Expression Analysis Methods.” Frontiers in Genetics 8 (May): 62. https://doi.org/10.3389/fgene.2017.00062.
Soneson, Charlotte, and Mark D. Robinson. 2018. “Bias, Robustness and Scalability in Single-Cell Differential Expression Analysis.” Nature Methods 15 (4): 255–61. https://doi.org/10.1038/nmeth.4612.
Vallejos, Catalina A., Davide Risso, Antonio Scialdone, Sandrine Dudoit, and John C. Marioni. 2017. “Normalizing Single-Cell RNA Sequencing Data: Challenges and Opportunities.” Nature Methods, May. https://doi.org/10.1038/nmeth.4292.
---

3. In the cell barcode selection step, the authors state that they remove "all barcodes that fall in the lower 1% tail of this distribution." What is the justification for this? What does this correspond to in practice? This threshold should also be denoted in Figure 3A.

AUTHOR RESPONSE:
The blue line in figure 3A corresponds to the calculated read cut-off. The normal distribution identified by mclust with the highest mean number of reads contains actual cell barcodes. Thus, setting the read cut-off to the lower 1% of this distribution is an empirical value that gives good correspondence to the known cell-barcodes for the HEK dataset (cut-off value: 52634 reads/barcode) and gave similarly good results for the DroNc-seq data analysed here. Still, in practice we recommend to always look at the elbow-plots output by zUMIs (Figure 3B). This will show whether our empirical cut-off was also valid for the dataset at hand.

***
REVIEWER RESPONSE
It would be useful to state your recommendation in the manuscript "to always look at the elbow-plots output by zUMIs (Figure 3B)" when setting the cut-off so that readers can benefit from this advice.
***

AUTHOR RESPONSE 2:
We have added this in the main text (page 2 section: Cell Barcode Selection).

Close
