# Supplementary material for: zUMIs - A fast and flexible pipeline to process RNA sequencing data with UMIs
Source: Gigascience. 2018 May 26;7(6):giy059. doi: 10.1093/gigascience/giy059 (PMC6007394; doi:10.1093/gigascience/giy059)
Supplement: Response_to_Reviewer_Comments_Revision_2.pdf [file giy059_response_to_reviewer_comments_revision_2.pdf]

Author's Response To Reviewer Comments

Close

In order to increase readability, we have included only the remaining questions of the reviewer

***
REVIEWER RESPONSE 2:
Please clarify "all detected genes". Is this all genes detected in Exon or Exon+Intron? If including all detected genes, then why are there different numbers of genes in each quantile in Figure 4H (top panel)? By definition, a quantile bin should contain roughly equal numbers of observations, but this does not seem to be the case even if the quantiles were defined on the Exon+Intron data (the larger quantile bins have more genes for Exon+Intron).
***

Author response:
We have amended the text to clarify this point as requested.
"For a fair comparison, we include all detected genes, which is equivalent to the number of genes detected with Exon+Intron counting and since we call a gene detected as soon as one count is associated, Exon counting is necessarily a subset of Exon+Intron."

***
REVIEWER RESPONSE 2:
Thanks for the clarification regarding enrichment of marker genes as opposed to simply detecting more genes in general. I appreciate the explanation given by the paragraph in the authors' response beginning with "Concerning the additionally detected marker genes:...". It would be transparent to include this explanation in the manuscript, since as it reads currently, it seems to imply an enrichment for marker genes when including introns.
***

Author response:
We agree with making the explanation as transparent as possible and have amended the text in this regard.
"Here, we cross-reference the gene list with marker genes for transcriptomic subtypes detected for major cell types of the mouse brain and find that ~5% of the additional genes are also marker genes, which corresponds well to the general frequency of marker genes among the detected genes (4%). In the same vein, we also detect proportionally more DE genes with Exon+Intron counting as compared to Exon counting. Thus including introns simply allows us to better detect present transcripts, while it leaves the proportions of interest unaltered."

Close
