# Supplementary material for: zUMIs - A fast and flexible pipeline to process RNA sequencing data with UMIs
Source: Gigascience. 2018 May 26;7(6):giy059. doi: 10.1093/gigascience/giy059 (PMC6007394; doi:10.1093/gigascience/giy059)
Supplement: Response_to_Reviewer_Comments_Revision_3.pdf [file giy059_response_to_reviewer_comments_revision_3.pdf]

Author's Response To Reviewer Comments

Close

Dear Dr. Zauner,

Thank you for approving the GigaDB submission.

In the attached files, we have updated the citation and structured the abstract as necessary.

Indeed, there seems to be a problem with the compilation of our Latex files in the submission system.
The compilation in Sharelatex works fine for us.
Please do not hesitate to contact us if we should upload the Latex files differently.

Close
