# Supplementary material for: zUMIs - A fast and flexible pipeline to process RNA sequencing data with UMIs
Source: Gigascience. 2018 May 26;7(6):giy059. doi: 10.1093/gigascience/giy059 (PMC6007394; doi:10.1093/gigascience/giy059)
Supplement: Reviewer_1_Report_(Original_Submission) -- Karthik Shekhar — 10/27/2017 Reviewed [file giy059_reviewer_1_report_(original_submission).pdf]

Reviewer Report

Title: zUMIs - A fast and flexible pipeline to process RNA sequencing data with UMIs

Version: Original Submission **Date:** 10/27/2017

Reviewer name: Karthik Shekhar

Reviewer Comments to Author:

Parekh and coworkers introduce a pipeline to process high throughput scRNA-seq data consisting of cell barcodes and UMIs. This is an open-source software that also supports features such as reads downsampling and intron counting - the latter is important for single nucleus RNA-seq data. Overall, this is a useful study and I would like to support its publication, but the current manuscript could greatly benefit with additional biological analysis (related to Fig 4) that would prompt users to take notice. I would like to support its publication, contingent on the authors addressing the following comments. 1. The pipeline appears to collapse only identical UMIs. We have found in our experience that this can lead to overcounting of transcripts, and that it is necessary to collapse UMIs mapping to the same gene in the same cell within an edit distance of 1. I would be curious to see how this impacts the number of transcripts detected per cell. 2. The authors use simulations to describe the impact of intron counting on differential expression. I would instead like to see this on real data. In particular I would like to see examples of "before/after" plots (e.g. violin plots/heatmaps) of specific genes that (1) were called out as differentially expressed (DE) but no longer are once introns are incorporated, (2) the reverse of (1), and (3) those that remain DE but with significantly different statistical significances. 3. I am also not convinced of the result claiming more clusters when introns are included. What is the evidence that these clusters are not spurious? The detection of additional clusters is not evidence enough that these are real. It would be useful to show a heatmap demonstrating that there is true, biologically significant differential expression between the novel clusters detected by the intron counting. 4. For completeness, I would like if the authors could include a section comparing their "exon-counts" matrix with the count matrix produced by either the cellranger or Drop-seq pipeline for datasets that have been classically analyzed by the latter methods. This would produce some confidence in the base reproducibility of the methods. If on the other hand, zUMIs produces a different exon count matrix, then the authors must explain why this is the case. 5. In Figure 4, the authors show to show a confusion matrix to compare how clusters in A map to clusters in B. Also for those clusters that multi-map (i.e. those resolved by intron-exon mapping but not by exon-mapping alone), is there biologically meaningful differential expression? Some examples of specific cell types and their gene expression differences in A vs B would be very informative.

Level of Interest

Please indicate how interesting you found the manuscript: An article whose findings are important to those with closely related research interests

Quality of Written English

Please indicate the quality of language in the manuscript: Acceptable

Declaration of Competing Interests

Please complete a declaration of competing interests, considering the following questions:

- Have you in the past five years received reimbursements, fees, funding, or salary from an organisation that may in any way gain or lose financially from the publication of this manuscript, either now or in the future?
- Do you hold any stocks or shares in an organisation that may in any way gain or lose financially from the publication of this manuscript, either now or in the future?
- Do you hold or are you currently applying for any patents relating to the content of the manuscript?
- Have you received reimbursements, fees, funding, or salary from an organization that holds or has applied for patents relating to the content of the manuscript?
- Do you have any other financial competing interests?
- Do you have any non-financial competing interests in relation to this paper?

If you can answer no to all of the above, write 'I declare that I have no competing interests' below. If your reply is yes to any, please give details below.

I declare that I have no competing interests

I agree to the open peer review policy of the journal. I understand that my name will be included on my report to the authors and, if the manuscript is accepted for publication, my named report including any attachments I upload will be posted on the website along with the authors' responses. I agree for my report to be made available under an Open Access Creative Commons CC-BY license (http://creativecommons.org/licenses/by/4.0/). I understand that any comments which I do not wish to be included in my named report can be included as confidential comments to the editors, which will not be published.

I agree to the open peer review policy of the journal

To further support our reviewers, we have joined with Publons, where you can gain additional credit to further highlight your hard work (see: https://publons.com/journal/530/gigascience). On publication of this paper, your review will be automatically added to Publons, you can then choose whether or not to claim your Publons credit. I understand this statement.

Yes
