# Supplementary material for: zUMIs - A fast and flexible pipeline to process RNA sequencing data with UMIs
Source: Gigascience. 2018 May 26;7(6):giy059. doi: 10.1093/gigascience/giy059 (PMC6007394; doi:10.1093/gigascience/giy059)
Supplement: Reviewer_2_Report_(Original_Submission) -- Keegan Korthauer — 11/21/2017 Reviewed [file giy059_reviewer_2_report_(original_submission).pdf]

Reviewer Report

Title: zUMIs - A fast and flexible pipeline to process RNA sequencing data with UMIs

Version: Original Submission **Date:** 11/21/2017

Reviewer name: Keegan Korthauer

Reviewer Comments to Author:

Review of "zUMIs - A fast and flexible pipeline to process RNA sequencing data with UMIs"Summary:Parekh et al. describe a computational pipeline to preprocess single-cell RNA-seq data that contains UMIs and cell barcodes. The main components of the pipeline include sequence quality filtering of UMIs and barcodes, a wrapper to call the mapping software STAR, selection of cell barcodes, and downsampling of reads to lower library size. While other tools exist that perform all of these steps either all together or individually for one or more platforms, the novelty of zUMIs is that it performs all of these steps at once for data from any UMI platform. Such a tool would likely be useful for the single-cell community, however many methodological details are missing. In addition the manuscript could benefit from additional comparison to existing tools.The authors also argue that in general quantification of gene expression should incorporate intron-mapping reads, a task which is enabled by the use of their software. However, I have reservations about the evidence upon which this conclusion is based. I have identified several issues that the authors should address in order to improve the manuscript, which are detailed below and divided into major (of critical importance) and minor (to improve clarity) categories.Major Comments:1. Methodological details are missing throughout. The method should be described more completely and clearly, and any analyses or comparisons should be made reproducible. For example:* What differential expression method was used in the simulation study to compare UMItools and zUMI? * What options were used with powsimR in the simulation study?* How is the k-dimensional multivariate normal distribution fit in the cell barcode selection step?* How is k determined in the cell barcode selection step?* How was data simulated for the intron evaluation? * What options were used in applying the Seurat pipeline to cluster cells?2. One major conclusion of the paper is that incorporation of intron-mapping reads significantly improves cluster resolution. It is perhaps not surprising that including the intron counts results in a higher mean number of genes detected, but the authors conclude that since more clusters are also found that this means the additional reads are biologically meaningful. Unfortunately, the authors have not provided any evidence that this is the case. The fact that more clusters are seen says nothing about the difference between technical and biological sources of variation. If these additional clusters also corresponded to some independently measured biological covariate, the argument would have basis. 3. Many central conclusions of the article were made based on an analysis of a dataset of 96 cells that is never described. It is referred to as "the HEK dataset" throughout the manuscript, but no citation, details of data generation, or description of the experimental design is given. 4. Several open-source tools exist that perform many of the steps in the zUMI pipeline [1, 2, 3]. It would be nice to see how these perform in comparison to zUMI.5. The conclusion that a UMI distance filter (using UMI-tools) is unnecessary is only based on a single simulated dataset of up to 90 cells per condition. It is also based on a single metric (power to identify differentially expressed genes in simulated data). If we are only interested in differential expression analyses, this might be a reasonable metric. However to be widely applicable to the analysis of single cell RNA-seq, the authors should consider additional metrics such as replicate reproducibility, number of detected genes, etc. The authors should also consider additional datasets. 6. It is not clear how the simulation parameters in the comparison to UMI-tools directly relate to the UMI quantification. Specifically, estimating the mean and dispersion of the processed data and then using these as the basis for a simulated dataset seems pretty far removed from the observed UMI counts. The authors should also investigate differences in differential expression analysis of the actual data (not simulated data). They could also generate a simulated null comparison by randomly permuting sample labels. The same comments hold for the second simulation (evaluating intron count inclusion).Minor Comments:1. The results of the simulation evaluating intron usage are summarized

broadly in the text, but the specific results are not shown. For example what does "power to detect differentially expressed genes was similar for the exon and exon+intron counts" mean? How similar? What were the values? 2. The pipeline requires the user to specify many parameters for each step, however the implementation is run with one command. This means that if a user wants to change a single parameter in one of the later steps, they would still have to rerun the entire pipeline, wasting time and computational resources. It would be useful if the pipeline could alternatively be run as a series of individual steps so that the same exact steps don't need to be carried out multiple times in these situations.3. In the cell barcode selection step, the authors state that they remove "all barcodes that fall in the lower 1% tail of this distribution." What is the justification for this? What does this correspond to in practice? This threshold should also be denoted in Figure 3A.4. What are the practical guidelines for downsampling? How should it be used in practice to normalize for sequencing depth?5. In the documentation online, section on cell barcode selection (here: https://github.com/sdparekh/zUMIs/wiki/Cell-barcodes-selection), Figure A is contradictory to Figure 3A in the manuscript. Specifically, the online documentation says "cells left to the blue line are selected" and the manuscript says "cell barcodes with reads above the blue line are selected." 6. As a main advantage of zUMIs is the ability to apply on any UMI platform, the documentation should clearly state how to use the software in each case. Currently, this is unclear, as for example in the case of the "-c" option the wiki on GitHub (https://github.com/sdparekh/zUMIs/wiki/Usage) states that "For STRT-seq/InDrops give this as 1-n where n is your first cell barcode(-f) length." But it also states in the very next line "For InDrops give this as 1-n where n is the total length of cell barcode(e.g. 1-22)," which is contradictory to what the previous line states about InDrops.References:[1] Luyi Tian, Shian Su, Daniela Amann-Zalcenstein, Christine Biben, Shalin H. Naik, Matthew E. Ritchie. scPipe: a flexible data preprocessing pipeline for single-cell RNA-sequencing data. bioRxiv 175927; doi: https://doi.org/10.1101/175927 [2] Serghei Mangul, Sarah Van Driesche, Lana S. Martin, Kelsey C. Martin, Eleazar Eskin. UMI-Reducer: Collapsing duplicate sequencing reads via Unique Molecular Identifiers. bioRxiv 103267; doi: https://doi.org/10.1101/103267 [3] Viktor Petukhov, Jimin Guo, Ninib Baryawno, Nicolas Severe, David Scadden, Maria G. Samsonova, Peter V. Kharchenko. Accurate estimation of molecular counts in droplet-based single-cell RNA-seq experiments. bioRxiv 171496; doi: https://doi.org/10.1101/171496

Level of Interest

Please indicate how interesting you found the manuscript: An article whose findings are important to those with closely related research interests

Quality of Written English

Please indicate the quality of language in the manuscript: Acceptable

Declaration of Competing Interests

Please complete a declaration of competing interests, considering the following questions:

- Have you in the past five years received reimbursements, fees, funding, or salary from an organisation that may in any way gain or lose financially from the publication of this manuscript, either now or in the future?
- Do you hold any stocks or shares in an organisation that may in any way gain or lose financially from the publication of this manuscript, either now or in the future?

- Do you hold or are you currently applying for any patents relating to the content of the manuscript?
- Have you received reimbursements, fees, funding, or salary from an organization that holds or has applied for patents relating to the content of the manuscript?
- Do you have any other financial competing interests?
- Do you have any non-financial competing interests in relation to this paper?

If you can answer no to all of the above, write 'I declare that I have no competing interests' below. If your reply is yes to any, please give details below.

I declare that I have no competing interests.

I agree to the open peer review policy of the journal. I understand that my name will be included on my report to the authors and, if the manuscript is accepted for publication, my named report including any attachments I upload will be posted on the website along with the authors' responses. I agree for my report to be made available under an Open Access Creative Commons CC-BY license (http://creativecommons.org/licenses/by/4.0/). I understand that any comments which I do not wish to be included in my named report can be included as confidential comments to the editors, which will not be published.

I agree to the open peer review policy of the journal

To further support our reviewers, we have joined with Publons, where you can gain additional credit to further highlight your hard work (see: https://publons.com/journal/530/gigascience). On publication of this paper, your review will be automatically added to Publons, you can then choose whether or not to claim your Publons credit. I understand this statement.

Yes
