# Supplementary material for: zUMIs - A fast and flexible pipeline to process RNA sequencing data with UMIs
Source: Gigascience. 2018 May 26;7(6):giy059. doi: 10.1093/gigascience/giy059 (PMC6007394; doi:10.1093/gigascience/giy059)
Supplement: Reviewer_2_Report_(Revision_1) -- Keegan Korthauer — 3/26/2018 Reviewed [file giy059_reviewer_2_report_(revision_1).pdf]

Reviewer Report

Title: zUMIs - A fast and flexible pipeline to process RNA sequencing data with UMIs

Version: Revision 1 Date: 3/26/2018

Reviewer name: Keegan Korthauer

Reviewer Comments to Author:

Review of Revised version R1 of "zUMIs - A fast and flexible pipeline to process RNA sequencing data with UMIs"

Parekh et al. have partially addressed my concerns, however there remain unresolved issues that have critical importance to the conclusions of the manuscript. In particular, although the authors have included a Methods section with the revision, some methodological details for reproducibility are still missing. In addition, although the authors have compared one aspect of the pipeline to existing tools, other steps of the pipeline that are also carried out by other tools are not compared. Finally, the biological relevance of the differences in clustering with and without introns is still not convincingly demonstrated. These remaining concerns are detailed below (new responses marked by ***).

AUTHOR RESPONSE:
We want to clarify that we do not wish to claim that counting introns is a good idea in general. However, we argue that for extremely sparse datasets such as generated by single nuclei sequencing, having Intron counts is better than losing even more genes. We hope that we could make this clearer in the text, as such:

"Furthermore, we think that although noisy, the large number of additionally detected genes makes Exon+Intron counting worthwhile for extremely sparse data."

***
REVIEWER RESPONSE:
Thank you for the clarification. However, this recommendation remains vague. It would be useful to define what is meant by "extremely sparse data". It may not be clear to the single-cell community, since all single-cell data is quite sparse by nature. But it seems this recommendation is focused on a particular subset of protocols (DroNc-seq)? Please clarify.
***

I have identified several issues that the authors should address in order to improve the manuscript, which are detailed below and divided into major (of critical importance) and minor (to improve clarity) categories.

Major Comments:
1. Methodological details are missing throughout. The method should be described more completely and clearly, and any analyses or comparisons should be made reproducible. For example:
* What differential expression method was used in the simulation study to compare UMItools and zUMI?
* What options were used with powsimR in the simulation study?
* How is the k-dimensional multivariate normal distribution fit in the cell barcode selection step?
* How is k determined in the cell barcode selection step?
* How was data simulated for the Intron evaluation?
* What options were used in applying the Seurat pipeline to cluster cells?

AUTHOR RESPONSE:
We added a methods section (Page:3-4) that includes subsections for (1) data generation of the HEK dataset as well as data processing of other used datasets, (2) the powsimR simulations and (3) the use of the Seurat pipeline. The passage about the Cell-Barcode selection was changed in the main text (Page:2). We hope to have made our barcode selection clearer in the main text.

"To this end, we fit a k-dimensional multivariate normal distribution using the R-package mclust [25, 26] for the number of reads/BC, where k is empirically determined by mclust via the Bayesian Information Criterion (BIC). We reason that only the kth normal distribution with the largest mean contains barcodes that identify reads originating from intact cells."

***
REVIEWER RESPONSE:
The authors added a Methods section. Most of the details seem to have been added, but there are still some details that I have questions about. For example, how was the Intron-sampling experiment carried out (Figure 1 in Additional File 1)? What is meant by "sufficiently many cells for DE analysis" (page 3)?
***

2. One major conclusion of the paper is that incorporation of intron-mapping reads significantly improves cluster resolution. It is perhaps not surprising that including the Intron counts results in a higher mean number of genes detected, but the authors conclude that since more clusters are also found that this means the additional reads are biologically meaningful. Unfortunately, the authors have not provided any evidence that this is the case. The fact that more clusters are seen says nothing about the difference between technical and biological sources of variation. If these additional clusters also corresponded to some independently measured biological covariate, the argument would have basis.

AUTHOR RESPONSE:
While we do not wish to claim that counting of intron-mapping reads is recommended in all cases of scRNA-seq, we do think it is valid and helpful for extremely sparse datasets such as the DroNc-seq data from Habib et al. (2017). We now provide detailed analyses of differences between newly formed subclusters using Exon+Intron counting. We find not only more genes, but also more significantly differentially expressed genes between subclusters when using Exon+Intron UMI data (Figure 4D). Furthermore, log2 fold changes (LFC) for the groups that were split up more when using Exon+Intron counting corresponded well to the Exon-only LFC (see the new Figure 4F). Additionally, we illustrate the biological relevance of subclusters found with Exon+Intron data by the example of the transcriptomic subtypes of GABAergic Pvalb-type Neurons marked by Il1rapl2 expression. We have added this evidence to the 'Intron Counting' section and included methodological details in the appropriate Methods sections.
Lastly, we have excluded the possibility of Intron-mapping reads being spurious by sampling fake intronic reads and attempting cluster identification (see response to Reviewer 1, point 3).

***
REVIEWER RESPONSE:
The authors have not fully addressed the concern of biologically meaningful clustering results. They highlight the example of a single gene, which is not convincing that the results are systematically meaningful. In main text they state that 5% of the additional genes found are marker genes, but no baseline is given to be able to judge if that is a significant result. What percentage of genes overall are marker genes? What percentage of DE genes by exon only are marker genes?

Furthermore while they have shown that there are more DE genes between sub-clusters with introns included (again this is not surprising since more genes are detected), this result could be influenced to a

degree by the use of a model (limma-trend) that is not appropriate for sparse RNA-seq data (see also response to Major comment 6).

In addition, it seems that the figures or additional results presented in the Additional File (including the "sampling fake intronic reads") are never mentioned in the manuscript. To me, the sampling fake intronic reads analysis could be a valuable addition to supporting the conclusion that utilizing the intronic reads gives biologically meaningful clustering results (as long as the details of this simulation are realistic, but these details are currently not provided).

As an additional note, it is not clear what color represents in newly added Figures 4D, G, and H and this is not defined in the legend. It is also not clear what color represents in Figure 4A and B - some colors seem to map to the legend of 4C, but not all of them.
***

3. Many central conclusions of the article were made based on an analysis of a dataset of 96 cells that is never described. It is referred to as "the HEK dataset" throughout the manuscript, but no citation, details of data generation, or description of the experimental design is given.

AUTHOR RESPONSE:
We added this information to the new Method section (Page:3-4).

***
REVIEWER RESPONSE
The authors have addressed this concern.
***

4. Several open-source tools exist that perform many of the steps in the zUMI pipeline [1, 2, 3]. It would be nice to see how these perform in comparison to zUMI.

AUTHOR RESPONSE:
While several tools exist that can perform some of the steps of the zUMIs pipeline, none of them provides a comprehensive combination as zUMIs. We have added a Table to compare available features of six other pipelines geared towards scRNA-seq data with UMIs. The tool "UMI-Reducer" with reference [2] suggested by the reviewer was omitted because it seemed like a tool geared towards one specific application outside of single-cell RNA-seq. Furthermore, "UMI-Reducer" only de-duplicates UMIs with the same mapping position, which would be inappropriate for scRNA-seq protocols that fragment after preamplification, such as SCRB-seq.
Furthermore, we added a comparison of the count-tables produced by zUMIs, Drop-seq-tools and UMI-tools and generally find very good correspondence (see response to Reviewer 1).

***
REVIEWER RESPONSE
The authors have included a comparison to two other tools for the UMI-collapsing strategy. However, these other tools are not included in any other aspect. For example, how do other methods perform in cell barcode selection (Page 2)? How do other methods perform in running time (Figure 3E)?
***

5. The conclusion that a UMI distance filter (using UMI-tools) is unnecessary is only based on a single simulated dataset of up to 90 cells per condition. It is also based on a single metric (power to identify differentially expressed genes in simulated data). If we are only interested in differential expression

analyses, this might be a reasonable metric. However to be widely applicable to the analysis of single cell RNA-seq, the authors should consider additional metrics such as replicate reproducibility, number of detected genes, etc. The authors should also consider additional datasets.

AUTHOR RESPONSE:
We substantially extended our comparison of different UMI-collapsing method. In Fig. 2 B,C, we also compare the correlation of gene expression values and numbers of detected UMIs per cell between various different filtering methods and find that there is generally a high consensus among all UMI collapsing methods in our HEK example dataset. An analysis of the DroNc-seq data gave basically the same results (see plot in attached "Additional File 1").
Furthermore, we added the possibility to collapse UMIs with a specified Hamming-distance to zUMIs, giving users more choice over UMI filtering. All these new analysis are also described in the section "Transcript Counting" of the main text.

***
REVIEWER RESPONSE
The authors have addressed this concern.
***

6. It is not clear how the simulation parameters in the comparison to UMI-tools directly relate to the UMI quantification. Specifically, estimating the mean and dispersion of the processed data and then using these as the basis for a simulated dataset seems pretty far removed from the observed UMI counts. The authors should also investigate differences in differential expression analysis of the actual data (not simulated data). They could also generate a simulated null comparison by randomly permuting sample labels. The same comments hold for the second simulation (evaluating Intron count inclusion).

AUTHOR RESPONSE:
We removed the simulations from the description of UMI-collapsing methods and focus our reporting on the descriptive statistics suggested by the reviewer (Figure 2 & section "Transcript counting").

***
REVIEWER RESPONSE
The authors have removed one of the simulations from the manuscript, but the second simulation remains. Unfortunately, I still have concerns regarding the intron count inclusion simulation. In particular, now that the authors have added methodological details, they have used a differential expression method that was developed for bulk RNA-seq and is not appropriate for sparse single-cell RNA-seq data. It could be that the differential expression results are due to a poorly fitting model with more sparse exon-only data. The authors should use a method appropriate for sparse single-cell RNA-seq data, such as MAST (Finak et al. 2015, Genome Biology) or zingeR (Van den Berge et al. 2018, Genome Biology).
***

Minor Comments:
1. The results of the simulation evaluating Intron usage are summarized broadly in the text, but the specific results are not shown. For example what does "power to detect differentially expressed genes was similar for the Exon and Exon+Intron counts" mean? How similar? What were the values?

AUTHOR RESPONSE:
This is now better described in the main text (Page 3 Passage:Intron Counting) along with specific settings for the powsimR package listed in the method section. Additionally, power simulation results are shown in

Figure 4 with the true positive rate (TPR) and false discovery rate (FDR) shown for 5 stratas of gene expression (Figure 4G). Furthermore, we display the number of genes per stratum for Exon and Exon+Intron counting (Figure 4H).

***
REVIEWER RESPONSE
The authors have added simulation results and addressed this concern.
***

2. The pipeline requires the user to specify many parameters for each step, however the implementation is run with one command. This means that if a user wants to change a single parameter in one of the later steps, they would still have to rerun the entire pipeline, wasting time and computational resources. It would be useful if the pipeline could alternatively be run as a series of individual steps so that the same exact steps don't need to be carried out multiple times in these situations.

AUTHOR RESPONSE:
This feature is implemented as "-w" option. One can invoke zUMIs at any step, eg to just re-run the counting of gene expression the user can give "-w counting".

***
REVIEWER RESPONSE
The authors have addressed this concern.
***

3. In the cell barcode selection step, the authors state that they remove "all barcodes that fall in the lower 1% tail of this distribution." What is the justification for this? What does this correspond to in practice? This threshold should also be denoted in Figure 3A.

AUTHOR RESPONSE:
The blue line in figure 3A corresponds to the calculated read cut-off. The normal distribution identified by mclust with the highest mean number of reads contains actual cell barcodes. Thus, setting the read cut-off to the lower 1% of this distribution is an empirical value that gives good correspondence to the known cell-barcodes for the HEK dataset (cut-off value: 52634 reads/barcode) and gave similarly good results for the DroNc-seq data analysed here. Still, in practice we recommend to always look at the elbow-plots output by zUMIs (Figure 3B). This will show whether our empirical cut-off was also valid for the dataset at hand.

***
REVIEWER RESPONSE
It would be useful to state your recommendation in the manuscript "to always look at the elbow-plots output by zUMIs (Figure 3B)" when setting the cut-off so that readers can benefit from this advice.
***

4. What are the practical guidelines for downsampling? How should it be used in practice to normalize for sequencing depth?

AUTHOR RESPONSE:
We found the downsampling function extremely useful for method comparisons as we showed in our previous study (Ziegenhain et al. 2017). This also allows to evaluate whether the single cell libraries were

sequenced to saturation (Figure 3D). For normalization purposes, the built-in MAD cut-offs as indicated by the dashed red lines in Figure 3C should be sufficient.

***
REVIEWER RESPONSE
The authors have addressed this concern.
***

5. In the documentation online, section on cell barcode selection (here: https://github.com/sdparekh/zUMIs/wiki/Cell-barcodes-selection), Figure A is contradictory to Figure 3A in the manuscript. Specifically, the online documentation says "cells left to the blue line are selected" and the manuscript says "cell barcodes with reads above the blue line are selected."

AUTHOR RESPONSE:
This was indeed a mistake and we corrected it on GitHub.

***
REVIEWER RESPONSE
The authors have addressed this concern.
***

6. As a main advantage of zUMIs is the ability to apply on any UMI platform, the documentation should clearly state how to use the software in each case. Currently, this is unclear, as for example in the case of the "-c" option the wiki on GitHub (https://github.com/sdparekh/zUMIs/wiki/Usage) states that "For STRT-seq/InDrops give this as 1-n where n is your first cell barcode(-f) length." But it also states in the very next line "For InDrops give this as 1-n where n is the total length of cell barcode(e.g. 1-22)," which is contradictory to what the previous line states about InDrops.

AUTHOR RESPONSE:
This was indeed a mistake and we corrected it on GitHub.

***
REVIEWER RESPONSE
The authors have addressed this concern.
***

References:
[1] Luyi Tian, Shian Su, Daniela Amann-Zalcenstein, Christine Biben, Shalin H. Naik, Matthew E. Ritchie. scPipe: a flexible data preprocessing pipeline for single-cell RNA-sequencing data. bioRxiv 175927; doi: https://doi.org/10.1101/175927

[2] Serghei Mangul, Sarah Van Driesche, Lana S. Martin, Kelsey C. Martin, Eleazar Eskin. UMI-Reducer: Collapsing duplicate sequencing reads via Unique Molecular Identifiers. bioRxiv 103267; doi: https://doi.org/10.1101/103267

[3] Viktor Petukhov, Jimin Guo, Ninib Baryawno, Nicolas Severe, David Scadden, Maria G. Samsonova, Peter V. Kharchenko. Accurate estimation of molecular counts in droplet-based single-cell RNA-seq experiments. bioRxiv 171496; doi: https://doi.org/10.1101/171496

Level of Interest

Please indicate how interesting you found the manuscript: An article whose findings are important to those with closely related research interests

Quality of Written English

Please indicate the quality of language in the manuscript: Acceptable

Declaration of Competing Interests

Please complete a declaration of competing interests, considering the following questions:

- Have you in the past five years received reimbursements, fees, funding, or salary from an organisation that may in any way gain or lose financially from the publication of this manuscript, either now or in the future?
- Do you hold any stocks or shares in an organisation that may in any way gain or lose financially from the publication of this manuscript, either now or in the future?
- Do you hold or are you currently applying for any patents relating to the content of the manuscript?
- Have you received reimbursements, fees, funding, or salary from an organization that holds or has applied for patents relating to the content of the manuscript?
- Do you have any other financial competing interests?
- Do you have any non-financial competing interests in relation to this paper?

If you can answer no to all of the above, write 'I declare that I have no competing interests' below. If your reply is yes to any, please give details below.

I declare that I have no competing interests

I agree to the open peer review policy of the journal. I understand that my name will be included on my report to the authors and, if the manuscript is accepted for publication, my named report including any attachments I upload will be posted on the website along with the authors' responses. I agree for my report to be made available under an Open Access Creative Commons CC-BY license (http://creativecommons.org/licenses/by/4.0/). I understand that any comments which I do not wish to be included in my named report can be included as confidential comments to the editors, which will not be published.

I agree to the open peer review policy of the journal

To further support our reviewers, we have joined with Publons, where you can gain additional credit to further highlight your hard work (see: https://publons.com/journal/530/gigascience). On publication of this paper, your review will be automatically added to Publons, you can then choose whether or not to claim your Publons credit. I understand this statement.

Yes
