# Supplementary material for: zUMIs - A fast and flexible pipeline to process RNA sequencing data with UMIs
Source: Gigascience. 2018 May 26;7(6):giy059. doi: 10.1093/gigascience/giy059 (PMC6007394; doi:10.1093/gigascience/giy059)
Supplement: Reviewer_2_Report_(Revision_2) -- Keegan Korthauer — 4/19/2018 Reviewed [file giy059_reviewer_2_report_(revision_2).pdf]

Reviewer Report

Title: zUMIs - A fast and flexible pipeline to process RNA sequencing data with UMIs

Version: Revision 2 Date: 4/19/2018

Reviewer name: Keegan Korthauer

Reviewer Comments to Author:

Review of Revised version R2 of "zUMIs - A fast and flexible pipeline to process RNA sequencing data with UMIs" Parekh et al. have adequately addressed all concerns except the following minor clarifications detailed below: issue number 1 regarding methodological details and issue number 2 regarding the increase of cluster resolution and biological information (concerns that have been adequately addressed are omitted below). 1. Methodological details are missing throughout. The method should be described more completely and clearly, and any analyses or comparisons should be made reproducible. For example: * What differential expression method was used in the simulation study to compare UMItools and zUMI? * What options were used with powsimR in the simulation study? * How is the k-dimensional multivariate normal distribution fit in the cell barcode selection step? * How is k determined in the cell barcode selection step? * How was data simulated for the Intron evaluation? * What options were used in applying the Seurat pipeline to cluster cells? --- AUTHOR RESPONSE: We added a methods section (Page:3-4) that includes subsections for (1) data generation of the HEK dataset as well as data processing of other used datasets, (2) the powsimR simulations and (3) the use of the Seurat pipeline. The passage about the Cell-Barcode selection was changed in the main text (Page:2). We hope to have made our barcode selection clearer in the main text. "To this end, we fit a k-dimensional multivariate normal distribution using the R-package mclust [25, 26] for the number of reads/BC, where k is empirically determined by mclust via the Bayesian Information Criterion (BIC). We reason that only the kth normal distribution with the largest mean contains barcodes that identify reads originating from intact cells." --- *** REVIEWER RESPONSE: The authors added a Methods section. Most of the details seem to have been added, but there are still some details that I have questions about. For example, how was the Intron-sampling experiment carried out (Figure 1 in Additional File 1)? What is meant by "sufficiently many cells for DE analysis" (page 3)? *** --- AUTHOR RESPONSE 2: We now added a description of the Intron-Sampling to Clustering part of the Methods section. Sorry, for the confusion about the statement "sufficiently many cells ..." - In this analysis we included all genes that were detected, i.e. had at least one read mapped. We changed this sentence. "For a fair comparison, we include all detected genes". --- ***REVIEWER RESPONSE 2:Please clarify "all detected genes". Is this all genes detected in Exon or Exon+Intron? If including all detected genes, then why are there different numbers of genes in each quantile in Figure 4H (top panel)? By definition, a quantile bin should contain roughly equal numbers of observations, but this does not seem to be the case even if the quantiles were defined on the Exon+Intron data (the larger quantile bins have more genes for Exon+Intron).***2. One major conclusion of the paper is that incorporation of intron-mapping reads significantly improves cluster resolution. It is perhaps not surprising that including the Intron counts results in a higher mean number of genes detected, but the authors conclude that since more clusters are also found that this means the additional reads are biologically meaningful. Unfortunately, the authors have not provided any evidence that this is the case. The fact that more clusters are seen says nothing about the difference between technical and biological sources of variation. If these additional clusters also corresponded to some independently measured biological covariate, the argument would have basis. --- AUTHOR RESPONSE: While we do not wish to claim that counting of intron-mapping reads is recommended in all cases of scRNA-seq, we do think it is valid and helpful for extremely sparse datasets such as the DroNc-seq data from Habib et al. (2017). We now provide detailed analyses of differences between newly formed subclusters using Exon+Intron counting. We find not only more genes, but also more significantly differentially expressed genes between subclusters when using Exon+Intron UMI data (Figure 4D). Furthermore, log2 fold changes (LFC) for the groups that were split up more when using Exon+Intron counting corresponded well to the Exon-only LFC (see the new Figure 4F).

Additionally, we illustrate the biological relevance of subclusters found with Exon+Intron data by the example of the transcriptomic subtypes of GABAergic Pvalb-type Neurons marked by Il1rapl2 expression. We have added this evidence to the 'Intron Counting' section and included methodological details in the appropriate Methods sections. Lastly, we have excluded the possibility of Intron-mapping reads being spurious by sampling fake intronic reads and attempting cluster identification (see response to Reviewer 1, point 3). --- *** REVIEWER RESPONSE: The authors have not fully addressed the concern of biologically meaningful clustering results. They highlight the example of a single gene, which is not convincing that the results are systematically meaningful. In main text they state that 5% of the additional genes found are marker genes, but no baseline is given to be able to judge if that is a significant result. What percentage of genes overall are marker genes? What percentage of DE genes by exon only are marker genes? Furthermore while they have shown that there are more DE genes between sub-clusters with introns included (again this is not surprising since more genes are detected), this result could be influenced to a degree by the use of a model (limma-trend) that is not appropriate for sparse RNA-seq data (see also response to Major comment 6). In addition, it seems that the figures or additional results presented in the Additional File (including the "sampling fake intronic reads") are never mentioned in the manuscript. To me, the sampling fake intronic reads analysis could be a valuable addition to supporting the conclusion that utilizing the intronic reads gives biologically meaningful clustering results (as long as the details of this simulation are realistic, but these details are currently not provided). As an additional note, it is not clear what color represents in newly added Figures 4D, G, and H and this is not defined in the legend. It is also not clear what color represents in Figure 4A and B - some colors seem to map to the legend of 4C, but not all of them. *** --- AUTHOR RESPONSE 2: We extended the section where we cite a number of papers that utilize intron counts in the analysis of single cell RNA-seq and in particular single nuclei sequencing data. What information can be gained from intron counting is indeed a hot topic in the single cell community and even though we believe to contribute some evidence to support the notion that intron counts add biologically meaningful information, our paper is hardly the place where this issue can be fully resolved. However, the intron counting utility of zUMIs will facilitate research in this area and thus ultimately help other researchers to address this question. Concerning the additionally detected marker genes: We do not expect any enrichment of marker genes when we use Exon+Intron counting and the 5% additional markers are roughly the level expected: 4 % of all detected Exon+Intron genes are also marker genes. However, detecting more of them gives us a better chance to later classify the cells. We also agree that it is not surprising that we find more DE genes if we detect more genes overall. Including introns simply allows us to better detect present transcripts. On the contrary, we would be reluctant to recommend the inclusion of Introns if they would lead to a major shift in the expression profiles, i.e. many more DE- or marker genes than expected. We now added the fake intron analysis to Figure 4 and also extended the methods section for Cluster Identification to describe our sampling: "To illustrate that the additional clusters found by counting Exon+Intron reads are not spurious, we use Intron-only UMI-counts from the same data to add to the observed Exon only counts. More specifically, to each gene we add scran-sizeFactor corrected Intron counts from the same gene after permuting them across cells. We assessed the cluster numbers from 100 such permutations." We now explain the color schemes of Figure 4 A,B &E in the figure legend and added a color legend for D,H &I (formerly D,G & H). "Different shades of those clusters indicate that multiple clusters had the same major cell-type assigned." --- *** REVIEWER RESPONSE 2: Thanks for the clarification regarding enrichment of marker genes as opposed to simply detecting more genes in general. I appreciate the explanation given by the paragraph in the authors' response beginning with "Concerning the additionally detected marker genes:...". It would be transparent to include this explanation in the manuscript, since as it reads currently, it seems to imply an enrichment for marker genes when including introns. ***

Level of Interest

Please indicate how interesting you found the manuscript: Choose an item.

Quality of Written English

Please indicate the quality of language in the manuscript: Choose an item.

Declaration of Competing Interests

Please complete a declaration of competing interests, considering the following questions:

- Have you in the past five years received reimbursements, fees, funding, or salary from an organisation that may in any way gain or lose financially from the publication of this manuscript, either now or in the future?
- Do you hold any stocks or shares in an organisation that may in any way gain or lose financially from the publication of this manuscript, either now or in the future?
- Do you hold or are you currently applying for any patents relating to the content of the manuscript?
- Have you received reimbursements, fees, funding, or salary from an organization that holds or has applied for patents relating to the content of the manuscript?
- Do you have any other financial competing interests?
- Do you have any non-financial competing interests in relation to this paper?

If you can answer no to all of the above, write 'I declare that I have no competing interests' below. If your reply is yes to any, please give details below.

I declare that I have no competing interests.

I agree to the open peer review policy of the journal. I understand that my name will be included on my report to the authors and, if the manuscript is accepted for publication, my named report including any attachments I upload will be posted on the website along with the authors' responses. I agree for my report to be made available under an Open Access Creative Commons CC-BY license (http://creativecommons.org/licenses/by/4.0/). I understand that any comments which I do not wish to be included in my named report can be included as confidential comments to the editors, which will not be published.

I agree to the open peer review policy of the journal

To further support our reviewers, we have joined with Publons, where you can gain additional credit to further highlight your hard work (see: https://publons.com/journal/530/gigascience). On publication of this paper, your review will be automatically added to Publons, you can then choose whether or not to claim your Publons credit. I understand this statement.

Yes
